# The association between serum lipid levels and colorectal cancer risk: A dose-response meta-analysis of 23 studies

**DOI:** 10.1371/journal.pone.0333907

**Published:** 2025-10-16

**Authors:** Iman Elahi Vahed, Zahra Esmaili, Mina Pourhabib Mamaghani, Shohreh Farshid, Behina Salarian, Mobina Alamdari, Zahra Azimizadeh, Zahra Rastad, Hoda Radmard, Hossein Soltaninejad, Mohammad Rahmanian

**Affiliations:** 1 School of Medicine, Shahid Beheshti University of Medical Sciences, Tehran, Iran; 2 Shiraz University of Medical Sciences, Shiraz, Iran; 3 Medical Physics Research Center, Mashhad University of Medical Sciences, Mashhad, Iran; 4 Student Reaserch Committee, Mashhad University of Medical Science, Mashhad, Iran; 5 University of Gothenburg, Gothenburg, Sweden; 6 Medical Campus, Xi’an Jiaotong University, Xi’an, Shaanxi, China; 7 Tehran Medical Science, Islamic Azad University, Tehran, Iran; 8 Shahid Sadoughi University of Medical Sciences, Yazd, Iran; 9 School of Medicine, Zahedan University of Medical Sciences, Zahedan, Iran; 10 Birjand University of Medical Sciences, Birjand, Iran; 11 Department of stem cells technology and Tissue Regeneration, Faculty of Interdisciplinary Science and Technologies, Tarbiat Modares University, Tehran, Iran; 12 Gastroenterology and Liver Diseases Research Center, Research Institute for Gastroenterology and Liver Diseases, Shahid Beheshti University of Medical Sciences, Tehran, Iran; 13 Student Research Committee, School of Medicine, Shahid Beheshti University of Medical Sciences, Tehran, Iran; Athens Medical Group, Psychiko Clinic, GREECE

## Abstract

**Background:**

Colorectal cancer (CRC) ranks as the third most prevalent cancer globally and the second leading cause of cancer-related mortality. Based on recent studies, lipid levels may have a relationship with the risk of CRC. This meta-analysis aims to better understand the association between various serum lipids and CRC risk.

**Methods:**

A comprehensive search was conducted in Web of Science, PubMed, and Scopus. This meta-analysis, including only prospective cohort studies, performed random-effects meta-analyses using the Restricted Maximum Likelihood (REML) model to assess the association between the highest versus lowest categories of serum triglycerides (TG), total cholesterol (TC), high-density lipoprotein (HDL), and low-density lipoproteins (LDL) with the risk of CRC, primarily using hazard ratios (HR) as the effect size. Subgroup analyses (e.g., by tumor site, region, and risk of bias) and meta-regression analyses (e.g., for mean age, mean BMI, sex distribution, and duration of follow-up) were conducted to explore heterogeneity. Dose-response analyses were performed utilizing three model types.

**Results:**

Following the screening of 27,278 articles, 23 articles have been included in this study finally. The associations between TG, TC, HDL, and LDL levels and the risk of CRC, colon, and rectum cancers were examined separately. Higher levels of TC were not significantly associated with the risk of CRC (HR = 1.08; 95% CI: 0.90–1.30; I^2^ = 50.55%; p = 0.4187) and colon cancer (HR = 1.08; 95% CI: 0.99–1.18; I^2^ = 35.57%; p = 0.0720), but were significantly associated with an increased risk of rectum cancer (HR = 1.19; 95% CI: 1.08–1.32; I^2^ = 28.36%; p = 0.0004). Higher levels of TG were associated with an increased hazard of CRC (HR 1.11; 95% CI: 1.044–1.18; I^2^ = 0.0%; p = 0.0008). For colon cancer, TG showed a marginally significant association (HR 1.23; 95% CI: 0.99–1.55; I^2^ = 52.0%; p = 0.0576). No significant association was found between TG levels and rectum cancer risk (HR 1.036; 95% CI: 0.69–1.56; I^2^ = 67.29%; p = 0.8674).Also, higher levels of HDL were not significantly associated with the risk of CRC (HR 0.93; 95% CI: 0.83–1.03; I^2^ = 28.8%; p = 0.1527), colon cancer (HR 0.94; 95% CI: 0.75–1.19; I^2^ = 0.0%; p = 0.6243), and rectum cancer (HR: 0.95; 95% CI: 0.66;1.37; I^2^ = 0.0%; p = 0.7888). For colon cancer, higher LDL level was not significantly associated with risk (HR 0.91; 95% CI: 0.60–1.37; p = 0.21; I^2^ = 37%; p = 6558).

Accordingly quadratic and RCS models represented as lowest AIC for TC (p = 0.026), for TG (p = 0.004), for LDL (p = 0.942) and for HDL (p = 0.295).

**Conclusion:**

Higher TG level was significantly associated with increased risk of CRC and showed a borderline association with colon cancer (HR 1.23, 95% CI 0.99–1.55; p = 0.0576), while TC, HDL, and LDL showed no significant associations with these cancers. For rectum cancer, higher TC was significantly linked to increased risk, whereas TG, HDL, and LDL showed no significant associations. Future research should prioritize longitudinal studies to investigate the mechanistic roles of hormones and the gut microbiota in modulating colorectal cancer risk, alongside multi-omics studies that integrate lipid metabolism with other biological variables such as inflammatory markers and genetic predispositions. These efforts could clarify causal pathways and inform targeted prevention strategies.

## Introduction

Globally, colorectal cancer (CRC) is the third most frequently diagnosed cancer and the second leading cause of cancer-related mortality, causing more than 930,000 deaths. Approximately two million new cases of CRC were identified in 2020 [1,2]. It is believed that 25–30 percent of CRC cases are attributed to non-modifiable risk factors [3]. These risk factors include genetic predispositions, a personal history of adenomas or polyps, and a family history of CRC or genetic risks. However, 70–75% of cases may be linked to modifiable risk factors such as alcohol use, smoking, unhealthy dietary patterns, and physical inactivity, obesity and dyslipidemia [4].

Dyslipidemia is a kind of metabolic disorder which may be genetically determined but can also be attributed to the diet, physical inactivity, and certain diseases [5]. It is one of the leading causes of heart issues, strokes, nonalcoholic fatty liver disease (NAFLD), and diabetes [6–9].

Also, some evidence shows high triglycerides (TG) and lipid levels linked to a higher risk of gastric, liver, esophageal, and ovarian cancer [10–13].

Some studies have reported raised lipid levels, specifically TG and total cholesterol (TC), have been connected to a higher risk of CRC. Cholesterol, a major component of cell membranes, plays a role in cell signaling, migration, and survival by regulating the stability, permeability, and formation of specialized microstructures called lipid rafts in the membrane [14]. These microdomains, particularly in cancer cells, are the site of accumulation of growth factor receptors and other regulatory proteins that can activate cell proliferation pathways such as PI3K/Akt and MAPK and contribute to tumor progression [15]. In addition, cholesterol plays a role in the growth and survival of cancer cells by disrupting cell signaling mechanisms [16].

In addition, TG also plays an important role in tumorigenesis by inducing insulin resistance. TG accumulation in the liver and peripheral tissues, by activating pathways such as PKC and JNK, disrupts insulin receptor phosphorylation and reduces cell sensitivity to insulin [17]. This condition leads to increased serum insulin and Insulin-like Growth Factor-1(IGF-1), molecules that facilitate neoplastic growth through cell growth and survival pathways, such as Akt/mTOR [18].

However, some researchers have claimed the protective impact high-density lipoprotein (HDL) for CRC risk [19]. Dyslipidemia may play a role in CRC through pathways of chronic inflammation and oxidative stress [20].

Immune dysregulation, redox reactions, and protein dysfunction can result from elevated plasma cholesterol levels. These disturbances reduce tumor apoptosis while promoting angiogenesis and cellular growth. Interleukin-6 (IL-6) and tumor necrosis factor-α (TNF-α) serve as two examples of inflammatory mediators that can rise in response to decreased HDL and increased low-density lipoprotein (LDL) and TC (5). Oxidized lipoproteins, such as oxidized LDL, can stimulate the innate immune response through the activation of macrophages and NF-κB pathways and increase the production of proinflammatory cytokines such as TNF-α and IL-6 [21].

Numerous epidemiological studies have examined the association between dyslipidemia and CRC risk, but the results obtained from these studies are often heterogeneous and contradictory. In this context, Fränzel J. B. van Duijnhoven et al. conducted a study within the framework of the European Prospective Investigation into Cancer and Nutrition (EPIC) to examine the associations between blood levels of HDL, LDL, triglycerides, apolipoprotein A-I (apoA), and apolipoprotein B with the risk of colorectal cancer. The results indicated that higher concentrations of HDL were associated with a decreased risk of CRC, suggesting a potential protective role of HDL due to its anti-inflammatory and antioxidant properties [22]. However, Gemma Ibáñez-Sanz et al., using Mendelian randomization, demonstrated that lipid levels such as HDL, TG, and LDL are not causally associated with the risk of colorectal cancer [23]. Given the conflicting results reported in previous studies, investigating the relationship between serum lipid levels and CRC remains essential and warrants further research.

This meta-analysis evaluates the dose-response relationship between HDL, LDL, TC, and TG and the risk of CRC development.

## Materials and methods

This systematic review and meta-analysis was reported in accordance with the PRISMA 2020 guidelines. The study protocol was also submitted and registered in the International Prospective Register of Systematic Reviews (PROSPERO) under the registration ID CRD42024612076 (See Appendix).

### Search strategy

We explored the databases PubMed, Scopus and Web of Science to find articles reporting the association between the levels of lipids in the serum and CRC, published up to 21 September 2024. The search applied the following MeSH terms: “Colorectal Neoplasms” and “Lipids/blood,” “Cholesterol,” “Triglycerides/blood,” “Cholesterol, HDL,” “Cholesterol, LDL,” “Cholesterol, VLDL,” “Hyperlipidemias,” and “Dyslipidemias.” A detailed search strategy is provided in S1 Table in S3 File. Backward and forward citation-seeking methods were applied to ensure the accuracy of the search. The references of selected articles were reviewed by backward citation to find further suitable studies that had a risk of being missed from the beginning. By employing forward citation, recently published papers that cited the selected studies since they were published, were identified.

### Study selection

All search results were combined using EndNote X9 (Clarivate Analytics, Philadelphia, PA, USA). Each paper’s title, abstract, and full text were examined by two reviewers (S.F. and S.F.) separately to exclude non-relevant studies. A third reviewer (M.R.) resolved the disagreements. The study followed the PECO format for identifying and analyzing the relationship between serum lipid levels and the risk of CRC. (P) The population of interest included individuals diagnosed with CRC. (E) We aimed to evaluate the relationship between higher levels of serum lipids—specifically TG, LDL, HDL and TC—and CRC risk, (C) comparing them to lower serum lipid levels. (O) The primary outcome was to determine the relationship between different levels of serum lipids and the risk of obtaining CRC. We included only cohort studies that examined the association between serum lipid levels and colorectal cancer incidence. Eligible studies were required to report effect estimates as hazard ratios (HRs) or risk ratios (RRs) with corresponding 95% confidence intervals. Studies using other designs, such as case-control or cross-sectional studies, were excluded. Articles from conferences or editorial articles, review studies, protocols, in-vivo or in-vitro studies, and papers not examining serum lipids in CRC patients were excluded from the study.

### Data extraction

A standard data extraction form was designed by I.E and M.R. Any differences of opinion among the reviewers were settled through mutual agreement. The following data has been extracted: type of study, location of study, data on population demographics (age and gender), size of the sample, first name of author, publication date, funding source (s), duration of study, follow-up duration, type of serum lipids (i.e., TG, LDL, HDL), measuring method, the number and definition of case and controls, possible confounding factors and their adjustments.

### Risk of bias evaluation

The risk of bias assessment of the included studies was conducted by two reviewers (H.R. and S.F) by Joanna Briggs Institute (JBI) Critical Appraisal Tools [24]. Conflicts were resolved by a third reviewer (M.R.).

These items were studied: demographic details of participants, exposure along with outcome assessments, confounding factor identification and correction, follow-up data, and appropriateness of statistical analysis. The JBI scores for the included studies ranged from 6/11–11/11.

### Statistical analysis

We performed random-effects meta-analyses to evaluate the associations between serum lipid levels including total cholesterol, triglycerides, high-density lipoprotein, and low-density lipoprotein and the risk of colorectal cancer. All analyses were conducted as high vs. low comparisons, where the highest exposure category was compared to the lowest, to ensure consistency across studies with varying exposure groupings (e.g., dichotomies, tertiles, quartiles, or quintiles). For studies that did not use the lowest exposure category as the reference group, we recalculated effect estimates to ensure consistent comparisons between the highest and lowest categories (e.g., Q5 vs. Q1). This was done using logarithmic transformation of the reported HRs and applying the identity:


log(HRQ5vsQ1) = log(HRQ5vsQ3) – log(HRQ1vsQ3),

with standard errors and confidence intervals computed accordingly. Full details of the transformation procedure are provided in the Supplementary Material.

The primary summary measure was the hazard ratio (HR), given that most included studies were prospective cohort designs. For studies that reported relative risks (RRs) instead of HRs, we considered RRs as equivalent to HRs due to the typically low incidence rate of CRC in the general population and the similarity of interpretations under this context. This approach is supported by previous methodological guidance indicating the comparability of HRs and RRs in meta-analyses of time-to-event outcomes when incidence is low [25].

Random-effects models were fitted using the restricted maximum likelihood (REML) estimator to account for potential between-study heterogeneity. Statistical heterogeneity was assessed using Cochran’s Q test and quantified with the I^2^ statistic. Publication bias was evaluated using Egger’s regression test, and statistical significance was defined as a two-sided p-value < 0.05.

To explore potential sources of heterogeneity in the meta-analysis, we conducted multiple subgroup analyses. These included stratification by tumor site, geographic region, risk of bias scores, lipid status, exclusion of participants with pre-existing cardiovascular disease or diabetes mellitus at baseline, and exclusion of CRC cases identified during early follow-up periods.

In addition, we performed meta-regression analyses to evaluate the potential modifying effects of continuous study-level variables. These moderators included the proportion of male participants, mean age, mean body mass index (BMI), and duration of follow-up. These analyses allowed us to assess whether variations in participant characteristics or study design features contributed to differences in effect estimates across studies.

To evaluate the robustness of findings, sensitivity analyses were conducted for outcomes with substantial heterogeneity (I^2^ > 50%) by sequentially excluding each study (leave-one-out analysis) and by restricting analyses to studies at low risk of bias.

For the dose-response relationship between lipid levels and CRC risk, we conducted dose-response meta-analyses using linear, quadratic, and restricted cubic spline (RCS) models. The best-fitting model was selected based on the lowest Akaike Information Criterion (AIC). Non-linearity in the spline models was assessed using the Wald test for the spline terms [26,27]. In studies reporting associations with low HDL levels, estimates were inverted to reflect high versus low comparisons for consistency.

All analyses were conducted using R software (version 4.4.1, 2024-06-14), utilizing the meta, metafor, and dosresmetapackages.

### Ethics approval and consent to participate

This study did not involve human participants, animals, or personal data; therefore, ethics approval and consent to participate were not applicable.

### Consent for publication

As no individual person’s data, images, or details are included in this study, consent for publication was not applicable.

## Results

### Study selection

**Fig 1:**
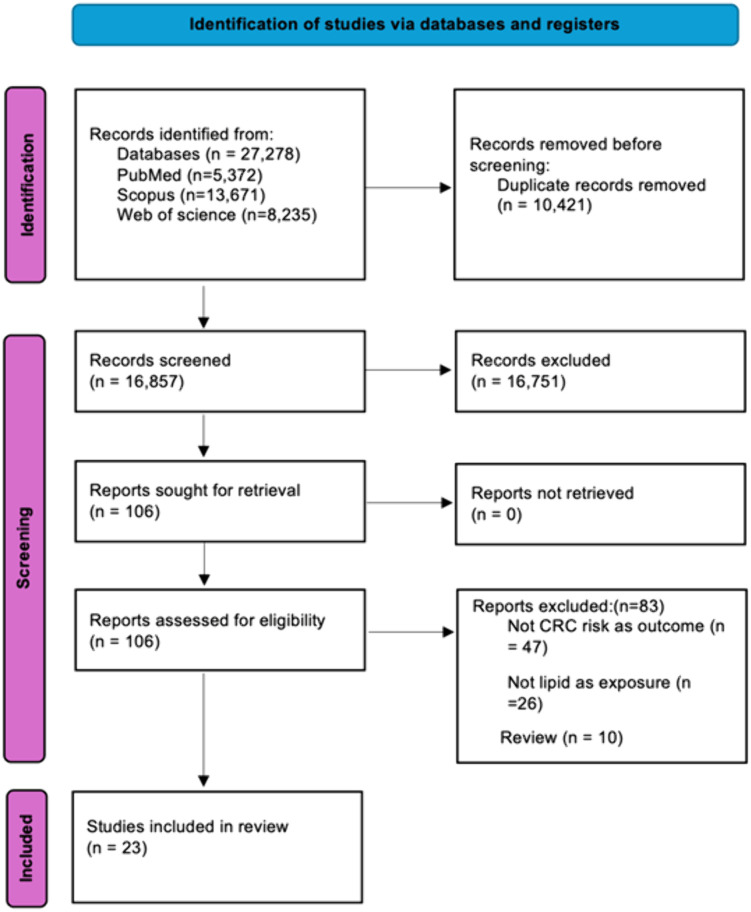
The PRISMA flow chart diagram.

As seen in Fig 1, the preliminary research revealed 27,278 articles, and then by removing duplicates, 16,857 studies remained. After screening for title and abstracts, 106 articles remained. Then, following the full-text screening of these studies (S15 Table in S3 File), 23 cohort studies were included, which met all of the inclusion criteria.

**Table 1:** Summary of the included studies.

Author(year)	Country	Type of study	Follow up duration(years)	Population	Sex (female%)	Type of cancer	Type of lipids	Adjustments	Out comes	Risk of bias assessment
Hsu et al. (2022) [2]	Taiwan	Cohort	16.7	4764	52.67%	CRC	LDL/HDL/TG/TSC	Age/gender/ DM/waist circumference/smoking history/ metabolic syndrome	↑ In men ↑ TSC/TG: CRC	9/11
Liu et al. (2022) [90]	Japan	Cohort	13	92770	20%	CRC	HDL/TG	age/sex/ educated/ income/marital status/BMI/TC/ALT/SUA/ alcohol/ smoking/tea/ salt/physical activity/high fat diet/family history	↑ TG/ ↓ HDL: ↑ CRC	9/11
Zhang et al. (2022) [91]	USA	Cohort	24	148291	67.5%	CRC/colon	TSC	Age/sex/race/ smoking/ physical activity/BMI/ alcohol/aspirin/ family history/history of DM/ multivitamin total caleries/red meat/fiber/folate/calcium/ vitamin D	Any association between TSC and CRC.	11/11
Fang et al. (2021) [92]	UK	Cohort	10.3	380087	52.81%	CRC	TC/LDL/HDL/TG	age/sex/race/ smoking /alcohol/ height/ family history/ diabetes/ Aspirin use/diet/BMI/ waist circumference/ physical activity	Any associate between TC/LDL/ HDL/TG and cancer	8/11
Li et al. (2019) [78]	China	Cohort	8.9	104333	0%	CRC	TG/HDL-C	age/education levels/income status/smoking/alcohol/sitting time	↑ WC: ↑ CRC The combination of WC/FBG: ↑ CRC	9/11
Katzeke et al. (2017) [93]	Germany	Cohort	15.6	25546	–	CRC	HDL/TG	age/sex/ smoking/ alcohol/height/ hypertension/ diabetes/diet/ BMI/waist/ physical activity/ red meat/fiber/ socioeconomic status/use of statin drugs	↑ HDL-C/apo(a) Lp(a): ↓ CRC mortality	9/11
Chandler et al.(2016) [79]	–	Cohort	19	15602	100%	CRC	TG/HDL/TSC	BMI/ menopausal status/physical activity/alcohol	↑ Apo A-1/HDL: ↓ CRC ↑ Apo B-100/TC: ↑ CRC	9/11
Muka et al. (2016) [41]	Netherland	Cohort	12.9	6628	–	CRC	TSC/ PUFAs	Age/sex/energy intake/dietary intake of PUFAs/red meat intake/smoking/physical activity/BMI/family history of disease	In low PUFAs situation: ↑ TSC ↑ CRC	11/11
Lu et al. (2015) [69]	Norway	Cohort	11.3	180553	–	CRC	HDL/TG	age/sex/ smoking/ alcohol/ physical activity/family history/BMI	↑ HDL/TG: ↑ CRC	10/11
Shin et al. (2014) [32]	Korea	Cohort	1	1326085	36%	CRC/ Colon/ rectum	cholesterol	age/BMI/serum cholesterol/ family history(men) age/height/ meat intake	high cholesterol: ↑ CRC	8/11
Strohmaier et al. (2013) [39]	Norway/Austria/Sweden	Cohort	11.7	577330	50%	Colon/ rectum	TSC	Age/BMI/ smoking	↓ High TCR: colon and rectum cancers	9/11
Kitahara et al.(2011) [60]	Korea	Cohort	14	1189719	36.4%	Colon/ rectum	TSC	Smoking/alcohol/BMI/ physical activity/ hypertension	High TSC: ↑ CRC	10/11
Iso et al.(2009) [52]	Japan	Cohort	14	33368	65%	Total cancer	TSC	Age	↑ TSC: ↑ total cancer	11/11
Borena et al.(2011) [72]	Norway/Austria/Sweden	Cohort	13.4(male)/ 11.9(female)	514097	50%	Total cancer	TG	Age/BMI/ smoking	↑ TG: ↑ total cancer	10/11
Ahn et al.(2009) [42]	southwestern Finland	Cohort	18	29133	100%	CRC	HDL TC	Age/ intervention/ education/ systolic BP/BMI/ physical activity/duration of smoking/alcohol/TSC/serum HDL	↑ TSC/HDL: ↓ CRC	10/11
Inoue et al.(2009) [30]	Japan	Cohort	10.2	27724	66.57%	Colon cancer	HDL-c/TC	BMI/Smoking/ alcohol	↑ TG: ↑ colon cancer in men.	10/11
Ahmed et al.(2006) [50]	USA	Cohort	11.5	14109	–	CRC	LDL/HDL	Age/sex/ family history of colorectal cancer/physical activity/ NSAID use/aspirin use/smoking/ alcohol	↑ HDL: ↑ CRC in men.	10/11
Tsushima et al.(2005) [68]	USA	Cohort	37	7619	0%	Colon/ rectal cancer	TG	Age/BMI/heart rate/smoking/ alcohol/total caloric intake	No associate between colon cancer and TG	10/11
Schoen et al.(1999) [49]	USA	Cohort	22.41	5849	–	CRC	LDL/HDL/TG	Age/sex/ physical activity	↑ LDL: ↑ CRC Any association between HDL and TG with CRC.	10/11
Gaard et al.(1997) [38]	Norway	Cohort	13	62173	55.29%	CRC/ rectum and colon cancer	HDL/LDL /TC	BMI/smoking/ age/ menopausal status	In women LDL/TC: Colon cancer. TSC: Rectum cancer. Any association between lipids and cancers in men.	6/11
Chyou et al.(1996) [29]	Hawaii	Cohort	21.9	7945	0%	Colon cancer/ rectal cancer	TSC	Age/BMI/TSC/heart rate/alcohol/ diet	↑ Serum cholesterol/ fatty acid intake: ↓ colon and rectal cancer.	8/11
Schatzkin et al.(1988) [55]	USA	Cohort	10	12488	59%	CRC	TSC	Age/Education /BMI/Smoking/ Alcohol/ Dietary fat intake/Dietary fiber intake/age at first birth/age at menarche parity/ menopause	In men ↑ TSC: ↓ CRC. Any association between TSC and CRC in women.	11/11
Tornberg et al.(1986) [61]	Sweden	Cohort	16	92000	50%	Colon cancer/ rectal cancer	Cholesterol/beta lipoprotein		↑ Cholesterol/beta lipoprotein: ↑ rectal/ rectum cancer ↑ dietary fat intake: ↑ CRC	10/11

(Abbreviations: CRC, Colorectal Cancer; WC, Waist Circumference; Mets, Metabolic Syndrome; HDL-C, High Density Lipoprotein Cholesterol; LDL, Low Density Lipoprotein Cholesterol; Lp(a), Lipoprotein(A); TC, Total Cholesterol; TSC, Total Serum Cholesterol; BMI, Body Mass Index; Mets, Metabolic Syndrome; NSAIDs, Nonsteroidal Anti-Inflammatory Drug; DM, Diabetes Mellitus; TG, Triglyceride; ALT, Alanine Transaminase; SUA, Single Umbilical Artery; FBG, Fasting Blood Glucose; PUFAs, Polyunsaturated Fatty Acid; BP, Blood Pressure)

### Study characteristics

All 23 studies included in this meta-analysis employed a cohort study design and were published between 1986 and 2024. Studies were conducted in different areas, including 8 studies in Asia: 3 in Japan [28–30], 2 in Korea [31,32], 2 in China [33,34], and 1 in Taiwan [35]. In total, 9 studies were conducted in Europe: 4 in Norway [36–39], 3 in Sweden [36,39,40], 2 in Austria [36,39], 1 in the Netherlands [41], 1 in Finland [42], 1 in Germany [43] and 1 in the UK [44]. A total of 6 studies were done in the USA [45–50]. In total, 4,694,828 participants were included. The studies identified 41,611 cases of CRC and 4,653,217 controls. There were 1,568,872 females (33%) and 3,111,847 males (67%) in studies, excluding one study that did not report the breakdown of females and males [50]. Among studies that reported age, the average age of the included participants was 51.1 years, with a range of 44 (29) to 73 (39) years. A total of 15 studies [28,29,31,32,35,38–47] studied the association between TC and the risk of CRC and 12 studies between TG and CRC risk [30,33–38,43,44,48–50], and 11 studies between high-density lipoprotein and CRC risk [30,33–35,37,38,42–44,49,50] and 3 studies between low-density lipoprotein levels and CRC risk [35,38,44]. Table 1 provides detailed information regarding the included articles. A summary of the fundings of the included studies can be found in S16 Table in S3 File. No funding biases were detected.

**Table 2:** Risk of bias assessment of cohort studies.

Article	1	2	3	4	5	6	7	8	9	10	11
Li et al. [33]	Yes	Yes	Yes	Yes	Yes	Yes	Yes	No	Yes	No	Yes
Katzke et al. [43]	Yes	Yes	Yes	Yes	Yes	Yes	Yes	No	Yes	No	Yes
Muka et al. [41]	Yes	Yes	Yes	Yes	Yes	Yes	Yes	Yes	Yes	Yes	Yes
Chandler et al. [47]	Yes	Yes	Yes	Yes	Yes	Yes	Yes	No	Yes	No	Yes
Lu et al. [37]	Yes	Yes	Yes	Yes	Yes	Yes	Yes	Yes	Yes	No	Yes
Shin et al. [32]	Yes	Yes	Yes	Yes	Yes	Yes	Yes	No	Yes	No	No
Strohmaier et al. [94]	Yes	Yes	Yes	Yes	Yes	Yes	Yes	No	Yes	No	Yes
Borena et al. [39]	Yes	Yes	Yes	Yes	Yes	Yes	Yes	Yes	Yes	No	Yes
Kitahara et al. [36]	Yes	Yes	Yes	Yes	Yes	Yes	Yes	Yes	Yes	No	Yes
Inoue et al. [31]	Yes	Yes	Yes	Yes	Yes	Yes	Yes	Yes	Yes	No	Yes
Iso et al. [30]	Yes	Yes	Yes	Yes	Yes	Yes	Yes	Yes	Yes	Yes	Yes
Ahn et al. [28]	Yes	Yes	Yes	Yes	Yes	Yes	Yes	Yes	Yes	No	Yes
Ahmed et al. [42]	Yes	Yes	Yes	Yes	Yes	Yes	Yes	Yes	Yes	No	Yes
Tsushima et al. [50]	Yes	Yes	Yes	Yes	Yes	Yes	Yes	Yes	Yes	No	Yes
Schoen et al. [49]	Yes	Yes	Yes	Yes	Yes	Yes	Yes	No	Yes	No	Yes
Gaard et al. [38]	Yes	No	Yes	Yes	Yes	No	No	Yes	Yes	No	No
Chyou et al. [29]	Yes	No	Yes	Yes	Yes	Yes	Yes	Yes	Yes	No	No
Schatzkin et al. [45]	Yes	Yes	Yes	Yes	Yes	Yes	Yes	Yes	Yes	Yes	Yes
Törnberg et al. [40]	Yes	Yes	Yes	Yes	Yes	Yes	Yes	Yes	Yes	No	Yes
Hsu et al. [35]	Yes	Yes	Yes	Yes	Yes	Yes	Yes	Yes	Yes	No	No
Liu et al. [34]	Yes	Yes	Yes	Yes	Yes	Yes	Yes	Yes	Yes	No	Yes
Zhang et al. [46]	Yes	Yes	Yes	Yes	Yes	Yes	Yes	Yes	Yes	Yes	Yes
Fang et al. [44]	Yes	Yes	Yes	Yes	Yes	No	Yes	Yes	Yes	No	No

1. Were the two groups similar and recruited from the same population?

2. Were the exposures measured similarly to assign people to both exposed and unexposed groups?

3. Was the exposure measured in a valid and reliable way?

4. Were confounding factors identified?

5. Were strategies to deal with confounding factors stated?

6. Were the groups/participants free of the outcome at the start of the study (or at the moment of exposure)?

7. Were the outcomes measured in a valid and reliable way?

8. Was the follow up time reported and sufficient to be long enough for outcomes to occur?

9. Was follow up complete, and if not, were the reasons to loss to follow up described and explored?

10. Were strategies to address incomplete follow up utilized?

11. Was appropriate statistical analysis used?

### Risk of bias assessment

All included studies had low risk of bias. A comprehensive evaluation of their quality is presented in Table 2. The majority scored 9/11 or higher, reflecting strengths in participant selection, exposure and outcome measurement, and confounding adjustment. Studies with lower scores, such as Gaard et al. (6/11), had limitations in reporting follow-up completeness or strategies to address incomplete follow-up.

## Meta-analysis and subgroup analysis

### Serum TC

#### Overall analysis.

**Fig 2:**
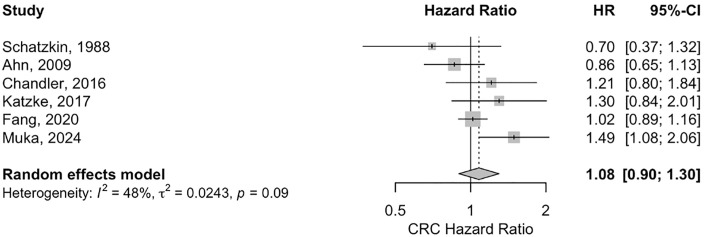
The forest plot for the association between high versus low serum cholesterol levels and the risk of colorectal cancer. HR, hazard ratio.

The relationship between serum Cholesterol and the risk of CRC was examined in 6 cohort studies, yielding an estimated log Hazard Ratio of 0.0763 (p = 0.42) using a Random-Effects Model. This corresponds to a pooled Hazard Ratio of 1.08 (95% CI: 0.90–1.30) with heterogeneity that may represent substantial heterogeneity (I^2^ = 50.55%, p = 0.09) (Fig 2). Similarly, Colon cancer was analyzed in 9 studies, producing a log Hazard Ratio of 0.0784 (p = 0.07) and a pooled Hazard Ratio of 1.08 (95% CI: 0.99–1.18) with heterogeneity that may represent moderate heterogeneity (I^2^ = 35.57%, p = 0.09). However, Rectal cancer examined in 7 studies, showed a significant log Hazard Ratio of 0.1767 (p = 0.000), translating to a pooled Hazard Ratio of 1.19 (95% CI: 1.08–1.32) with low heterogeneity (I^2^ = 28.36%, p = 0.40) (S1 and S2 Figs in S3 File).

Tests for funnel plot asymmetry indicated no evidence of publication bias for any cancer site, with p-values of 0.8983 for colorectal cancer, 0.7769 for colon cancer, and 0.4179 for rectal cancer (S43, S44, S45 Figs in S3 File).

#### Sensitivity analysis.

Sensitivity analyses were conducted to assess the robustness of overall estimates. For CRC, where the initial overall random effects model showed an HR of 1.08 (95%CI: 0.90–1.30) with heterogeneity of I^2^ = 50.55%, a sensitivity analysis was performed. This analysis yielded an HR of 1.01 (95%CI: 0.90–1.13), with significantly reduced heterogeneity of I^2^ = 13% (τ² < 0.0001, p = 0.33), indicating that the overall estimate for CRC was consistent (S3 Fig, S12 Table in S3 File).

#### Early exclusion.

Among studies of CRC in which no early exclusion of participants was applied, the pooled HR was 1.08 (95% CI: 0.90–1.30) with moderate heterogeneity (I^2^ = 48%, p = 0.09). For colon cancer, studies without early exclusion reported a pooled HR of 1.08 (95% CI: 0.99–1.18), whereas those with early exclusion yielded a slightly higher HR of 1.15 (95% CI: 0.79–1.68); the difference between subgroups was not statistically significant (p = 0.74). In rectal cancer, studies without early exclusion showed a pooled HR of 1.19 (95% CI: 1.08–1.32), while those with early exclusion reported 1.26 (95% CI: 0.68–2.33); again, the subgroup difference was not significant (p = 0.86). Overall, these results indicate that the application of early exclusion criteria did not influence the observed associations (S4, S5 and S6 Figs in S3 File).

#### Exclusion of extreme levels at baseline.

Subgroup analyses examining lipid status, specifically excluding extreme lipid levels at baseline, were performed for CRC, Colon cancer, and Rectal cancer. For CRC, studies that excluded extreme lipid levels showed a pooled HR of 1.02 (95% CI: 0.89–1.16), compared to an overall HR of 1.08 (95% CI: 0.90–1.30), with no significant subgroup difference observed (p = 0.59). In Colon cancer, exclusion of extreme lipid levels yielded an HR of 1.13 (95% CI: 1.03–1.24), slightly higher than the overall HR of 1.08 (95% CI: 0.99–1.18), though the subgroup difference was not statistically significant (p = 0.46). For Rectal cancer, studies excluding extreme lipid levels reported an HR of 1.08 (95% CI: 0.98–1.19), which was lower than the overall HR of 1.19 (95% CI: 1.08–1.32); notably, this difference was statistically significant (p = 0.02), indicating that lipid status may modify the association in rectal cancer (S7, S8 and S9 Figs in S3 File).

#### Study location.

Overall, the random effects models showed no significant regional differences in the association between risk and cancer type. For CRC, HRs were similar across regions, with Europe reporting 1.11 (95% CI: 0.88–1.35) and the USA 0.97 (95% CI: 0.57–1.64), and no significant subgroup difference (p = 0.66) (S10 Fig in S3 File). Colon cancer showed a significant association in European studies (HR 1.17; 95% CI: 1.01–1.35), while Asian (HR 1.08; 95% CI: 0.89–1.30) and USA studies (HR 0.98; 95% CI: 0.83–1.15) did not, with no significant regional difference overall (p = 0.29). For Rectal cancer, all regions showed elevated HRs, notably Europe (HR 1.29; 95% CI: 1.06–1.57), Asia (HR 1.18; 95% CI: 1.01–1.32), and USA (HR 1.28; 95% CI: 0.95–1.73), but subgroup differences by region were not statistically significant (p = 0.63). Heterogeneity varied among regions and cancer types but did not affect the overall lack of significant subgroup differences (S11 and S12 Figs in S3 File).

#### Risk of bias.

**Fig 3:**
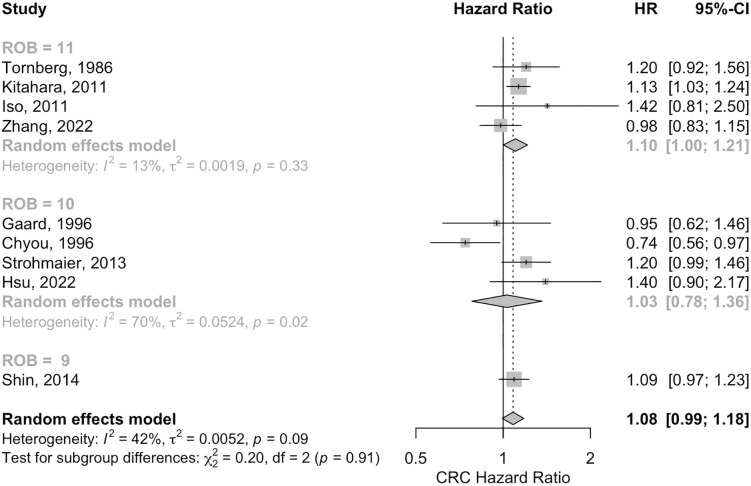
The forest plot for the risk of bias analysis of the association between high versus low serum cholesterol levels and colon cancer. HR, hazard ratio.

Subgroup analysis based on Risk of Bias (ROB) scores offered insights into the quality of studies examining cholesterol’s association with colorectal, colon, and rectal cancers. For CRC, the overall HR was 1.08 (95% CI: 0.90, 1.30). When stratified by ROB, studies with a score of 11 showed an HR of 1.01 (95% CI: 0.65, 1.56) with high heterogeneity (I^2^ = 75%, p = 0.02), those with ROB 9 had an HR of 1.04 (95% CI: 0.91, 1.18) with no heterogeneity, and ROB 10 studies had an HR of 1.30 (95% CI: 0.84, 2.01), with no significant differences across these groups (p = 0.61). For Colon cancer, the overall HR was 1.08 (95% CI: 0.99, 1.18), with ROB 11 studies reporting a significant HR of 1.16 (95% CI: 1.09, 1.24) and low heterogeneity (I^2^ = 13%), ROB 10 studies showing an HR of 1.03 (95% CI: 0.78, 1.36) with high heterogeneity (I^2^ = 70%), and ROB 9 studies having an HR of 1.09 (95% CI: 0.97, 1.23), again without significant subgroup differences (p = 0.91) (Fig 3). In Rectal cancer, the overall HR was 1.19 (95% CI: 1.08, 1.32), with ROB 11 studies yielding an HR of 1.19 (95% CI: 1.00, 1.41) and moderate heterogeneity (I^2^ = 20%), ROB 10 studies an HR of 1.21 (95% CI: 0.98, 1.53) with no heterogeneity, and ROB 9 studies showing an HR of 1.25 (95% CI: 1.12, 1.40); no significant differences were observed across ROB categories (p = 0.87) (S13 and S1 Figs in S3 File).

#### Cardiovascular disease/ diabetes mellitus exclusion.

Subgroup analyses concerning the exclusion of Cardiovascular Disease/Diabetes Mellitus (CVD_DM) patients at baseline revealed varying impacts across the three cancer types. For CRC, the overall random effects model yielded a HR of 1.08 (95% CI: 0.90, 1.30). Studies where CVD_DM patients were not excluded showed an HR of 1.13 (95% CI: 0.88, 1.45) with moderate heterogeneity (I^2^ = 57%, τ² = 0.0327, p = 0.07). Conversely, studies that did exclude CVD_DM patients had an HR of 0.98 (95% CI: 0.71, 1.36) with lower heterogeneity (I^2^ = 44%, τ² = 0.0258, p = 0.18); the subgroup difference for CRC was not statistically significant (χ² = 0.46, df = 1, p = 0.50). For Colon cancer, the overall random effects model indicated an HR of 1.08 (95% CI: 0.99, 1.18). Studies without CVD_DM exclusion had an HR of 1.05 (95% CI: 0.93, 1.19) (I^2^ = 49%, τ² = 0.0131, p = 0.06), while those with exclusion showed an HR of 1.14 (95% CI: 1.04, 1.25) with no heterogeneity (I^2^ = 0%, τ² = 0, p = 0.43); no significant subgroup difference was observed for colon cancer (χ² = 0.91, df = 1, p = 0.34). Conversely, for Rectal cancer, the overall random effects model presented an HR of 1.19 (95% CI: 1.08, 1.32). In this case, studies where CVD_DM patients were *not* excluded reported an HR of 1.28 (95% CI: 1.15, 1.35) with low heterogeneity (I^2^ = 3%, τ² = 0.0043, p = 0.40), whereas those that *did* exclude CVD_DM patients showed an HR of 1.08 (95% CI: 0.98, 1.19) with no heterogeneity (I^2^ = 0%, τ² = 0, p = 0.98); a statistically significant subgroup difference was noted for rectal cancer (χ² = 5.15, df = 1, p = 0.02) (S15, S16 and S17 Figs in S3 File).

#### Meta-regression analysis.

We performed meta-regression analyses using sex, age, follow-up duration, and body mass index (BMI) as moderators. The results indicated that none of the variables significantly influenced the effect estimates. The hazard ratio We conducted meta-regression analyses to assess the influence of sex, age, follow-up duration, and body mass index (BMI) as moderators on the association between cholesterol and cancer risk. For CRC, none of the moderators significantly affected the effect estimates, with HR of 0.67 (95% CI: 0.39–1.16, p = 0.15) for sex, 1.02 (95% CI: 0.99–1.05, p = 0.15) for age, 1.00 (95% CI: 0.94–1.05, p = 0.87) for follow-up duration, and 0.89 (95% CI: 0.61–1.30, p = 0.54) for BMI. Similarly, for Colon cancer, sex (HR = 1.23, 95% CI: 0.73–2.07, p = 0.44) and follow-up duration (HR = 0.99, 95% CI: 0.98–1.01, p = 0.35) showed no significant moderation. In Rectal cancer, sex (HR = 0.69, 95% CI: 0.27–1.80, p = 0.45) and follow-up duration (HR = 1.00, 95% CI: 0.98–1.02, p = 0.95) also did not significantly influence the results. Heterogeneity across these models was generally low to moderate. HR associated with sex was 0.93 (95% CI: 0.82–1.07, p = 0.32), showing no significant difference between sexes. Similarly, age (HR = 0.99, 95% CI = 0.99–1.01, p = 0.82), follow-up duration (HR = 0.99, 95% CI = 0.98–1.00, p = 0.25), and BMI (HR = 0.997, 95% CI: 0.96–1.04, p = 0.88) were not statistically significant. Heterogeneity varied across models, ranging from 0% for age to 15% for BMI (S6, S7, S8 Tables in S3 File).

### Serum TG

#### Overall analysis.

**Fig 4:**
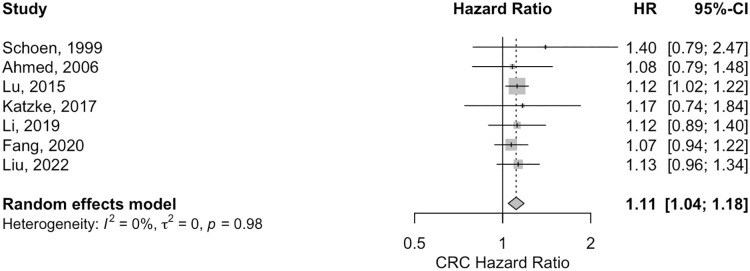
The forest plot for the association between high versus low serum TG levels and the risk of colorectal cancer. HR, hazards ratio.

The results regarding Triglycerides for the three cancer types analyzed show varied associations. For CRC, the meta-analysis of 7 studies revealed a pooled HR of 1.11 with a 95% Confidence Interval (CI) of 1.044–1.18, indicating a statistically significant positive association (p-value = 0.0008). This finding was supported by no significant heterogeneity across studies (I^2 = 0.00%, p-value = 0.9812) (Fig 4) and no evidence of funnel plot asymmetry (z = 0.4830, p = 0.6291) (S46 Fig in S3 File). In the case of Colon cancer, based on 5 studies, the pooled HR for TG was 1.23(95% CI: 0.99–1.55) (S18 Fig in S3 File), suggesting a marginally significant association (p-value = 0.0576). Moderate heterogeneity was observed (I^2 = 52.01%, p-value = 0.0763), but the funnel plot asymmetry test showed no significant bias (z = 0.0882, p = 0.9297) (S47 Fig in S3 File). Lastly, for Rectal cancer, data from 4 studies indicated a pooled HR of 1.036 (95% CI: 0.69–1.56), which was not statistically significant (p-value = 0.8674) (S19 Fig in S3 File). This analysis exhibited substantial heterogeneity (I^2 = 67.29%, p-value = 0.0327) and no significant funnel plot asymmetry (z = −0.5281, p = 0.5974) (S48 Fig in S3 File).

#### Sensitivity analysis.

The sensitivity analyses for triglycerides (TG) were conducted separately for colon and rectum cancer outcomes. For colon cancer, the sensitivity analysis yielded a pooled Hazard Ratio of 1.37(95% CI: 1.17–1.61). The heterogeneity for this analysis was I^2^ = 17%, τ² < 0.0001, and p = 0.31. This outcome differs from the overall pooled HR for colon TG, which was 1.24 (95% CI: 0.99–1.55).For rectum cancer, the sensitivity analysis showed a pooled HR of 1.28(95% CI: 1.01–1.61). The heterogeneity in this case was I^2^ = 0%, τ² = 0, and p = 0.99. This result also contrasts with the overall pooled HR for rectum TG, which was 1.04 (95%CI: 0.69–1.56). Leave-one-out sensitivity analysis indicated that exclusion of Study 2 increased the pooled HR to 1.37 (95% CI: 1.17–1.61) and eliminated heterogeneity, suggesting the colon result is sensitive to that single study (S13 Table in S3 File).

For TG and rectal cancer, excluding Study 3 produced a pooled HR of 1.28 (95% CI: 1.01–1.61), again indicating sensitivity to an individual study (S14 Table in S3 File) (S20 and S21 Figs in S3 File).

#### Early exclusion.

The forest plots examining the effect of early exclusion on TG across the three cancer types reveal varied results. For CRC, the plot shows a pooled HR of 1.11 (95% CI: 1.04; 1.18) from studies without early exclusion, with no heterogeneity observed (I^2^ = 0%, p = 0.98). In Colon cancer, two subgroups are presented: studies with early exclusion had a pooled HR of 1.26 (95% CI: 0.72; 2.21) accompanied by substantial heterogeneity (I^2^ = 63%, p = 0.10), while those without early exclusion had a pooled HR of 1.24 (95% CI: 0.94; 1.63) with moderate heterogeneity (I^2^ = 53%, p = 0.06). The combined pooled HR for Colon cancer was 1.24 (95% CI: 0.99; 1.55), with no significant difference between subgroups (p = 0.95). For Rectal cancer, the subgroup with early exclusion showed a pooled HR of 1.22 (95% CI: 0.70; 2.14) and high heterogeneity (I^2^ = 77%, p = 0.01), whereas the subgroup without early exclusion had a pooled HR of 1.04 (95% CI: 0.69; 1.56) with substantial heterogeneity (I^2^ = 66%, p = 0.03). The overall pooled HR for Rectum cancer was 1.04 (95% CI: 0.69; 1.56), and the subgroup difference test was not statistically significant (p = 0.58) (S22 and S23 Figs in S3 File).

#### Exclusion of extreme levels at baseline.

The analysis of excluding extreme lipid levels at baseline provided insights primarily for CRC and Colon cancer, though data for Rectum cancer under this criterion were not sufficient. For CRC, the pooled Hazard Ratio for TG when excluding extreme lipid levels was 1.07 (95% CI: 0.94–1.22), compared to an overall HR of 1.11 (95% CI: 1.04–1.18) across all lipid statuses. There was no significant subgroup difference by lipid status (p = 0.51), and heterogeneity was absent (I^2^ = 0%, p = 0.98) in both groups. For Colon cancer, the overall pooled HR was 1.24 (95% CI: 0.99–1.55) with moderate heterogeneity (I^2^ = 53%, p = 0.08). Data on Rectal cancer on this subject were insufficient for analysis (S24 and S25 Figs in S3 File).

#### Study location.

The analysis of TG by geographical region across the three cancer types showed varying subgroup results but no statistically significant differences between regions. For CRC, the pooled Hazard Ratios were 1.15 (95% CI: 0.87–1.51) for the USA and 1.11 (95% CI: 1.03–1.19) for Europe, both with no heterogeneity. The overall HR for CRC was 1.11 (95% CI: 1.04–1.18), with no significant subgroup difference by region (p = 0.95), indicating a consistent association regardless of location. In Colon cancer, regional HRs varied more, with Europe at 1.26 (95% CI: 0.83–1.93) showing considerable heterogeneity, the USA at 1.43 (95% CI: 1.09–1.88), and Asia at 0.92 (95% CI: 0.67–1.26). Despite this variability, the overall HR was 1.24 (95% CI: 0.99–1.55), and subgroup differences by region were not statistically significant (p = 0.11) (S26 and S27 Figs in S3 File).

#### Risk of bias.

The analysis of Risk of Bias scores for TG across colorectal, colon, and rectum cancers showed that pooled Hazard Ratios were generally consistent regardless of ROB level, with no significant differences between ROB subgroups. For CRC, studies with ROB scores of 9, 10, and 11 reported similar HRs ranging from 1.08 to 1.13, all with no heterogeneity, and no significant subgroup differences were found (p = 0.90). In Colon cancer, the ROB = 10 subgroup showed considerable heterogeneity with an HR of 1.26, while the ROB = 11 subgroup had an HR of 1.24 with moderate heterogeneity; however, subgroup differences were not significant (p = 0.95). For Rectum cancer, both ROB = 10 and ROB = 11 subgroups demonstrated high heterogeneity and HRs of 1.22 and 1.04, respectively, but again, no significant differences were detected between these groups (p = 0.58). Overall, the results suggest that study quality, as measured by ROB scores, did not significantly influence the association between TG and cancer risk (S28, S29 and S30 Figs in S3 File).

#### Cardiovascular disease/ diabetes mellitus exclusion.

The analysis of baseline exclusion of Cardiovascular Disease and Diabetes Mellitus showed differing effects across the three cancer types for TG. For CRC, studies that did not exclude patients with CVD/DM had a pooled Hazard Ratio of 1.11 (95% CI: 1.04–1.19) with no heterogeneity, while studies that excluded these patients showed a similar HR of 1.12 (95% CI: 0.89–1.40). The overall HR for CRC was 1.11 (95% CI: 1.04–1.18), and subgroup differences by CVD/DM exclusion were not statistically significant (p = 0.95), indicating this factor did not meaningfully affect the association. For Colon cancer, only data for studies without CVD/DM exclusion were available, showing a pooled HR of 1.24 (95% CI: 0.99–1.55) with moderate heterogeneity (S31 and S32 Figs in S3 File).

#### Meta-regression analysis.

Meta-regression analyses of TG across colorectal, colon, and rectum cancers examined potential moderating effects of sex, age, and follow-up duration, finding no statistically significant influences on the pooled Hazard Ratios. For CRC, sex (HR = 1.03, p = 0.87), age (HR = 1.00, p = 0.90), and follow-up duration (HR = 0.99, p = 0.78) showed no significant moderation, with zero heterogeneity. In Colon cancer, sex approached marginal significance (HR = 0.51, p = 0.06) but follow-up duration did not (HR = 0.98, p = 0.62), with moderate heterogeneity for follow-up. For Rectum cancer, neither sex (HR = 0.69, p = 0.45) nor follow-up duration (HR = 1.00, p = 0.95) significantly moderated the association. Overall, these results indicate that demographic and study-level factors did not substantially affect the relationship between TG and cancer risk, supporting the robustness of the primary findings (S9, S10 Tables in S3 File).

### Serum HDL

#### Overall analysis.

**Fig 5:**
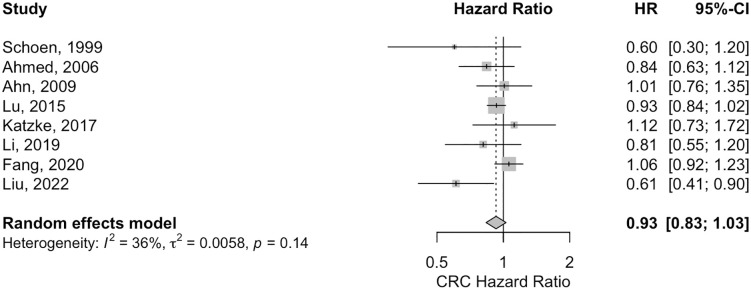
The forest plot for the association between high versus low serum HDL levels and the risk of colorectal cancer. HR, hazards ratio.

The relationship between serum HDL and the risk of CRC was assessed by 8 studies, while 3 studies focused on colon cancer and 2 studies on rectum cancer, all exploring the association between serum HDL and the risk of these cancers. The analysis indicated no statistically significant association between higher levels of HDL (HR 0.93; 95% CI: 0.83; 1.03) and CRC risk (Fig 5). Similarly, the results for both colon cancer (HR 0.94; 95% CI: 0.75; 1.19) and rectum cancer (HR: 0.95; 95% CI: 0.66;1.37) were also not statistically significant (S33 and S34 Figs in S3 File).

Studies on HDL showed moderate heterogeneity for CRC (I^2^ = 36%, p = 0.14), and no heterogeneity for Colon cancer (I^2^ = 0.00%, p = 0.78) and Rectum cancer (I^2^ = 0.00%, p = 0.37), respectively. No significant result was shown by the funnel plot asymmetry for HDL in, CRC (z = −1.5017, p = 0.1332) and for colon cancer (z = 0.4552, p = 0.6490) (S49, S50 and S51 Figs in S3 File).

#### Exclusion of extreme levels at baseline.

The analysis investigating the impact of excluding extreme lipid levels at baseline included data for CRC, Colon, and Rectum cancers. For CRC, the pooled Hazard Ratio for HDL was 0.93 (95% CI: 0.83;1.03, p = 0.14) with minimal heterogeneity (I^2^ = 0.36%). For Colon cancer, the overall pooled HR for HDL was 0.94 (95% CI: 0.75;1.19, p = 0.78) with no heterogeneity (I^2^ = 0.00%). For Rectum cancer, the pooled HR was 0.95 (95% CI: 0.66–1.37, p = 0.37) with no heterogeneity (I^2^ = 0.00%) (S35 Fig in S3 File).

#### Study location.

For CRC, the meta-analysis by geographical region included studies from the USA, Europe, and Asia. The pooled Hazard Ratios were 0.80 (95% CI: 0.61;1.04, p = 0.38) for the USA, 0.99 (95% CI: 0.89;1.09, p = 0.45) for Europe, and 0.70 (95% CI: 0.53;0.93, p = 0.32) for Asia, with no heterogeneity observed within any subgroup (I^2^ = 0.00%). However, the overall heterogeneity across all studies was moderate, with an I^2^ value of 36%, indicating some variability when combining results from the three regions. Similar to the non-significant p-values observed in each subgroup, the p-value for the comparison among the three regions was also not significant (p = 0.14; HR = 0.93; 95% CI: 0.83–1.03) (S36 Fig in S3 File).

#### Risk of bias.

For CRC, studies with ROB scores of 9, 10, and 11 reported pooled HR of 0.89, 0.81, and 0.93 respectively with differing heterogeneity levels, high heterogeneity for ROB 9, moderate heterogeneity for ROB 10, and no heterogeneity for ROB 11. Also, no significant differences were observed between subgroups (p = 0.14). Overall, the findings indicate that the quality of studies, assessed through ROB scores, had no significant impact on the relationship between HDL and CRC risk. For Rectum cancer and CRC, the available data were not sufficient for an analysis (S37 Fig in S3 File).

#### Cardiovascular disease/ diabetes mellitus exclusion.

The analysis grouped by cardiovascular disease/Diabetes Mellitus (CVD_DM) exclusion status, indicate that HDL lipid status demonstrates no statistically significant association with CRC. For CRC, (HR = 0.93; 95% CI = 0.83–1.03). This finding shows moderate heterogeneity (I^2^ = 36%, τ^2 ^= 0.0058), and the test for subgroup differences was not significant (p = 0.14). For Rectum cancer and CRC, data were insufficient to perform a comprehensive analysis (S38 Fig in S3 File).

#### Meta-regression analysis.

In meta-regression analysis for HDL in CRC, neither Sex nor Follow-up duration significantly moderated the results. Specifically, for CRC, sex showed no significant effect (HR = 0.79, p = 0.43), age had no significant influence (HR = 0.99, p = 0.76), and follow-up duration also did not significantly affect the results (HR = 1.01, p = 0.57). Data on Rectal and Colon cancers on this subject were insufficient for analysis. In summary, demographic and study-level variables showed no significant impact on the relationship between HDL and CRC risk, reinforcing the reliability of the main results (S11 Table in S3 File).

### Serum LDL

#### Overall analysis.

For Colon cancer, the pooled Hazard Ratio for LDL was 0.91 (95% CI: 0.60; 1.37, p = 0.21), indicating no statistically significant association between LDL levels and colon cancer risk. Moderate heterogeneity was observed among the studies (I^2^ = 37%) (S39 Fig in S3 File). Funnel plot analysis showed no evidence of publication bias (S52 Fig in S3 File). Also, for Rectum cancer and CRC, data were insufficient to perform a comprehensive analysis.

## Dose-response analyses

### Total cholesterol

**Fig 6:**
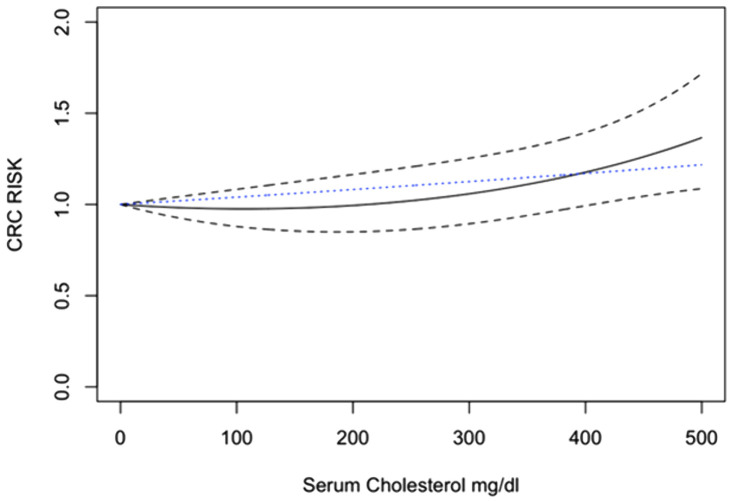
Dose-response analysis of the association between serum cholesterol levels and the risk of colorectal cancer: Quadratic and Linear Models.

By applying linear, quadratic, and RCS models, the quadratic model represented the best fit with the lowest AIC (χ² = 7.28, p = 0.026) and indicated an association that was statistically significant between increasing TC levels and CRC risk (Fig 6). The dose-response analysis indicated that increased TC levels were significantly linked to a higher risk of CRC. S2 Table in S3 File demonstrates the specific dose levels and their odds ratios.

### TG

Similarly, the TG quadratic model provided a better fit than the linear model and indicated a statistically significant relationship between increased TG levels and the risk of CRC (χ² = 10.88, p = 0.004) (S40 Fig in S3 File). Moderate heterogeneity (I^2^ = 29.7%) was observed in the dose-response relationship among studies assessing TG serum levels. The specific dose levels and their odds ratios are shown in S3 Table in S3 File.

### HDL and LDL

The quadratic model offered the best fit for both LDL and HDL with the lowest AIC in comparison with other models, however, this model did not report significant associations with CRC risk and higher levels of LDL (χ² = 0.12, p = 0.942) or HDL (χ² = 2.44, p = 0.295) (S41 and S42 Figs in S3 File). Heterogeneity was not observed for HDL (I^2^ = 0.0%) but indicated moderate for LDL (I^2^ = 31.7%). The specific dose levels and their odds ratios are shown in S4 and S5 Tables in S3 File.

## Discussion

In this study, we examined the associations between high TC, high TG, high LDL, and HDL levels and the hazard of CRC, colon and rectum cancers, finding different associations between higher TG, TC, HDL and LDL levels and a hazard of these cancers. The results of this study showed that high TG level was associated with an increased risk of CRC, a marginally significant association was reported for colon cancer and no significant association was observed for rectum cancer. In contrast, higher TC levels were not associated with the risk of CRC or colon cancer, but were associated with an increased risk of rectum cancer. Additionally, higher HDL level was not associated with the risk of any of the three cancers mentioned. This was while higher LDL level was not associated with the risk of colon cancer.

According to the results of this study,higher levels of TC were not significantly associated with the risk of CRC (HR = 1.08; 95% CI: 0.90–1.30; I^2^ = 50.55%; p = 0.865) and colon cancer (HR = 1.08; 95% CI: 0.99–1.18; I^2^ = 35.57%; p = 0.0877), but were significantly associated with an increased risk of rectum cancer (HR = 1.19; 95% CI: 1.08–1.32; I^2^ = 28.36%; p = 0.0004). Cholesterol is associated with cancer development and may participate in several key oncogenic signaling pathways. It can directly activate the Hedgehog and Wnt/β-catenin pathways, which are crucial for cell proliferation and have been linked to cancers such as colorectal and pancreatic. Additionally, cholesterol contributes to activating the mTORC1 pathway, which governs cell growth and survival. Its presence in lipid rafts further supports signaling through pathways like EGFR, HER2, and PI3K/AKT, which are pivotal for the progression of tumors. Furthermore, cholesterol influences immune-related signaling via the JAK/STAT pathway, especially interferon-gamma (IFN-γ) signaling, which impacts tumor immunogenicity and escape mechanisms [51].

However, in contrast to our results, some articles demonstrated either no association or the inverse relationship between cholesterol and CRC risk [39,52]. Ahn et al. [53] revealed that decreased blood cholesterol levels could represent a sign of malignancies. According to their research, higher cholesterol levels were linked to a lower incidence of cancer compared to individuals with lower cholesterol levels. This could be due to their population of study which included only male cigarette smokers. It might also be the consequence of the metabolic impacts of preclinical, undetected tumors, which may lower cholesterol levels [54]. Additionally, it’s possible that those with higher serum cholesterol levels had a higher chance of dying from cardiovascular causes before receiving a cancer diagnosis [55]. Chyou et al [56] revealed a negative association between cholesterol and CC, but no association with RC. This could be attributed to different microbiota inhabiting different regions of the intestine that play multiple roles on tumor progression [57]. Nevertheless, further research is needed to explore the interplay between gut microbiota, lipid metabolism, and CRC risk. Recent advances in bacterial-based delivery systems, such as bacterial ghosts and extracellular vesicles, highlight the potential of leveraging microbiota-derived nanoparticles for targeted cancer therapies, which could also inform novel strategies for modulating lipid-related pathways in CRC prevention and treatment [58].

Also, Zhe Fang et al. [44] conducted a prospective cohort study in 2020 involving 380,087 adults aged 40–69 years using data from the UK Biobank. According to these findings, after adjusting for potential confounders, such as BMI and waist circumference none of the lipid biomarkers (HDL, LDL, TC, TG, ApoA, ApoB) showed a significant association with CRC risk. The discrepancies observed between the findings of this study and our results may be attributable to several factors. These include differences in the age structure of the study population, as a significant proportion of participants entered the study after the age of 50, thereby limiting the statistical power to investigate early-onset colorectal cancer. Another factor may be the potential influence of early metabolic alterations in lipid profiles, which could be a consequence of carcinogenic processes and thus confound the association between lipids and cancer risk. Furthermore, the analysis was limited to circulating lipids, without consideration of local biochemical changes within the gut microenvironment, which may play a more significant role in carcinogenesis. Lastly, differences in study design, follow-up duration, and measurement methods may also have contributed to the observed inconsistencies. Another study conducted in by Ronac Mamtani et al. [59] also investigated this issue. This study was a nested case–control study that investigated the association between statins, cholesterols and CRC. The results of this study indicated that, independent of statin use, there is an inverse association between serum cholesterol levels and the risk of colorectal cancer. In other words, the findings of this study demonstrated that decreased levels of LDL, HDL, and TG are significantly associated with an increased risk of CRC. The association observed between serum lipid reduction and short-term increased colorectal cancer risk, regardless of statin use, may reflect the presence of undiagnosed malignancy in the early stages. The difference in findings compared to our study may be attributed to differences in follow-up duration.

There have also been many articles in agreement with our study [60–62]. Törnberg et al. [61] reported a positive link between high serum cholesterol levels and risk of RC in men, based on a study involving 92,000 subjects. Yang et al. [62] performed a systematic review which resulted in the same positive association. Hypercholesterolemia is one of the factors that can activate carcinogenic signaling pathways [63]. As previously reported, cholesterol may be a significant factor in acute phase reactions during a number of diseases [64]. Since it participates in cell proliferation as well, it may have an effect on aberrant tumor growth [65]. Zhang et al. [66] discovered that CRC metastasized as a result of a cholesterol metabolic pathway triggered by Sterol Regulatory Element-Binding Protein 2 (SREBP2). Huang et al. [67] revealed that high cholesterol in colorectal cancer cells activated the ATP6V0A1 pathway, which in turn causes CD8 + T cells to become inactive and aids in tumor immune evasion. These explained mechanisms could collectively account for the role of cholesterol.

According to the results of this study, higher serum TG level was associated with a 11% higher hazard of CRC (HR 1.11; 95% CI: 1.044–1.18,), with low heterogeneity (I^2^ = 0.00%). Also, for colon cancer, TG demonstrated a marginally significant relationship (HR 1.23; 95% CI: 0.99–1.55; I^2^ = 52.0%; p = 0.0576). No significant link was observed between TG levels and the risk of rectum cancer (HR 1.036; 95% CI: 0.69–1.56; I^2^ = 67.29%; p = 0.8674).

However, Tsushima et al [68] discovered no meaningful associations between TG levels and CRC. Lu Y et al. [69] found that elevated TG levels were linked to a higher risk of proximal colon adenocarcinoma in men and rectal adenocarcinoma in women. A reason behind the different results could be the fluctuations of TG serum levels after meal consumptions [70]. In various other studies TG levels and men’s risk of colon cancer have been positively correlated [71–73]. Fang et al. [44] published a cohort study on 390,000 subjects which showed that elevated TG levels were linked to a higher risk of CRC, especially in the caecum and transverse colon. Additionally, Yang et al. [62] conducted a systematic review in which the same positive link was observed. Triglycerides are needed for the rapid metabolism of cancer cells and therefore higher levels of TG can easily provide for the high energy demand. Fatty acids are the primary energy source of tumor growth [74]. On the other hand, hypertriglyceridemia has been hypothesized to be linked to the production of reactive oxygen species and the formation of oxidative stress, which are elevated in cancer cells and play an important role in normal cell proliferation [75]. In the intestine, triglycerides are linked to fecal bile acids, which have been shown to have a close relationship with colorectal cancer [76]. Yarla et al. [77] reported that dysregulation of TG caused overactivation of TG anabolic pathways and inactivation of TG catabolic pathways, both of which are associated with CRC tumor growth and metastasis.

However, this analysis showed elevated HDL levels were not significantly related to CRC risk (HR 0.93; 95% CI: 0.83–1.03; I^2^ = 28.8%; p = 0.1433), colon cancer (HR 0.94; 95% CI: 0.75–1.19; I^2^ = 0.0%; p = 0.6490), or rectum cancer (HR 0.95; 95% CI: 0.66–1.37; I^2^ = 0.0%; p = 0.37),and colon cancer, higher LDL level was not significantly associated with risk (HR 0.91; 95% CI: 0.60–1.37; p = 0.21; I^2^ = 37%).

However, Yang et al. (14) found a substantial relationship between a high HDL level and a lower risk of colorectal cancer, which is in contradiction to our study. In one case control study on CRC cases, the same results were obtained. There were notable associations between high ApoA and HDL and risk reduction of CRC [22]. However, there are articles that report inverse or no association of HDL with CRC [71,78,79]. The contrasting results on HDL could be due to imbalance of lipidomic in various diseases like metabolic syndrome [80]. These lipid profiles and cancer risk have a profoundly complicated relationship. While HDL and LDL are usually known as cardiovascular markers, their mechanisms in cancer progression are still unclear. HDL acts as a protective agent against the oxidation of LDL by inhibiting it. Therefore, HDL prevents tumor growth by its antioxidant property and also by regulating the cholesterol level of cell membranes which influences signaling pathways that could potentially lead to carcinogenic effects [81–83]. The anti-inflammatory characteristics of HDL might get entirely blocked out by other pathways and mechanisms involved in CRC [84]. Recent research increasingly suggests that different subtypes of HDL and LDL could exhibit distinct biological effects. Our review’s inability to differentiate between these subtypes could possibly explain the significant lack of associations with CRC risk [85].

Our analysis showed a significant association between higher TG levels and the risk of CRC, whereas in colon and rectum cancer, higher TG level was not significantly associated with the risk.

The proximal and distal colons serve various purposes because of their different embryonic origins. They also contain different compositions of bile acids [86]. The rate of occurrence for proximal colon is high among females and in the distal colon and rectum is higher among men and it is influenced by the distinct levels of sex hormones between men and women [69]. According to Fang et al., elevated TG levels raised the risk of transverse colon and caecum cancer. [44].

The are studies in which a certain lipid is only considered a CRC risk factor in one sex but not the other [69,71,87]. This could be due to the hormonal factors that are specific for females which limits our understanding of cancer risk factors that could be influenced by various hormones [88]. It has been proposed that the ways in which progesterone and estrogen protect the colon are one of the reasons that high serum TG is not regarded as a risk factor for CC in women [89]. In the dose-response analysis, we selected the model based on the lowest AIC value to identify the best fit. In some cases, the quadratic model provided a better fit than the linear model, which may indicate that the association between lipid levels and colorectal cancer follows a non-linear pattern. These findings highlight the importance of considering non-linearity in exposure–risk relationships when interpreting dose-response trends. We examined whether the quality of the included studies (risk of bias) influenced the results of our meta-analysis. In some subgroups, such as those with higher-quality studies, the association between certain lipid levels (e.g., cholesterol and triglycerides) and CRC risk was statistically significant. However, the overall comparisons between different ROB levels did not show major differences, and the tests for subgroup differences were not significant. This suggests that while study quality may have contributed to some variation, it did not have a substantial impact on the overall findings. However, the presence of bias in some studies, such as confounding or selection bias, may still affect the reliability of the findings. Therefore, our results should be interpreted with caution, considering the observational design and varying study quality.

This study had some limitation. The impacts of some factors have not been thoroughly considered in our included articles. The hormonal state of the cases was not stated and as previously mentioned the female hormones have a notably great impact on how various lipids effect different parts of the intestine. Therefore, lack of data on hormones could have potentially caused different incidence rates among men and women and create heterogeneity. In addition, as previously stated, microbiota is also a key factor that could impact tumor progression since these microbes can produce multiple metabolites that play different role in inflammation and carcinogenesis. And these microorganisms have not been considered in any of our included articles. One other limitation could be the variability in the adjustments across our included articles. For example, only some articles considered lifestyle factors like smoking and alcohol intake as an adjustment. Few articles adjusted for education levels, whereas some focused solely on age. Factors like menopausal state, physical activity and diet were also adjustments considered in some of the included articles. These differences could affect the comparability and generalization of our results.

In conclusion, elevated levels of TG were associated with a higher risk of CRC. Our research clearly demonstrated no connection between elevated HDL, LDL or TC levels and a higher risk of CRC, colon and rectum cancers. Further research is desperately required to identify the precise risk factors and their processes for CRC, as several crucial components, such as hormones and microbiome, have not been considered in many studies. The findings of this meta-analysis, highlighting a clear link between high levels TG and higher risk of CRC, have important implications for public health and nutrition strategies. These results suggest that dyslipidemia may be linked to colorectal cancer risk, though further research is needed to clarify its role. Thus, public health efforts that encourage lower lipid levels, such as decreasing saturated fat consumption, increasing dietary fiber, engaging in regular physical activity, and making general lifestyle changes, could not only help prevent cardiovascular disease but also possibly lower CRC rates. Incorporating cholesterol-lowering strategies into national dietary guidelines and cancer prevention programs could provide a dual advantage for both metabolic and cancer-related health.

## Supporting information

S1 File PRISMA 2020 checklist. (DOCX)

S2 File Data extraction of the included studies. (XLSX)

S3 File Additional figures, tables, and appendices. (DOCX)

## References

[1] Morgan E, Arnold M, Gini A, Lorenzoni V, Cabasag CJ, Laversanne M, et al. Global burden of colorectal cancer in 2020 and 2040: incidence and mortality estimates from GLOBOCAN. Gut. 2023;72(2):338–44. doi: 10.1136/gutjnl-2022-327736 36604116

[2] Hsu S-H, Syu D-K, Chen Y-C, Liu C-K, Sun C-A, Chen M. The Association between Hypertriglyceridemia and Colorectal Cancer: A Long-Term Community Cohort Study in Taiwan. Int J Environ Res Public Health. 2022;19(13):7804. doi: 10.3390/ijerph19137804 35805464 PMC9265720

[3] Li Q, Yu M, Lv H, Zhang L, Deng Y, Yu H. Burden of early-onset colorectal cancer along with attributable risk factors from 1990 to 2019: a comparative study between China and other G20 countries. BMC Public Health. 2023;23(1):1463. doi: 10.1186/s12889-023-16407-y 37525147 PMC10391986

[4] GBD 2019 Colorectal Cancer Collaborators. Global, regional, and national burden of colorectal cancer and its risk factors, 1990-2019: a systematic analysis for the Global Burden of Disease Study 2019. Lancet Gastroenterol Hepatol. 2022;7(7):627–47. doi: 10.1016/S2468-1253(22)00044-9 35397795 PMC9192760

[5] Su X, Chen X, Wang B. Pathology of metabolically-related dyslipidemia. Clin Chim Acta. 2021;521:107–15. doi: 10.1016/j.cca.2021.06.029 34192528

[6] Huang J, Lin H, Wang S, Li M, Wang T, Zhao Z, et al. Association between serum LDL-C concentrations and risk of diabetes: A prospective cohort study. J Diabetes. 2023;15(10):881–9. doi: 10.1111/1753-0407.13440 37461165 PMC10590678

[7] Du Z, Qin Y. Dyslipidemia and cardiovascular disease: current knowledge, existing challenges, and new opportunities for management strategies. J Clin Med. 2023;12(1). 10.3390/jcm12010363 PMC9820834 36615163

[8] Peng J, Zhao F, Yang X, Pan X, Xin J, Wu M, et al. Association between dyslipidemia and risk of type 2 diabetes mellitus in middle-aged and older Chinese adults: a secondary analysis of a nationwide cohort. BMJ Open. 2021;11(5):e042821. doi: 10.1136/bmjopen-2020-042821 34035089 PMC8154929

[9] Dowla S, Aslibekyan S, Goss A, Fontaine K, Ashraf AP. Dyslipidemia is associated with pediatric nonalcoholic fatty liver disease. J Clin Lipidol. 2018;12(4):981–7. doi: 10.1016/j.jacl.2018.03.089 29699915 PMC8513128

[10] Neshat S, Rezaei A, Farid A, Sarallah R, Javanshir S, Ahmadian S, et al. The tangled web of dyslipidemia and cancer: Is there any association?. J Res Med Sci. 2022;27:93. doi: 10.4103/jrms.jrms_267_22 36685020 PMC9854911

[11] Pih GY, Gong EJ, Choi JY, Kim M-J, Ahn JY, Choe J, et al. Associations of Serum Lipid Level with Gastric Cancer Risk, Pathology, and Prognosis. Cancer Res Treat. 2021;53(2):445–56. doi: 10.4143/crt.2020.599 33253515 PMC8053878

[12] Li B, Li M, Qi X, Tong T, Zhang G. The causal associations of circulating lipids with Barrett’s Esophagus and Esophageal Cancer: a bi-directional, two sample mendelian randomization analysis. Hum Genomics. 2024;18(1):37. doi: 10.1186/s40246-024-00608-6 38627859 PMC11020202

[13] Zhang Z, Xu S, Song M, Huang W, Yan M, Li X. Association between blood lipid levels and the risk of liver cancer: a systematic review and meta-analysis. Cancer Causes Control. 2024;35(6):943–53. doi: 10.1007/s10552-024-01853-9 38376693 PMC11129988

[14] Mollinedo F, Gajate C. Lipid rafts as signaling hubs in cancer cell survival/death and invasion: implications in tumor progression and therapy: Thematic Review Series: Biology of Lipid Rafts. J Lipid Res. 2020;61(5):611–35. doi: 10.1194/jlr.TR119000439 33715811 PMC7193951

[15] Jahn KA, Su Y, Braet F. Multifaceted nature of membrane microdomains in colorectal cancer. World J Gastroenterol. 2011;17(6):681–90. doi: 10.3748/wjg.v17.i6.681 21390137 PMC3042645

[16] Mordzińska-Rak A, Verdeil G, Hamon Y, Błaszczak E, Trombik T. Dysregulation of cholesterol homeostasis in cancer pathogenesis. Cell Mol Life Sci. 2025;82(1):168. doi: 10.1007/s00018-025-05617-9 40257622 PMC12011706

[17] Samuel VT, Shulman GI. Mechanisms for insulin resistance: common threads and missing links. Cell. 2012;148(5):852–71. doi: 10.1016/j.cell.2012.02.017 22385956 PMC3294420

[18] Floyd S, Favre C, Lasorsa FM, Leahy M, Trigiante G, Stroebel P, et al. The insulin-like growth factor-I-mTOR signaling pathway induces the mitochondrial pyrimidine nucleotide carrier to promote cell growth. Mol Biol Cell. 2007;18(9):3545–55. doi: 10.1091/mbc.e06-12-1109 17596519 PMC1951771

[19] Yuan F, Wen W, Jia G, Long J, Shu X-O, Zheng W. Serum Lipid Profiles and Cholesterol-Lowering Medication Use in Relation to Subsequent Risk of Colorectal Cancer in the UK Biobank Cohort. Cancer Epidemiol Biomarkers Prev. 2023;32(4):524–30. doi: 10.1158/1055-9965.EPI-22-1170 36780218

[20] Bardelčíková A, Šoltys J, Mojžiš J. Oxidative Stress, Inflammation and Colorectal Cancer: An Overview. Antioxidants (Basel). 2023;12(4). 10.3390/antiox12040901 PMC10135609 37107276

[21] Bhaskar S, Shalini V, Helen A. Quercetin regulates oxidized LDL induced inflammatory changes in human PBMCs by modulating the TLR-NF-κB signaling pathway. Immunobiology. 2011;216(3):367–73. 20828867 10.1016/j.imbio.2010.07.011

[22] van Duijnhoven FJB, Bueno-De-Mesquita HB, Calligaro M, Jenab M, Pischon T, Jansen EHJM, et al. Blood lipid and lipoprotein concentrations and colorectal cancer risk in the European Prospective Investigation into Cancer and Nutrition. Gut. 2011;60(8):1094–102. doi: 10.1136/gut.2010.225011 21383385

[23] Ibáñez-Sanz G, Díez-Villanueva A, Riera-Ponsati M, Fernández-Villa T, Fernández Navarro P, Bustamante M, et al. Mendelian randomization analysis rules out disylipidaemia as colorectal cancer cause. Sci Rep. 2019;9(1):13407. doi: 10.1038/s41598-019-49880-w 31527690 PMC6746794

[24] JBI. Critical Appraisal Tools. 2021. https://jbi.global/critical-appraisal-tools

[25] Tierney JF, Stewart LA, Ghersi D, Burdett S, Sydes MR. Practical methods for incorporating summary time-to-event data into meta-analysis. Trials. 2007;8:16. doi: 10.1186/1745-6215-8-16 17555582 PMC1920534

[26] Shim SR, Lee J. Dose-response meta-analysis: application and practice using the R software. Epidemiol Health. 2019;41:e2019006. doi: 10.4178/epih.e2019006 30999740 PMC6635664

[27] Orsini N, Li R, Wolk A, Khudyakov P, Spiegelman D. Meta-analysis for linear and nonlinear dose-response relations: examples, an evaluation of approximations, and software. Am J Epidemiol. 2012;175(1):66–73. doi: 10.1093/aje/kwr265 22135359 PMC3244608

[28] Iso H, Ikeda A, Inoue M, Sato S, Tsugane S, JPHC Study Group. Serum cholesterol levels in relation to the incidence of cancer: the JPHC study cohorts. Int J Cancer. 2009;125(11):2679–86. doi: 10.1002/ijc.24668 19544528

[29] Chyou PH, Nomura AM, Stemmermann GN. A prospective study of colon and rectal cancer among Hawaii Japanese men. Ann Epidemiol. 1996;6(4):276–82. doi: 10.1016/s1047-2797(96)00047-6 8876837

[30] Inoue M, Noda M, Kurahashi N, Iwasaki M, Sasazuki S, Iso H, et al. Impact of metabolic factors on subsequent cancer risk: results from a large-scale population-based cohort study in Japan. Eur J Cancer Prev. 2009;18(3):240–7. doi: 10.1097/CEJ.0b013e3283240460 19491612

[31] Kitahara CM, Berrington de González A, Freedman ND, Huxley R, Mok Y, Jee SH, et al. Total cholesterol and cancer risk in a large prospective study in Korea. J Clin Oncol. 2011;29(12):1592–8. doi: 10.1200/JCO.2010.31.5200 21422422 PMC3082977

[32] Shin A, Joo J, Yang H-R, Bak J, Park Y, Kim J, et al. Risk prediction model for colorectal cancer: National Health Insurance Corporation study, Korea. PLoS One. 2014;9(2):e88079. doi: 10.1371/journal.pone.0088079 24533067 PMC3922771

[33] Li X, Chen H, Wang G, Feng X, Lyu Z, Wei L, et al. Metabolic Syndrome Components and the Risk of Colorectal Cancer: A Population-Based Prospective Study in Chinese Men. Front Oncol. 2019;9:1047. doi: 10.3389/fonc.2019.01047 31681585 PMC6811600

[34] Liu T, Fan Y, Zhang Q, Wang Y, Yao N, Song M, et al. The combination of metabolic syndrome and inflammation increased the risk of colorectal cancer. Inflamm Res. 2022;71(7–8):899–909. doi: 10.1007/s00011-022-01597-9 35715516 PMC9307555

[35] Hsu S-H, Syu D-K, Chen Y-C, Liu C-K, Sun C-A, Chen M. The Association between Hypertriglyceridemia and Colorectal Cancer: A Long-Term Community Cohort Study in Taiwan. Int J Environ Res Public Health. 2022;19(13):7804. doi: 10.3390/ijerph19137804 35805464 PMC9265720

[36] Borena W, Stocks T, Jonsson H, Strohmaier S, Nagel G, Bjørge T, et al. Serum triglycerides and cancer risk in the metabolic syndrome and cancer (Me-Can) collaborative study. Cancer Causes Control. 2011;22(2):291–9. doi: 10.1007/s10552-010-9697-0 21140204

[37] Lu Y, Ness-Jensen E, Hveem K, Martling A. Metabolic predispositions and increased risk of colorectal adenocarcinoma by anatomical location: a large population-based cohort study in Norway. Am J Epidemiol. 2015;182(10):883–93. doi: 10.1093/aje/kwv141 26511906

[38] Gaard M, Tretli S, Urdal P. Blood lipid and lipoprotein levels and the risk of cancer of the colon and rectum. A prospective study of 62,173 Norwegian men and women. Scand J Gastroenterol. 1997;32(2):162–8. doi: 10.3109/00365529709000187 9051877

[39] Strohmaier S, Edlinger M, Manjer J, Stocks T, Bjørge T, Borena W, et al. Total serum cholesterol and cancer incidence in the Metabolic syndrome and Cancer Project (Me-Can). PLoS One. 2013;8(1):e54242. doi: 10.1371/journal.pone.0054242 23372693 PMC3553083

[40] Törnberg SA, Holm LE, Carstensen JM, Eklund GA. Risks of cancer of the colon and rectum in relation to serum cholesterol and beta-lipoprotein. N Engl J Med. 1986;315(26):1629–33. doi: 10.1056/NEJM198612253152601 3785333

[41] Muka T, Kraja B, Ruiter R, de Keyser CE, Hofman A, Stricker BH, et al. Dietary polyunsaturated fatty acids intake modifies the positive association between serum total cholesterol and colorectal cancer risk: the Rotterdam Study. J Epidemiol Community Health. 2016;70(9):881–7. doi: 10.1136/jech-2015-206556 26917548

[42] Ahn J, Lim U, Weinstein SJ, Schatzkin A, Hayes RB, Virtamo J, et al. Prediagnostic total and high-density lipoprotein cholesterol and risk of cancer. Cancer Epidemiol Biomarkers Prev. 2009;18(11):2814–21. doi: 10.1158/1055-9965.EPI-08-1248 19887581 PMC3534759

[43] Katzke VA, Sookthai D, Johnson T, Kühn T, Kaaks R. Blood lipids and lipoproteins in relation to incidence and mortality risks for CVD and cancer in the prospective EPIC-Heidelberg cohort. BMC Med. 2017;15(1):218. doi: 10.1186/s12916-017-0976-4 29254484 PMC5735858

[44] Fang Z, He M, Song M. Serum lipid profiles and risk of colorectal cancer: a prospective cohort study in the UK Biobank. Br J Cancer. 2021;124(3):663–70. doi: 10.1038/s41416-020-01143-6 33139801 PMC7851156

[45] Schatzkin A, Hoover RN, Taylor PR, Ziegler RG, Carter CL, Albanes D, et al. Site-specific analysis of total serum cholesterol and incident cancer in the National Health and Nutrition Examination Survey I Epidemiologic Follow-up Study. Cancer Res. 1988;48(2):452–8. 3335013

[46] Zhang Y, Wu K, Chan AT, Meyerhardt JA, Giovannucci EL. Long-Term Statin Use, Total Cholesterol Level, and Risk of Colorectal Cancer: A Prospective Cohort Study. Am J Gastroenterol. 2022;117(1):158–66. doi: 10.14309/ajg.0000000000001543 34730560 PMC9200604

[47] Chandler PD, Song Y, Lin J, Zhang S, Sesso HD, Mora S, et al. Lipid biomarkers and long-term risk of cancer in the Women’s Health Study. Am J Clin Nutr. 2016;103(6):1397–407. doi: 10.3945/ajcn.115.124321 27099252 PMC4880994

[48] Tsushima M, Nomura AMY, Lee J, Stemmermann GN. Prospective study of the association of serum triglyceride and glucose with colorectal cancer. Dig Dis Sci. 2005;50(3):499–505. doi: 10.1007/s10620-005-2464-5 15810632

[49] Schoen RE, Tangen CM, Kuller LH, Burke GL, Cushman M, Tracy RP, et al. Increased blood glucose and insulin, body size, and incident colorectal cancer. J Natl Cancer Inst. 1999;91(13):1147–54. doi: 10.1093/jnci/91.13.1147 10393723

[50] Ahmed RL, Schmitz KH, Anderson KE, Rosamond WD, Folsom AR. The metabolic syndrome and risk of incident colorectal cancer. Cancer. 2006;107(1):28–36. doi: 10.1002/cncr.21950 16721800

[51] Mordzińska-Rak A, Verdeil G, Hamon Y, Błaszczak E, Trombik T. Dysregulation of cholesterol homeostasis in cancer pathogenesis. Cell Mol Life Sci. 2025;82(1):168. doi: 10.1007/s00018-025-05617-9 40257622 PMC12011706

[52] Iso H, Ikeda A, Inoue M, Sato S, Tsugane S, JPHC Study Group. Serum cholesterol levels in relation to the incidence of cancer: the JPHC study cohorts. Int J Cancer. 2009;125(11):2679–86. doi: 10.1002/ijc.24668 19544528

[53] Ahn J, Lim U, Weinstein SJ, Schatzkin A, Hayes RB, Virtamo J, et al. Prediagnostic total and high-density lipoprotein cholesterol and risk of cancer. Cancer Epidemiol Biomarkers Prev. 2009;18(11):2814–21. doi: 10.1158/1055-9965.EPI-08-1248 19887581 PMC3534759

[54] Vitols S, Gahrton G, Björkholm M, Peterson C. Hypocholesterolaemia in malignancy due to elevated low-density-lipoprotein-receptor activity in tumour cells: evidence from studies in patients with leukaemia. Lancet. 1985;2(8465):1150–4. doi: 10.1016/s0140-6736(85)92679-0 2865616

[55] Schatzkin A, Hoover RN, Taylor PR, Ziegler RG, Carter CL, Albanes D, et al. Site-specific analysis of total serum cholesterol and incident cancer in the National Health and Nutrition Examination Survey I Epidemiologic Follow-up Study. Cancer Res. 1988;48(2):452–8. 3335013

[56] Chyou PH, Nomura AM, Stemmermann GN. A prospective study of colon and rectal cancer among Hawaii Japanese men. Ann Epidemiol. 1996;6(4):276–82. doi: 10.1016/s1047-2797(96)00047-6 8876837

[57] Pandey H, Tang DWT, Wong SH, Lal D. Gut Microbiota in Colorectal Cancer: Biological Role and Therapeutic Opportunities. Cancers (Basel). 2023;15(3):866. doi: 10.3390/cancers15030866 36765824 PMC9913759

[58] Ahmadishoar S, Mones Saeed S, Salih Mahdi M, Mohammed Taher W, Alwan M, Jasem Jawad M, et al. The potential use of bacteria and their derivatives as delivery systems for nanoparticles in the treatment of cancer. Journal of Drug Targeting. 2025:1–34. 10.1080/1061186X.2025.2489979 40186857

[59] Mamtani R, Lewis JD, Scott FI, Ahmad T, Goldberg DS, Datta J, et al. Disentangling the Association between Statins, Cholesterol, and Colorectal Cancer: A Nested Case-Control Study. PLoS Med. 2016;13(4):e1002007. doi: 10.1371/journal.pmed.1002007 27116322 PMC4846028

[60] Kitahara CM, Berrington de González A, Freedman ND, Huxley R, Mok Y, Jee SH, et al. Total cholesterol and cancer risk in a large prospective study in Korea. J Clin Oncol. 2011;29(12):1592–8. doi: 10.1200/JCO.2010.31.5200 21422422 PMC3082977

[61] Törnberg SA, Holm LE, Carstensen JM, Eklund GA. Risks of cancer of the colon and rectum in relation to serum cholesterol and beta-lipoprotein. N Engl J Med. 1986;315(26):1629–33. doi: 10.1056/NEJM198612253152601 3785333

[62] Yang Z, Tang H, Lu S, Sun X, Rao B. Relationship between serum lipid level and colorectal cancer: a systemic review and meta-analysis. BMJ Open. 2022;12(6):e052373. doi: 10.1136/bmjopen-2021-052373 35732386 PMC9226934

[63] Ding X, Zhang W, Li S, Yang H. The role of cholesterol metabolism in cancer. Am J Cancer Res. 2019;9(2):219–27. 30906624 PMC6405981

[64] Jacobs DR Jr, Hebert B, Schreiner PJ, Sidney S, Iribarren C, Hulley S. Reduced cholesterol is associated with recent minor illness: the CARDIA Study. Coronary Artery Risk Development in Young Adults. Am J Epidemiol. 1997;146(7):558–64. doi: 10.1093/oxfordjournals.aje.a009314 9326433

[65] Brown AJ. Cholesterol, statins and cancer. Clin Exp Pharmacol Physiol. 2007;34(3):135–41. 17250629 10.1111/j.1440-1681.2007.04565.x

[66] Zhang K-L, Zhu W-W, Wang S-H, Gao C, Pan J-J, Du Z-G, et al. Organ-specific cholesterol metabolic aberration fuels liver metastasis of colorectal cancer. Theranostics. 2021;11(13):6560–72. doi: 10.7150/thno.55609 33995676 PMC8120208

[67] Huang T-X, Huang H-S, Dong S-W, Chen J-Y, Zhang B, Li H-H, et al. ATP6V0A1-dependent cholesterol absorption in colorectal cancer cells triggers immunosuppressive signaling to inactivate memory CD8+ T cells. Nat Commun. 2024;15(1):5680. doi: 10.1038/s41467-024-50077-7 38971819 PMC11227557

[68] Tsushima M, Nomura AMY, Lee J, Stemmermann GN. Prospective study of the association of serum triglyceride and glucose with colorectal cancer. Dig Dis Sci. 2005;50(3):499–505. doi: 10.1007/s10620-005-2464-5 15810632

[69] Lu Y, Ness-Jensen E, Hveem K, Martling A. Metabolic predispositions and increased risk of colorectal adenocarcinoma by anatomical location: a large population-based cohort study in Norway. Am J Epidemiol. 2015;182(10):883–93. doi: 10.1093/aje/kwv141 26511906

[70] Bruce WR, Wolever TM, Giacca A, Mechanisms linking diet and colorectal cancer: the possible role of insulin resistance. Nutr Cancer. 2000;37(1):19–26. 10965515 10.1207/S15327914NC3701_2

[71] Inoue M, Noda M, Kurahashi N, Iwasaki M, Sasazuki S, Iso H, et al. Impact of metabolic factors on subsequent cancer risk: results from a large-scale population-based cohort study in Japan. Eur J Cancer Prev. 2009;18(3):240–7. doi: 10.1097/CEJ.0b013e3283240460 19491612

[72] Borena W, Stocks T, Jonsson H, Strohmaier S, Nagel G, Bjørge T, et al. Serum triglycerides and cancer risk in the metabolic syndrome and cancer (Me-Can) collaborative study. Cancer Causes Control. 2011;22(2):291–9. doi: 10.1007/s10552-010-9697-0 21140204

[73] Ulmer H, Borena W, Rapp K, Klenk J, Strasak A, Diem G, et al. Serum triglyceride concentrations and cancer risk in a large cohort study in Austria. Br J Cancer. 2009;101(7):1202–6. doi: 10.1038/sj.bjc.6605264 19690552 PMC2768093

[74] Li B, Mi J, Yuan Q. Fatty acid metabolism-related enzymes in colorectal cancer metastasis: from biological function to molecular mechanism. Cell Death Discov. 2024;10(1):350. doi: 10.1038/s41420-024-02126-9 39103344 PMC11300464

[75] Cowey S, Hardy RW. The metabolic syndrome: A high-risk state for cancer?. Am J Pathol. 2006;169(5):1505–22. doi: 10.2353/ajpath.2006.051090 17071576 PMC1780220

[76] McKeown-Eyssen G. Epidemiology of colorectal cancer revisited: are serum triglycerides and/or plasma glucose associated with risk?. Cancer Epidemiol Biomarkers Prev. 1994;3(8):687–95. 7881343

[77] Yarla N, Madka V, Rao C. Targeting Triglyceride Metabolism for Colorectal Cancer Prevention and Therapy. Curr Drug Targets. 2022;23(6):628–35. doi: 10.2174/1389450122666210824150012 34431463

[78] Li X, Chen H, Wang G, Feng X, Lyu Z, Wei L, et al. Metabolic Syndrome Components and the Risk of Colorectal Cancer: A Population-Based Prospective Study in Chinese Men. Front Oncol. 2019;9:1047. doi: 10.3389/fonc.2019.01047 31681585 PMC6811600

[79] Chandler PD, Song Y, Lin J, Zhang S, Sesso HD, Mora S, et al. Lipid biomarkers and long-term risk of cancer in the Women’s Health Study. Am J Clin Nutr. 2016;103(6):1397–407. doi: 10.3945/ajcn.115.124321 27099252 PMC4880994

[80] Sundaram S, Lamichhane R, Cecchetti A, Murughiyan U, Sundaram U. Risk of Colorectal Cancer among Patients with One or Multiple Metabolic Syndrome Components. Cancers (Basel). 2024;16(19):3350. doi: 10.3390/cancers16193350 39409969 PMC11482601

[81] Zamanian-Daryoush M, DiDonato JA. Apolipoprotein A-I and Cancer. Front Pharmacol. 2015;6:265. doi: 10.3389/fphar.2015.00265 26617517 PMC4642354

[82] Soran H, Hama S, Yadav R, Durrington PN. HDL functionality. Curr Opin Lipidol. 2012;23(4):353–66. 22732521 10.1097/MOL.0b013e328355ca25

[83] von Eckardstein A, Hersberger M, Rohrer L. Current understanding of the metabolism and biological actions of HDL. Curr Opin Clin Nutr Metab Care. 2005;8(2):147–52. doi: 10.1097/00075197-200503000-00007 15716792

[84] Stevanovic M, Vekic J, Bogavac-Stanojevic N, Janac J, Stjepanovic Z, Zeljkovic D, et al. Significance of LDL and HDL subclasses characterization in the assessment of risk for colorectal cancer development. Biochem Med (Zagreb). 2018;28(3):030703. doi: 10.11613/BM.2018.030713 30429670 PMC6214700

[85] Hernáez Á, Soria-Florido MT, Schröder H, Ros E, Pintó X, Estruch R, et al. Role of HDL function and LDL atherogenicity on cardiovascular risk: A comprehensive examination. PLoS One. 2019;14(6):e0218533. doi: 10.1371/journal.pone.0218533 31246976 PMC6597156

[86] Bufill JA. Colorectal cancer: evidence for distinct genetic categories based on proximal or distal tumor location. Ann Intern Med. 1990;113(10):779–88. doi: 10.7326/0003-4819-113-10-779 2240880

[87] Ahmed RL, Schmitz KH, Anderson KE, Rosamond WD, Folsom AR. The metabolic syndrome and risk of incident colorectal cancer. Cancer. 2006;107(1):28–36. doi: 10.1002/cncr.21950 16721800

[88] Shin A, Song Y-M, Yoo K-Y, Sung J. Menstrual factors and cancer risk among Korean women. Int J Epidemiol. 2011;40(5):1261–8. doi: 10.1093/ije/dyr121 21841186

[89] al-Azzawi F, Wahab M. Estrogen and colon cancer: current issues. Climacteric. 2002;5(1):3–14. doi: 10.1080/cmt.5.1.3.14 11974557

[90] Liu T, Fan Y, Zhang Q, Wang Y, Yao N, Song M, et al. The combination of metabolic syndrome and inflammation increased the risk of colorectal cancer. Inflamm Res. 2022;71(7–8):899–909. doi: 10.1007/s00011-022-01597-9 35715516 PMC9307555

[91] Zhang Y, Wu K, Chan AT, Meyerhardt JA, Giovannucci EL. Long-Term Statin Use, Total Cholesterol Level, and Risk of Colorectal Cancer: A Prospective Cohort Study. Am J Gastroenterol. 2022;117(1):158–66. doi: 10.14309/ajg.0000000000001543 34730560 PMC9200604

[92] Fang Z, He M, Song M. Serum lipid profiles and risk of colorectal cancer: a prospective cohort study in the UK Biobank. Br J Cancer. 2021;124(3):663–70. doi: 10.1038/s41416-020-01143-6 33139801 PMC7851156

[93] Katzke VA, Sookthai D, Johnson T, Kühn T, Kaaks R. Blood lipids and lipoproteins in relation to incidence and mortality risks for CVD and cancer in the prospective EPIC-Heidelberg cohort. BMC Med. 2017;15(1):218. doi: 10.1186/s12916-017-0976-4 29254484 PMC5735858

[94] Agnoli C, Grioni S, Sieri S, Sacerdote C, Vineis P, Tumino R, et al. Colorectal cancer risk and dyslipidemia: a case-cohort study nested in an Italian multicentre cohort. Cancer Epidemiol. 2014;38(2):144–51. doi: 10.1016/j.canep.2014.02.002 24636241

