# Supplementary material for: The association between serum lipid levels and colorectal cancer risk: A dose-response meta-analysis of 23 studies
Source: PLoS One. 2025 Oct 16;20(10):e0333907. doi: 10.1371/journal.pone.0333907 (PMC12530605; doi:10.1371/journal.pone.0333907)
Supplement: S3 File — (DOCX) [file pone.0333907.s003.docx]

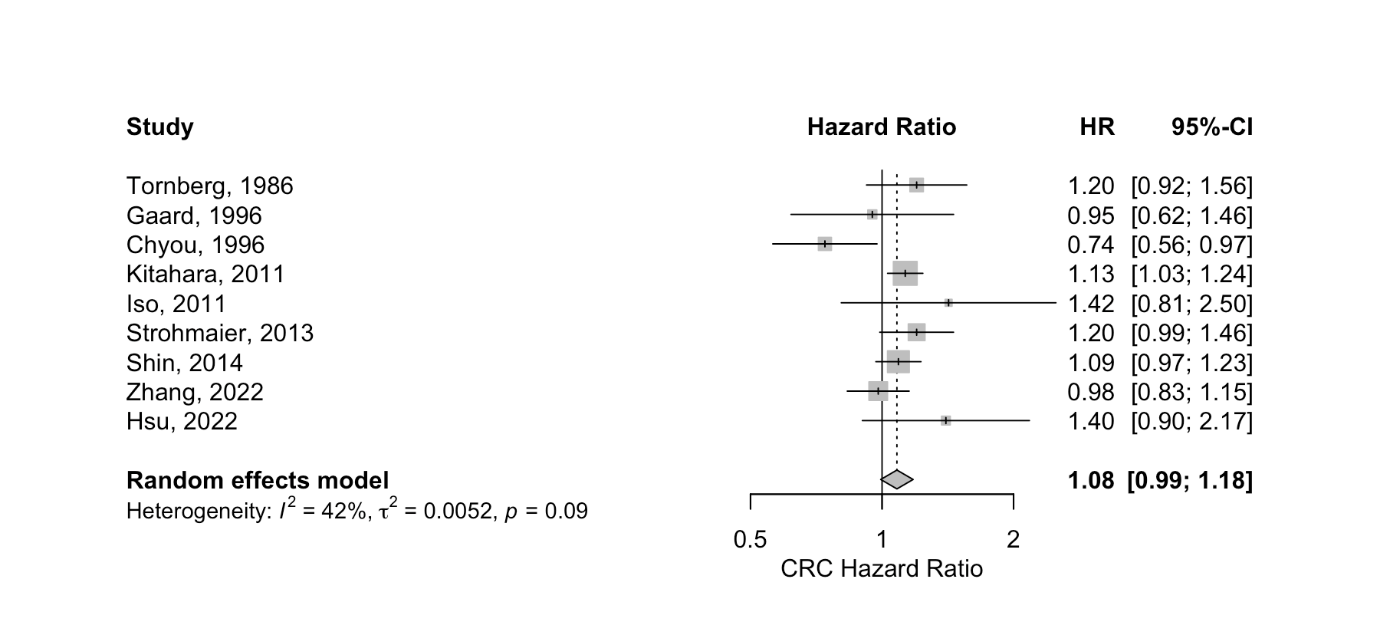


**Figure S1**. The forest plot for the association between serum cholesterol

levels and the risk of colon cancer.

HR, hazards ratio


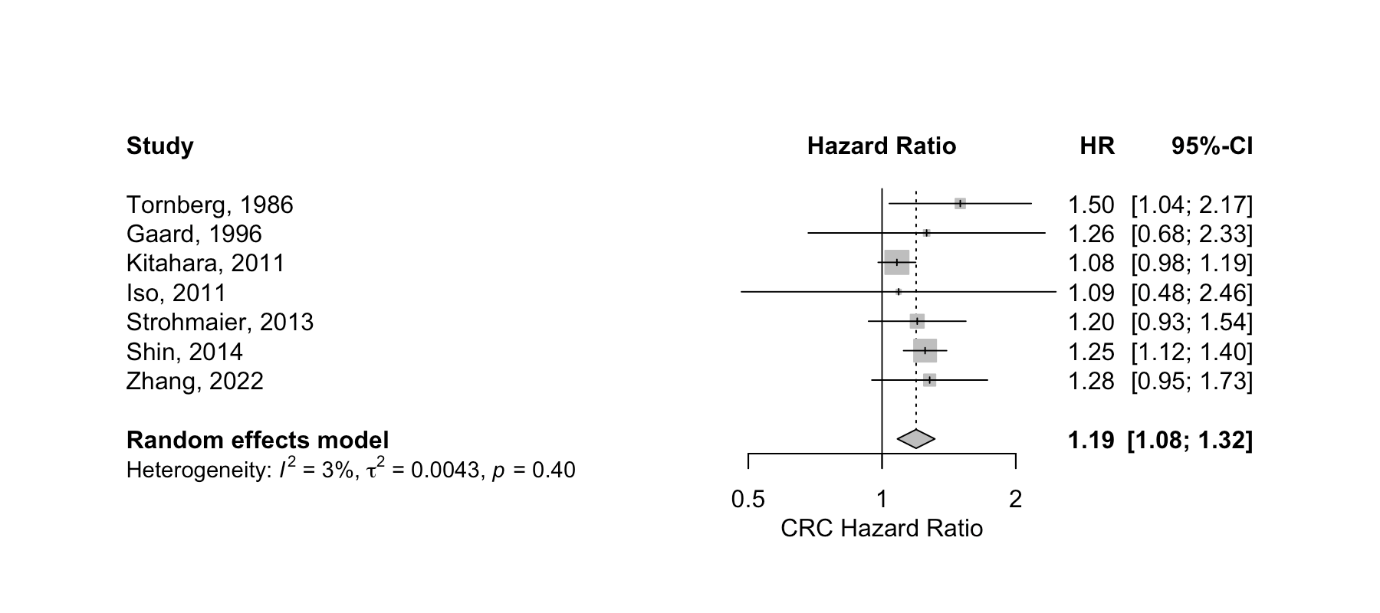
**Figure S2.** The forest plot for the association between serum cholesterol

levels and the risk of rectal cancer.

HR, hazards ratio


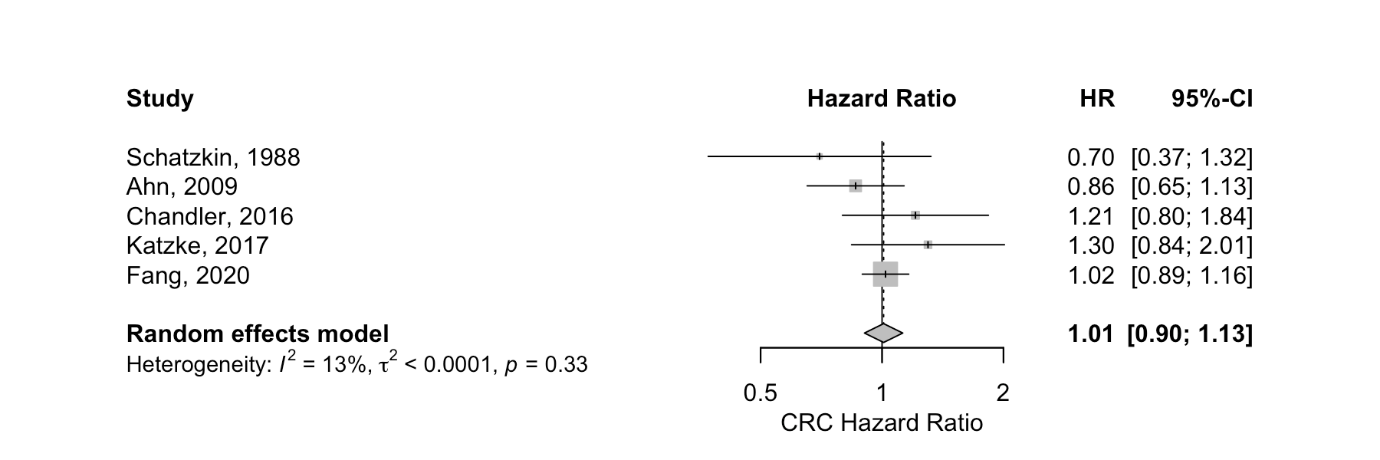
**Figure S3.** The forest plot for the sensitivity analysis for the association between cholesterol and CRC.

HR, hazards ratio.


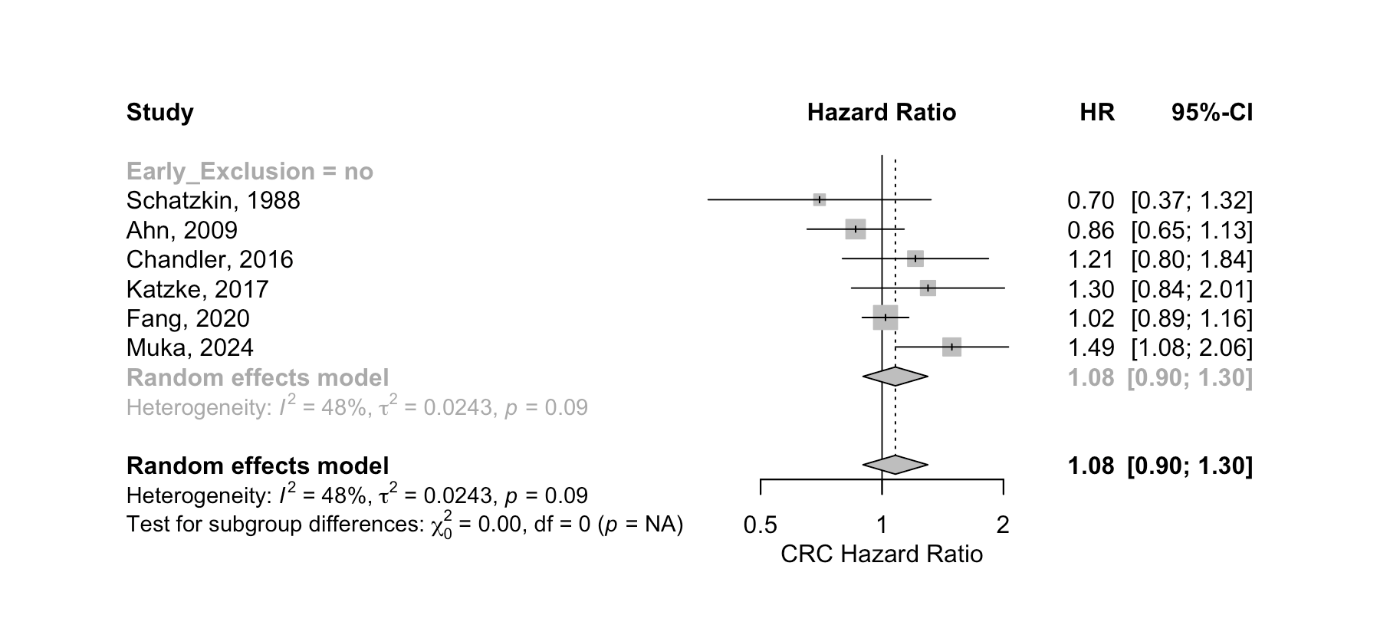


**Figure S4.** The forest plot for the association between serum cholesterol

levels and the risk of colorectal cancer with and without early exclusion.

HR, hazard ratio


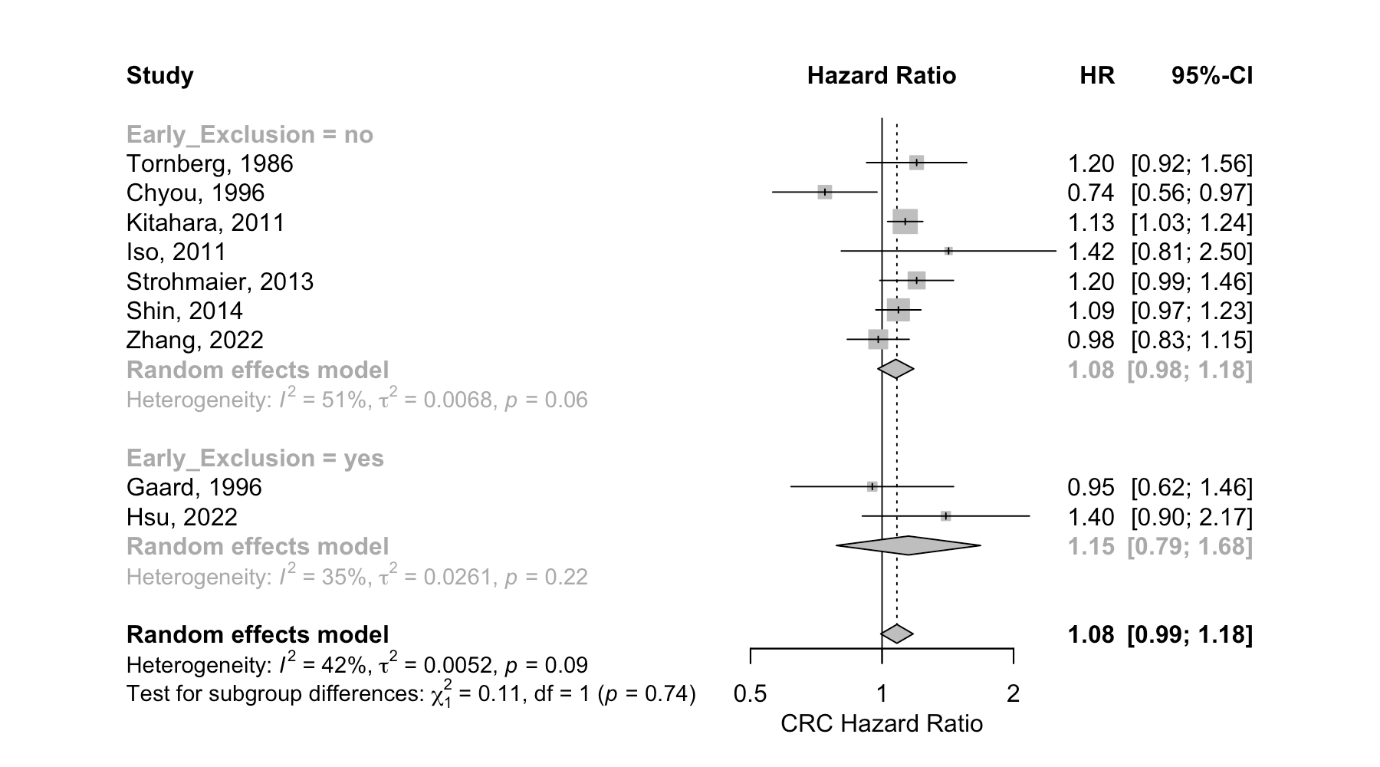
**Figure S5.** The forest plot for the association between serum cholesterol

levels and the risk of colon cancer with and without early exclusion.

HR, hazard ratio


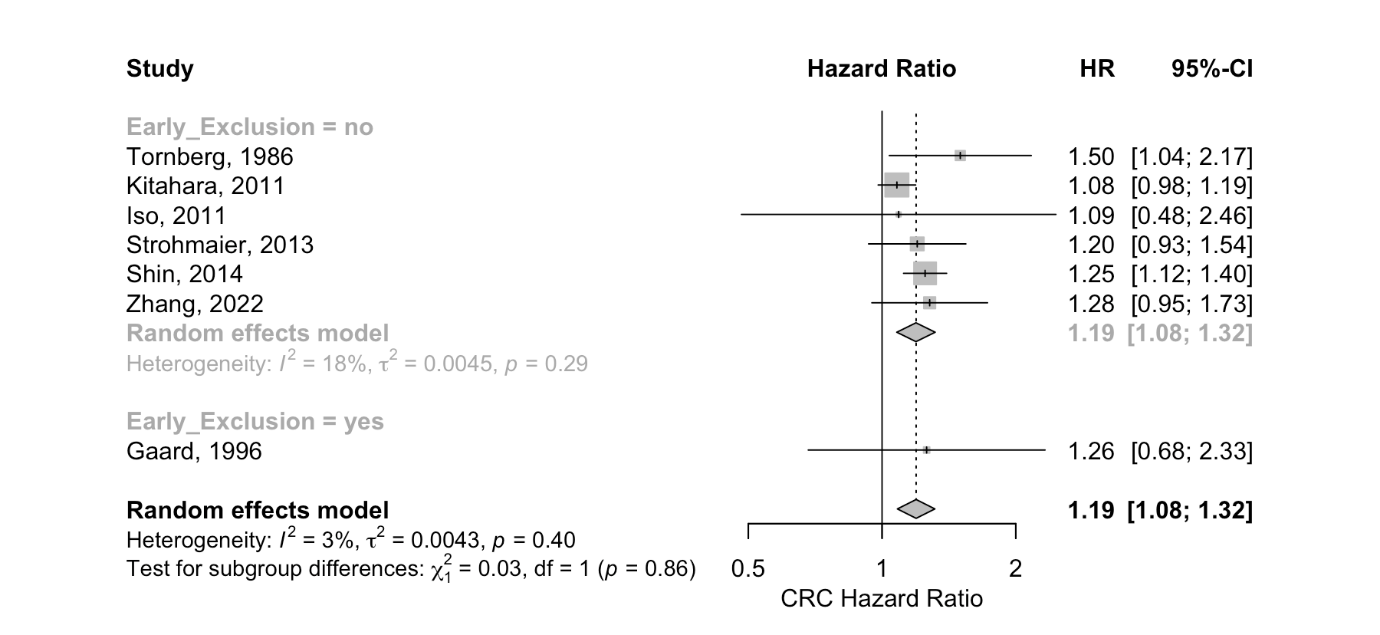


**Figure S6.** The forest plot for the association between serum cholesterol

levels and the risk of rectal cancer with and without early exclusion.

HR, hazard ratio


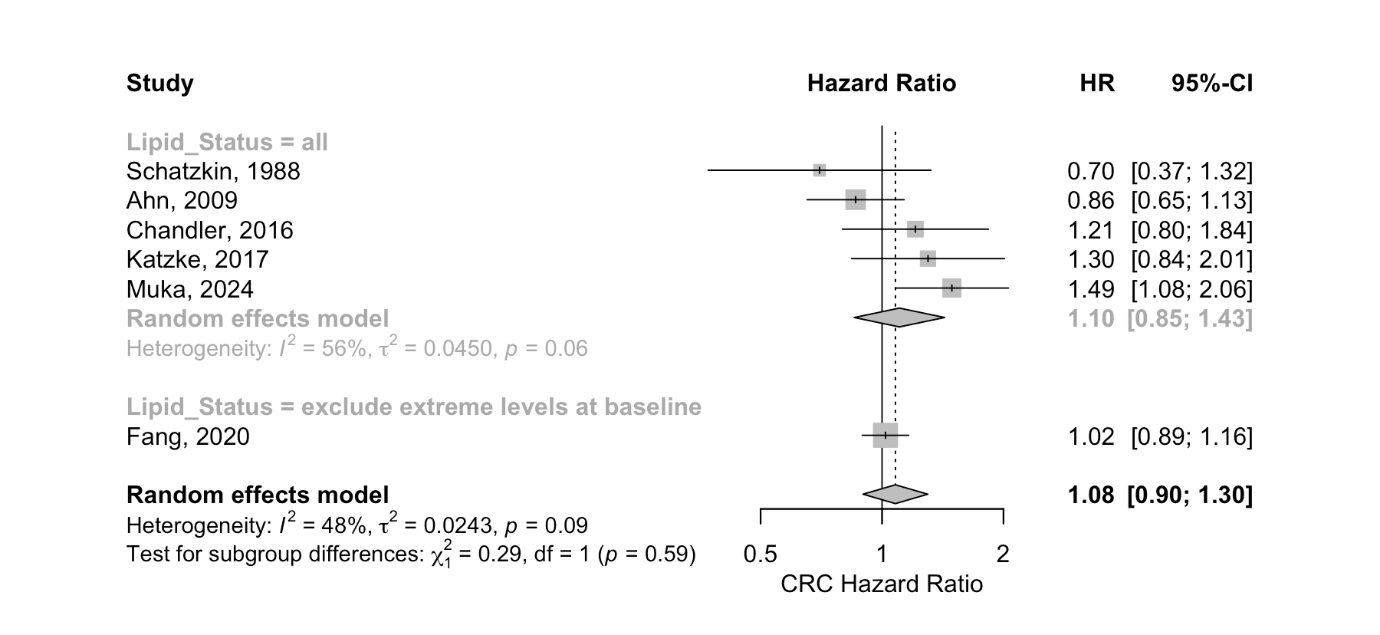
**Figure S7**. The forest plot for the association between serum cholesterol

levels and the risk of colorectal cancer with and without exclusion of extreme levels at baseline.

HR, hazard ratio


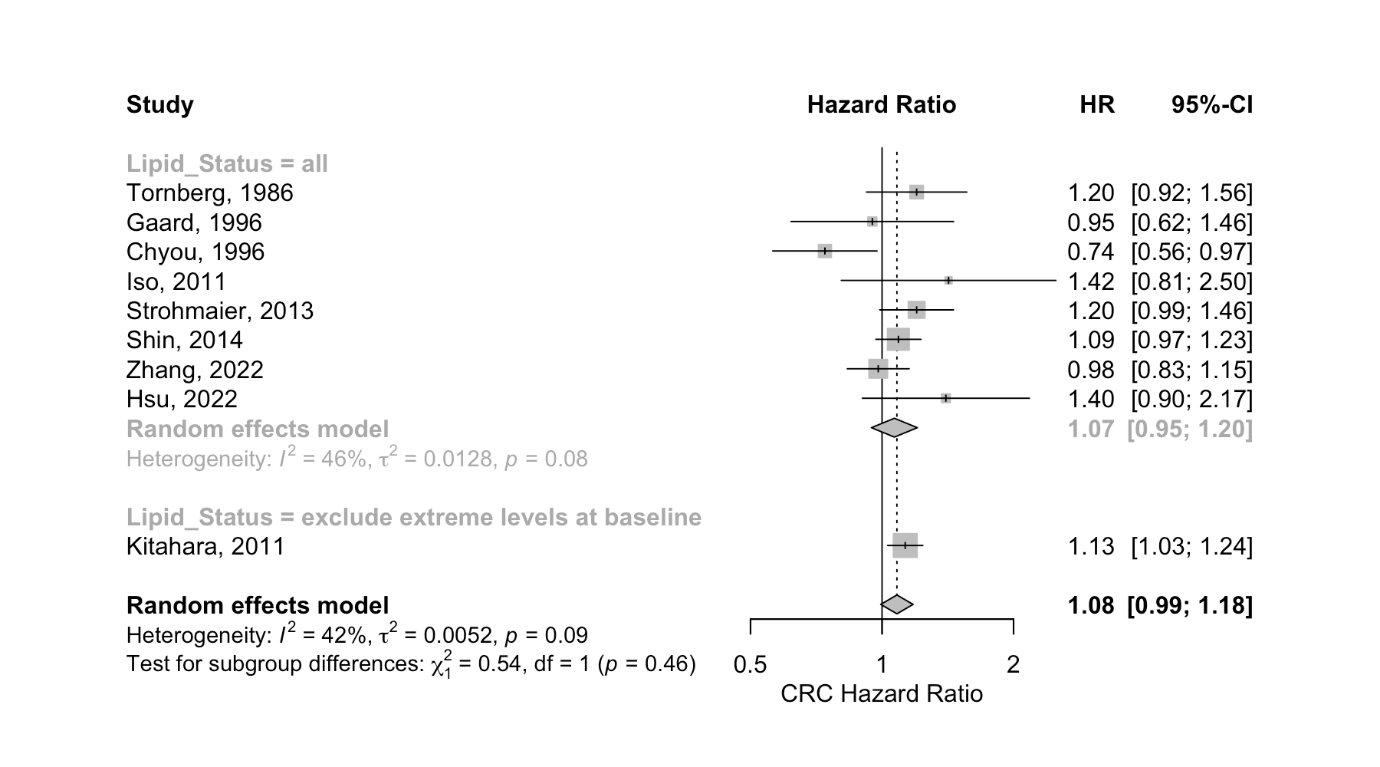
**Figure S8.** The forest plot for the association between serum cholesterol

levels and the risk of colon cancer with and without exclusion of extreme levels at baseline.

HR, hazard ratio


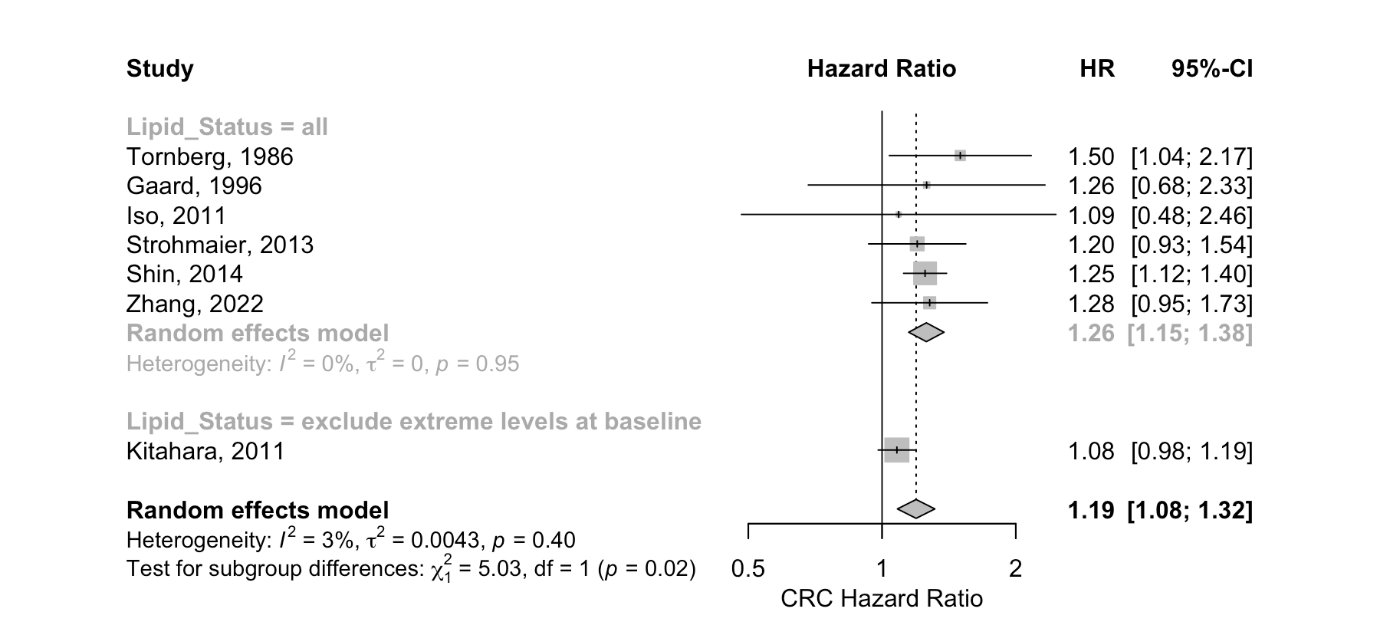
**Figure S9.** The forest plot for the association between serum cholesterol

levels and the risk of rectal cancer with and without exclusion of extreme levels at baseline.

HR, hazard ratio


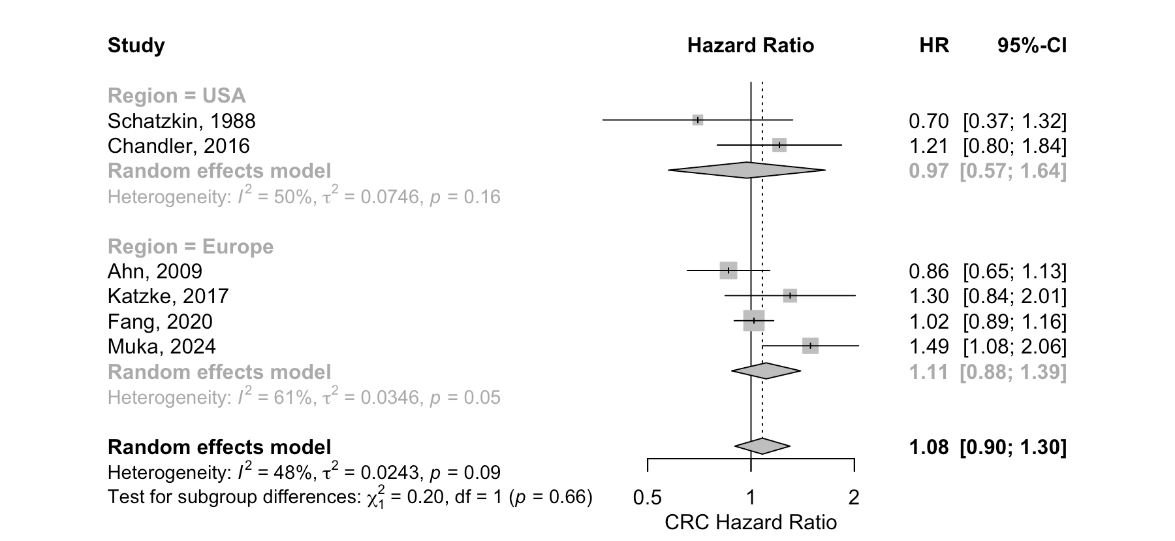


**Figure S10***.* The forest plot for the association between serum cholesterol

levels and the risk of colorectal cancer, categorized

by geographic region. HR, hazard ratio.


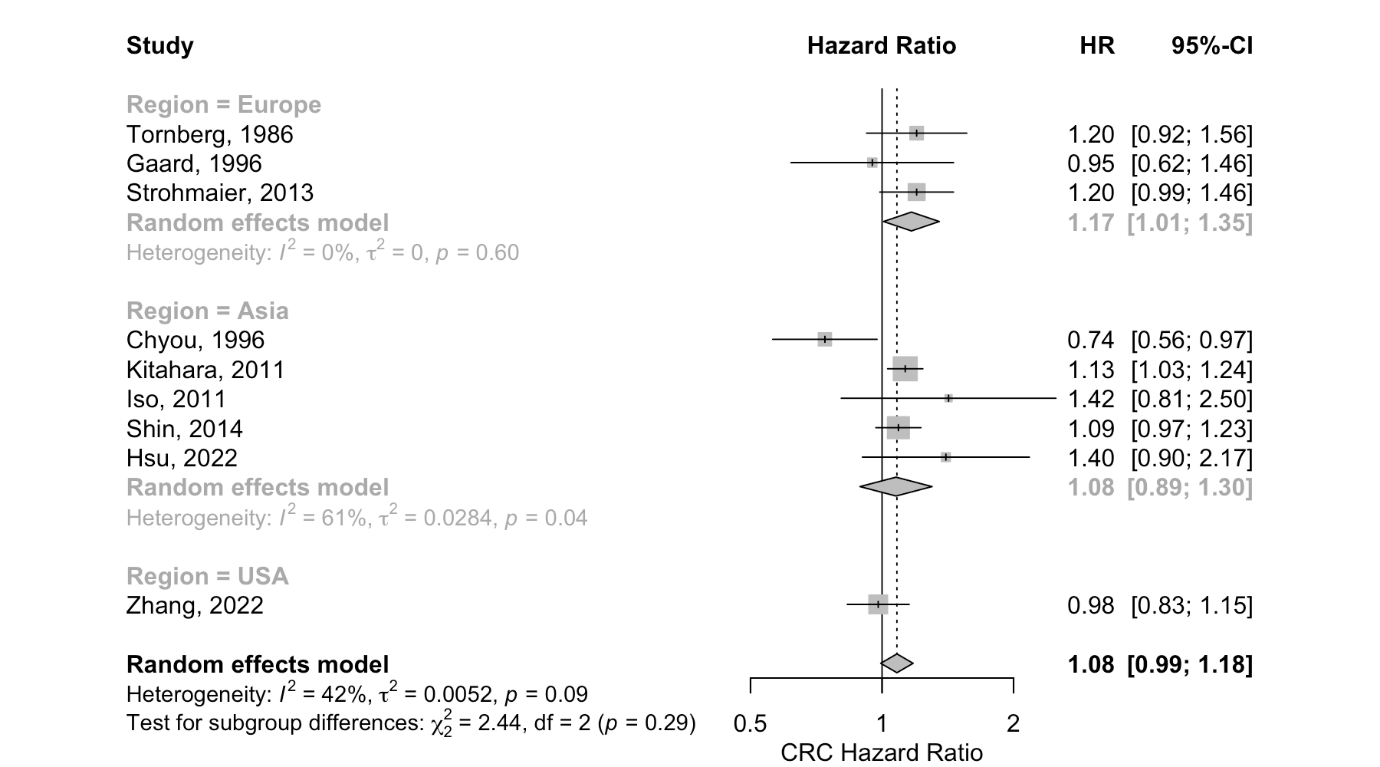


**Figure S11**. The forest plot for the association between serum cholesterol

levels and the risk of colon cancer, categorized

by geographic region. HR, hazard ratio.


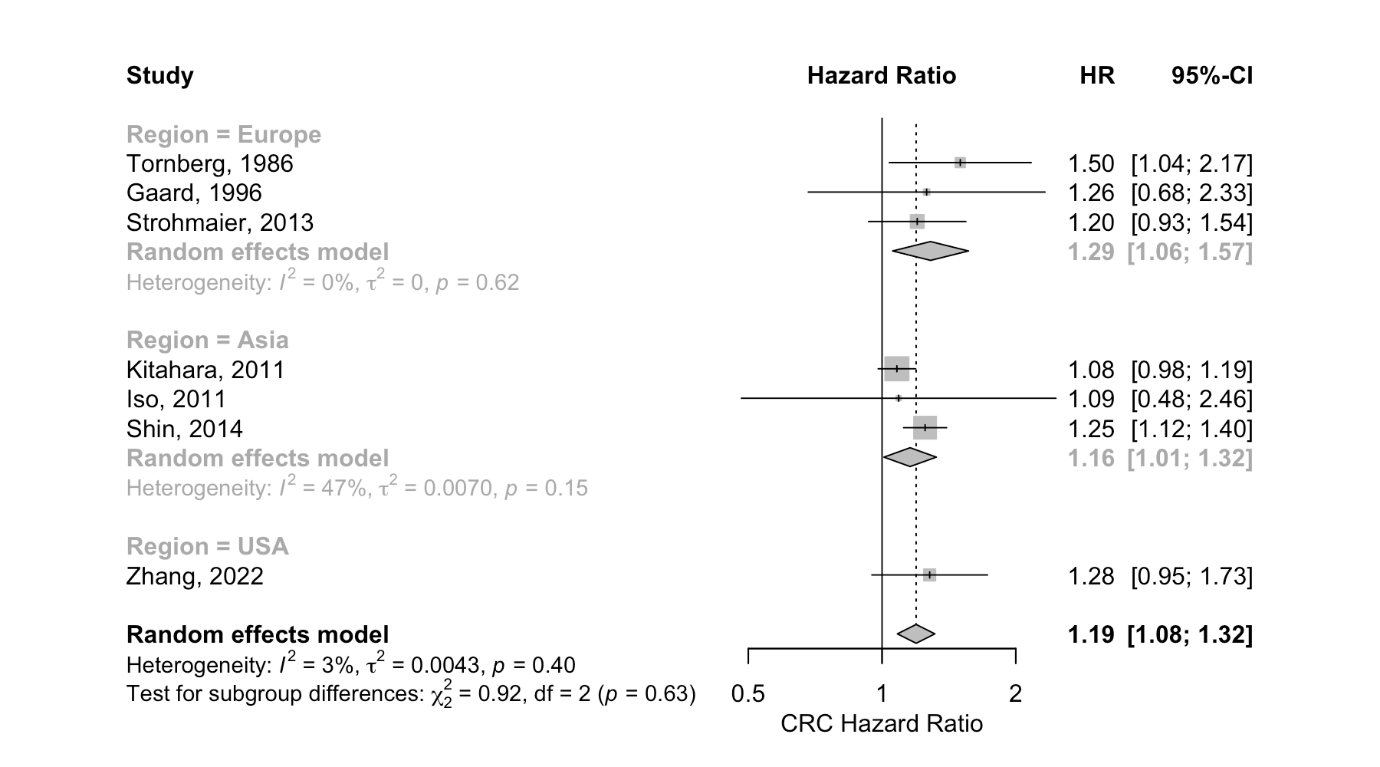


**Figure S12.** The forest plot for the association between serum cholesterol

levels and the risk of rectal cancer, categorized

by geographic region. HR, hazard ratio.


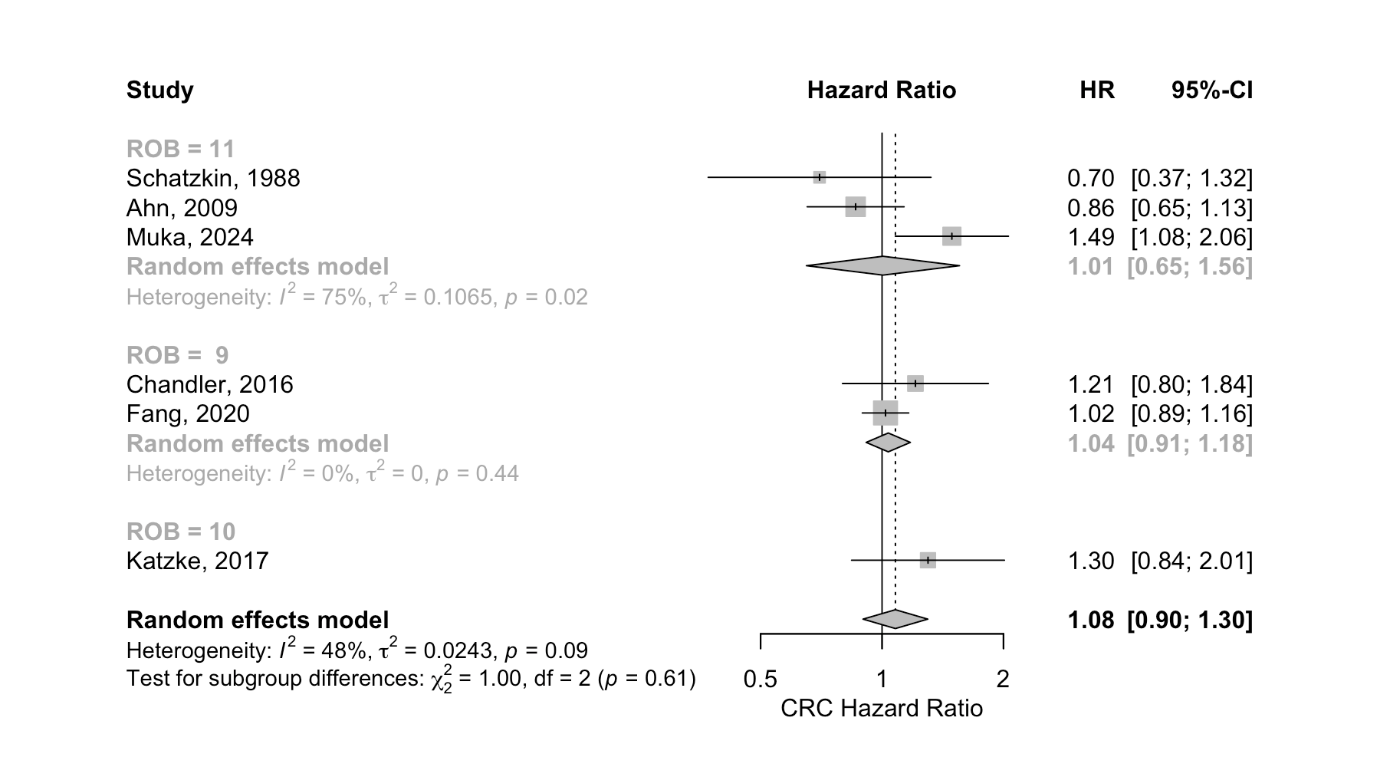


**Figure S13.** The forest plot for the risk of bias analysis of the association of cholesterol and CRC.

HR, hazard ratio.


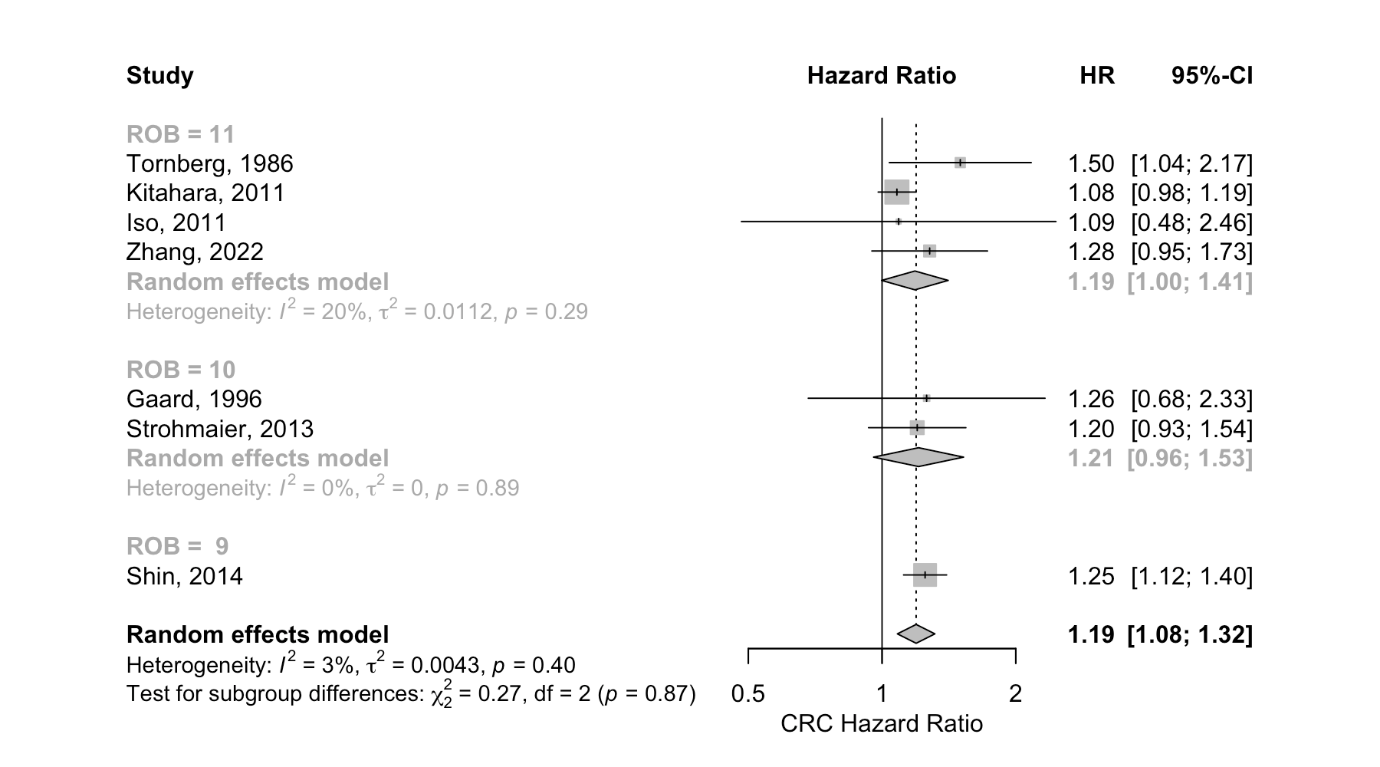


**Figure S14**. The forest plot for the risk of bias analysis of the association of cholesterol and rectal cancer.

HR, hazard ratio.


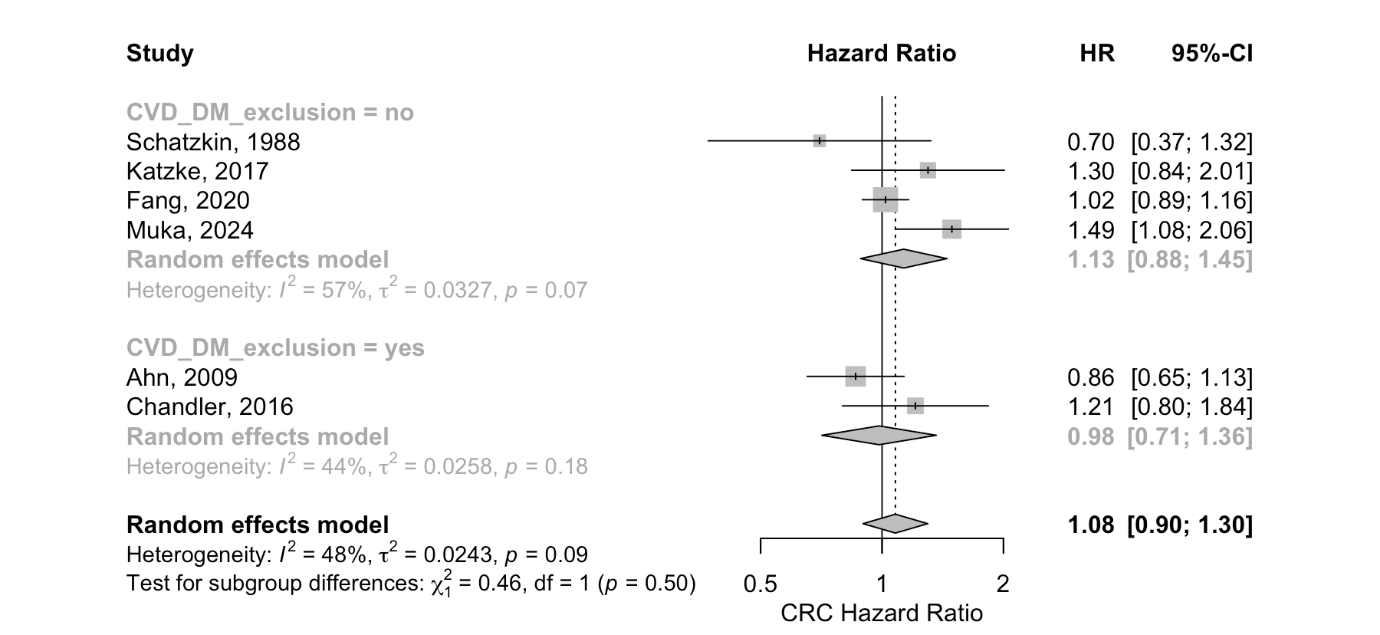


**Figure S15.** The forest plot for the association between serum cholesterol

levels and the risk of colorectal cancer with and without exclusion of cases with cardiovascular diseases and diabetes mellitus.

HR, hazard ratio


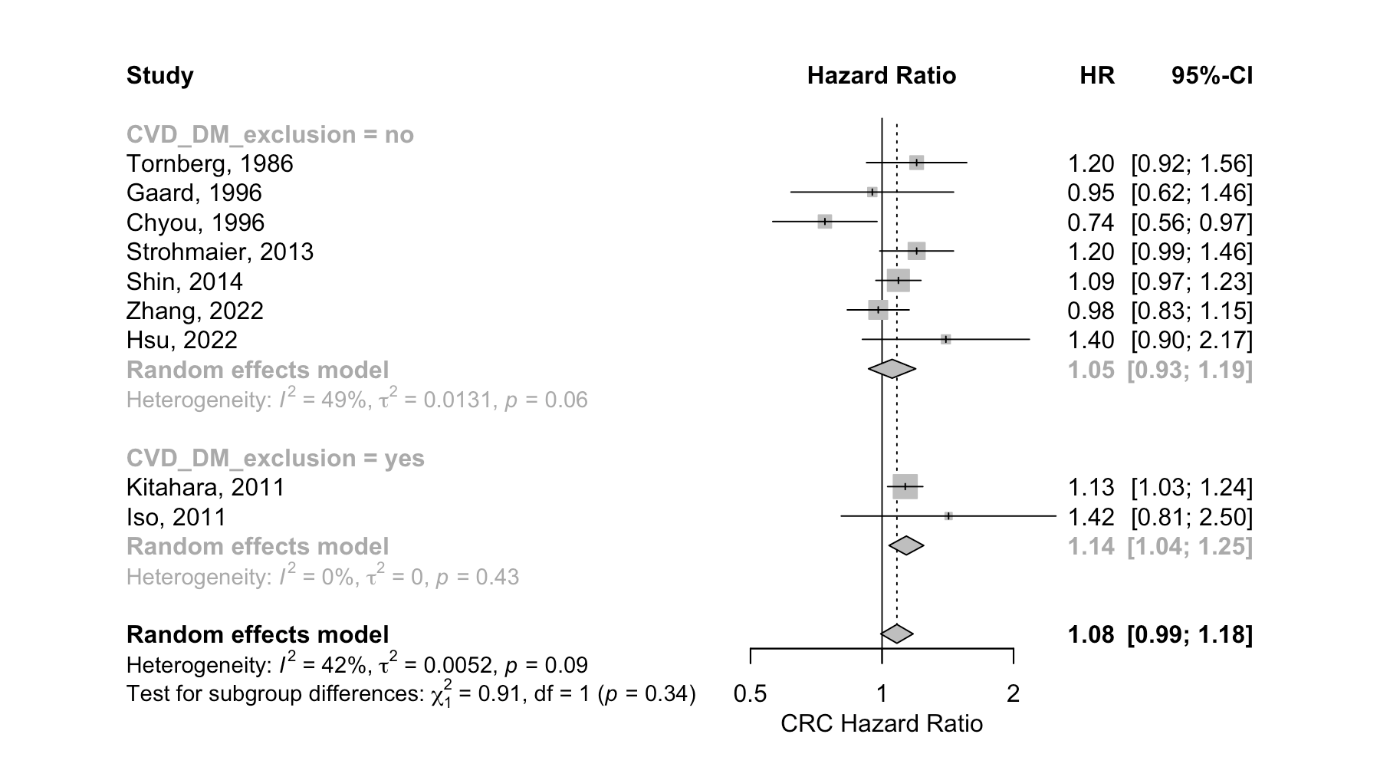


**Figure S16.** The forest plot for the association between serum cholesterol

levels and the risk of colon cancer with and without exclusion of cases with cardiovascular diseases and diabetes mellitus.

HR, hazard ratio


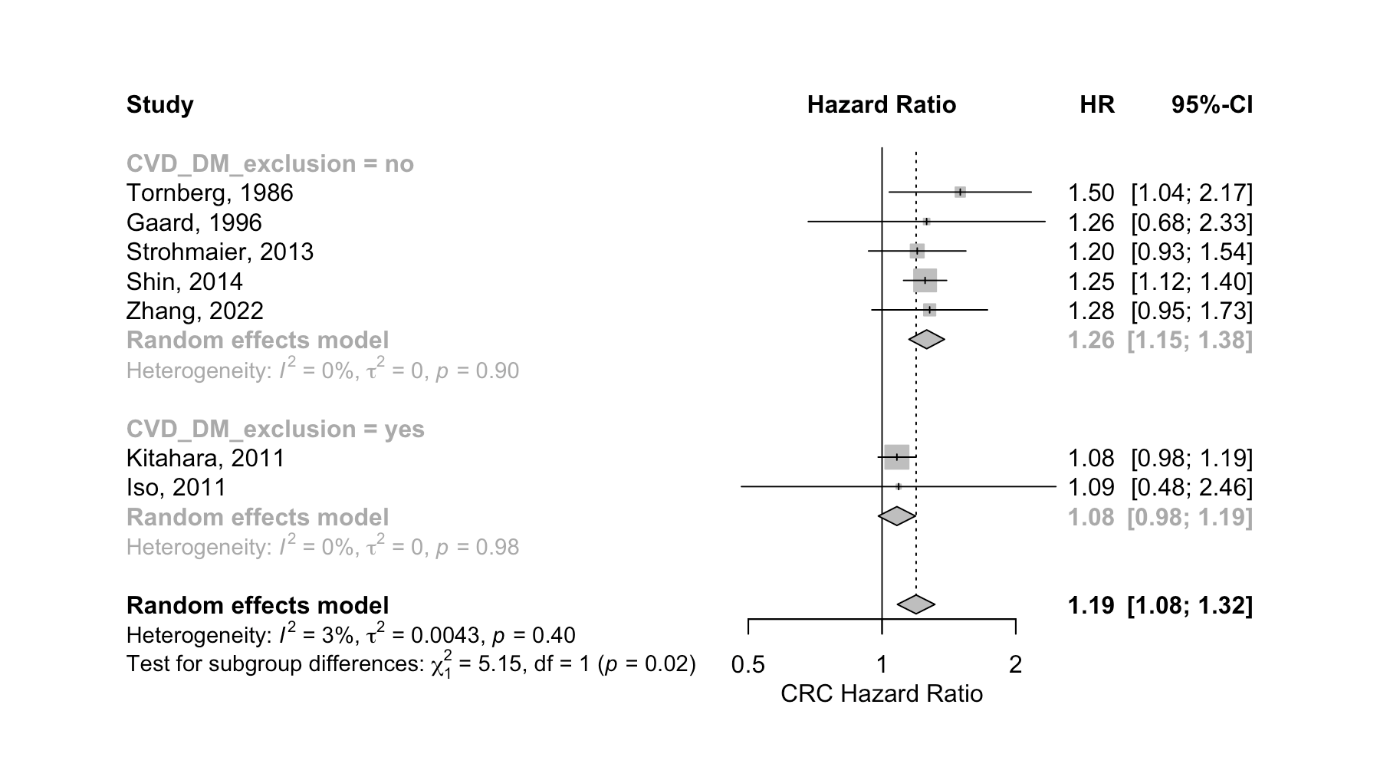
**Figure S17**. The forest plot for the association between serum cholesterol

levels and the risk of rectal cancer with and without exclusion of cases with cardiovascular diseases and diabetes mellitus.

HR, hazard ratio


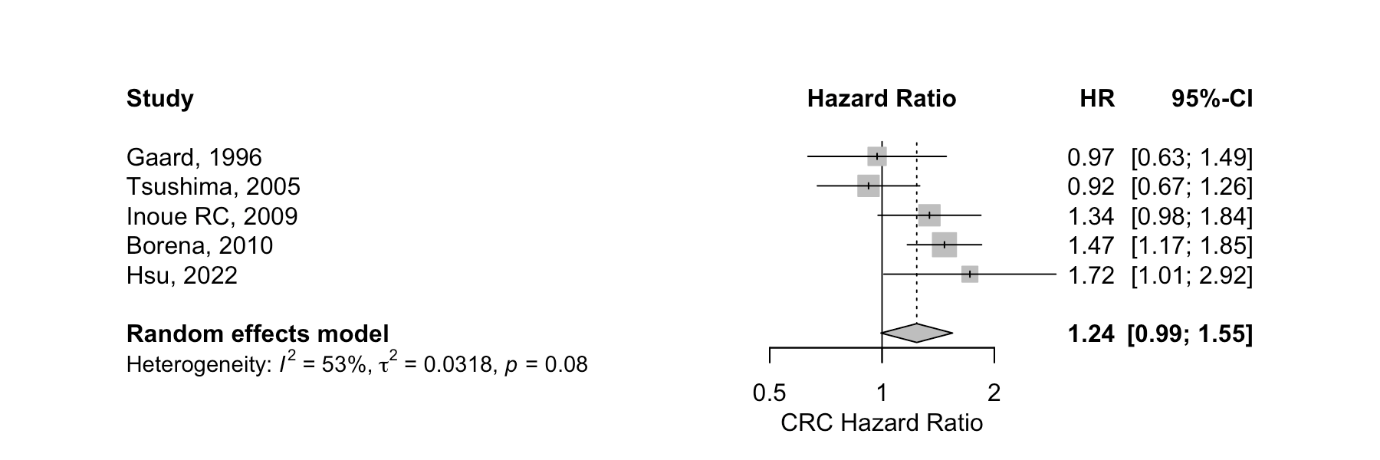


**Figure S18**. The forest plot for the association between serum TG

levels and the risk of colon cancer.

HR, hazards ratio


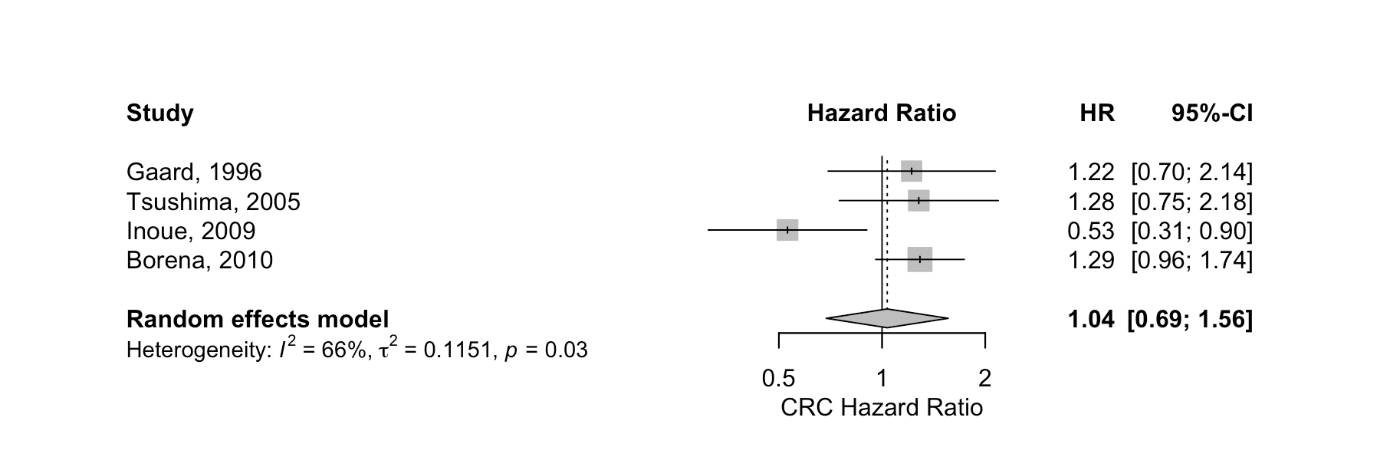


**Figure S19**. The forest plot for the association between serum TG

levels and the risk of rectal cancer. HR, hazards ratio


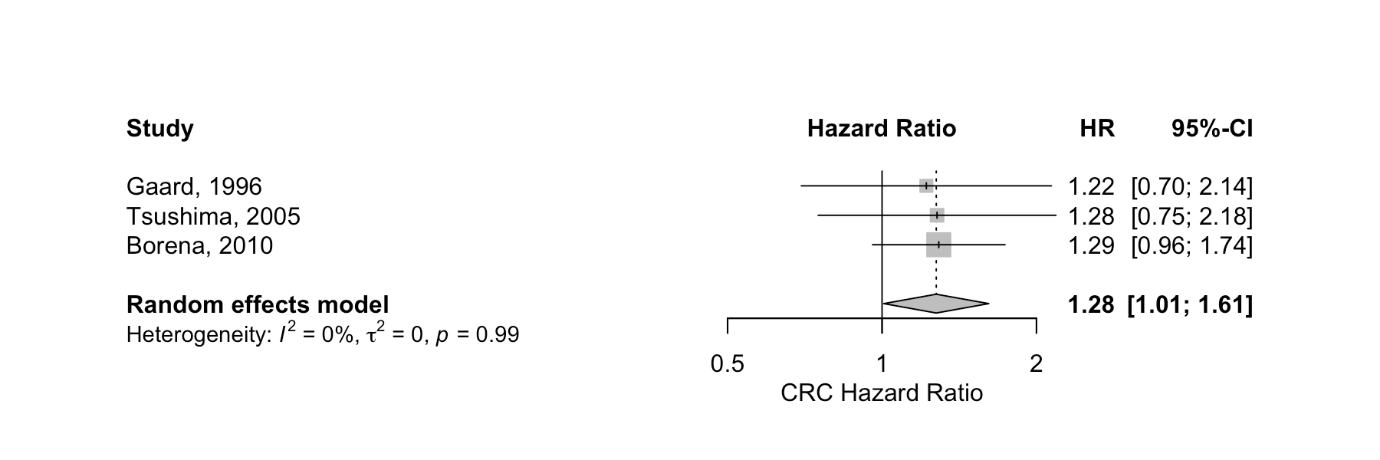
**Figure S20**. The forest plot for the sensitivity analysis for the association between TG and CRC.

HR, hazards ratio.


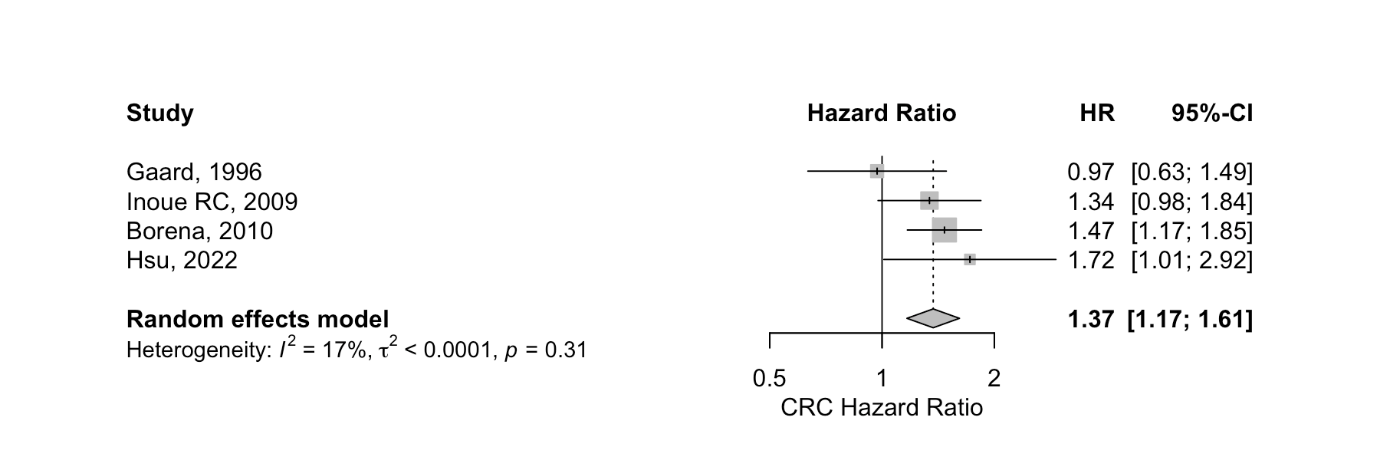
**Figure S21**. The forest plot for the sensitivity analysis for the association between TG and colon cancer.

HR, hazards ratio.


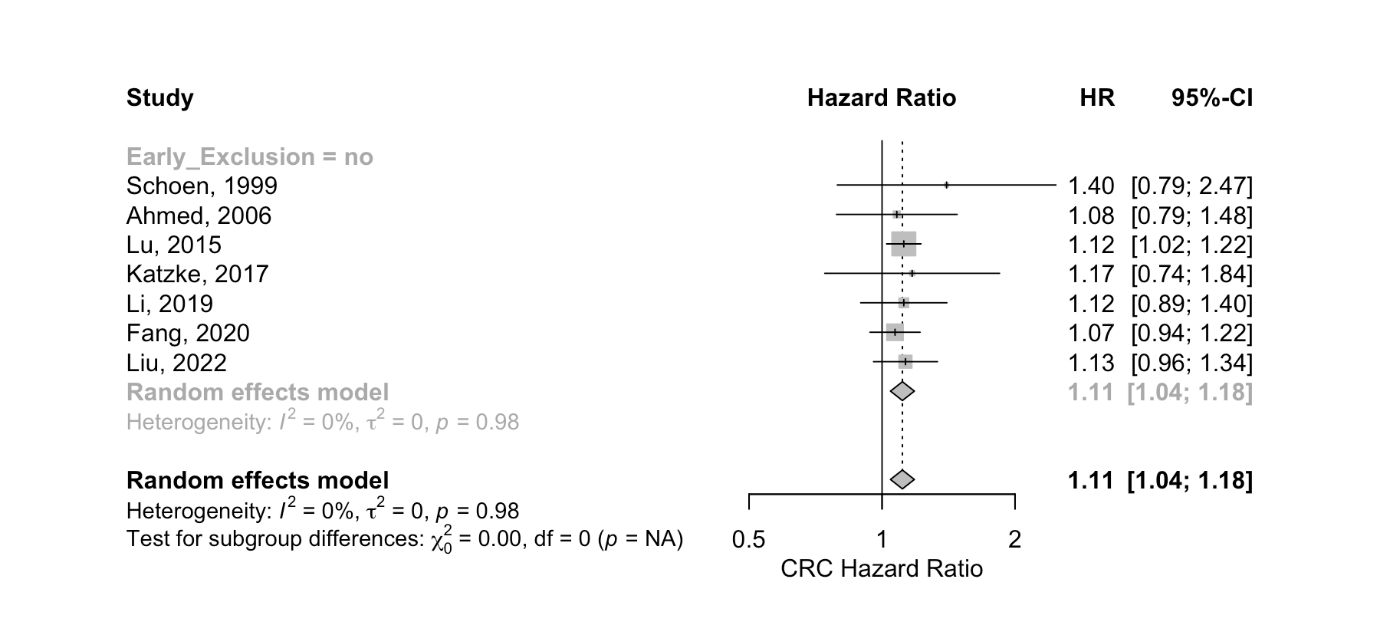


**Figure S22**. The forest plot for the association between serum TG

levels and the risk of colorectal cancer with and without early exclusion.

HR, hazard ratio


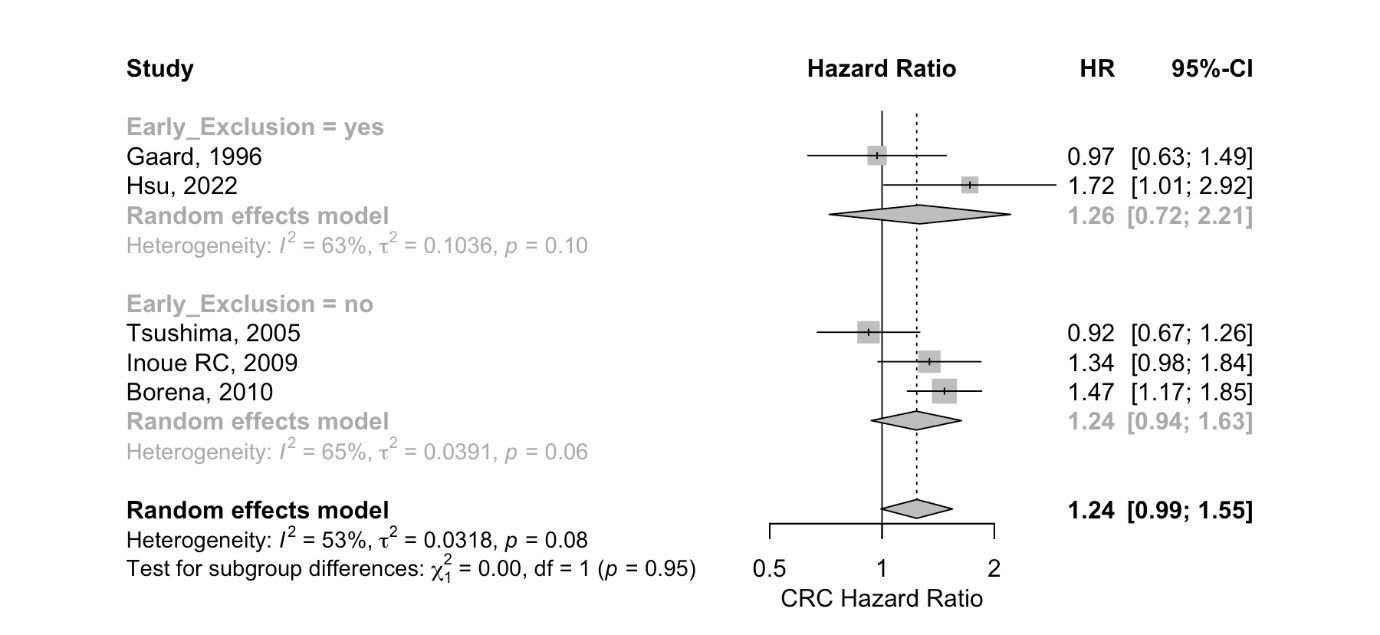
**Figure S23**. The forest plot for the association between serum TG

levels and the risk of colon cancer with and without early exclusion.

HR, hazard ratio


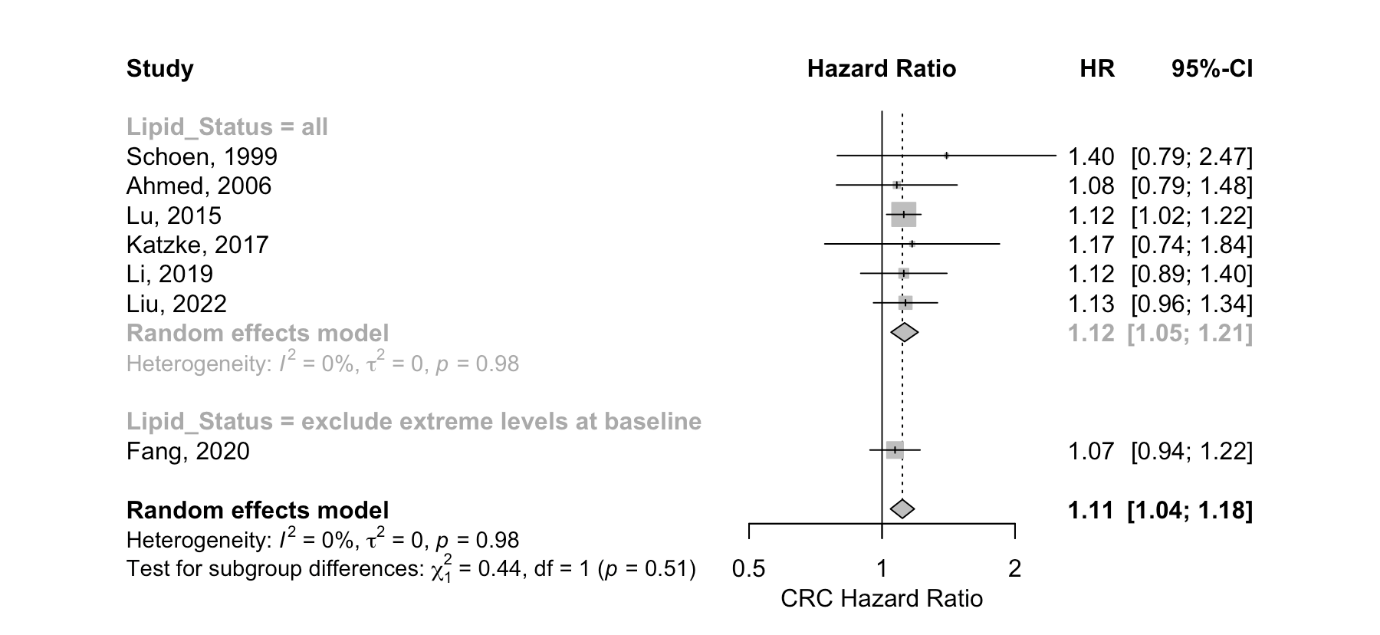
**Figure S24**. The forest plot for the association between serum TG

levels and the risk of colorectal cancer with and without exclusion of extreme levels at baseline.

HR, hazard ratio


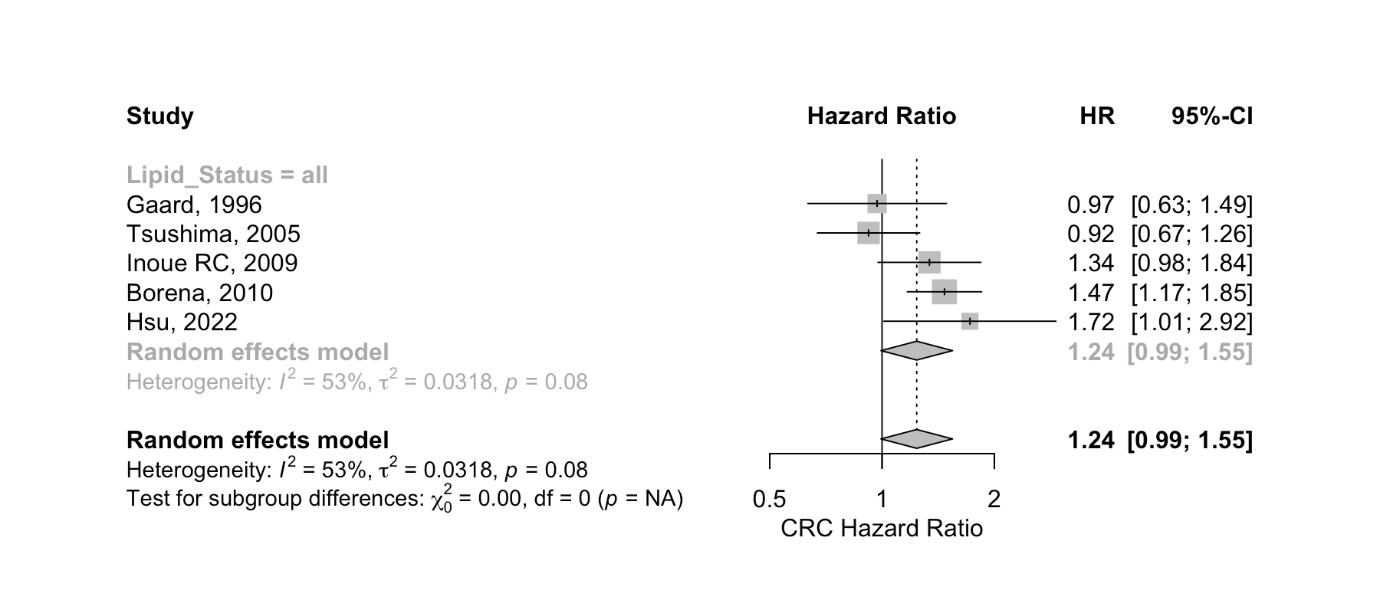


**Figure S25**. The forest plot for the association between serum TG

levels and the risk of colon cancer with and without exclusion of extreme levels at baseline.

HR, hazard ratio


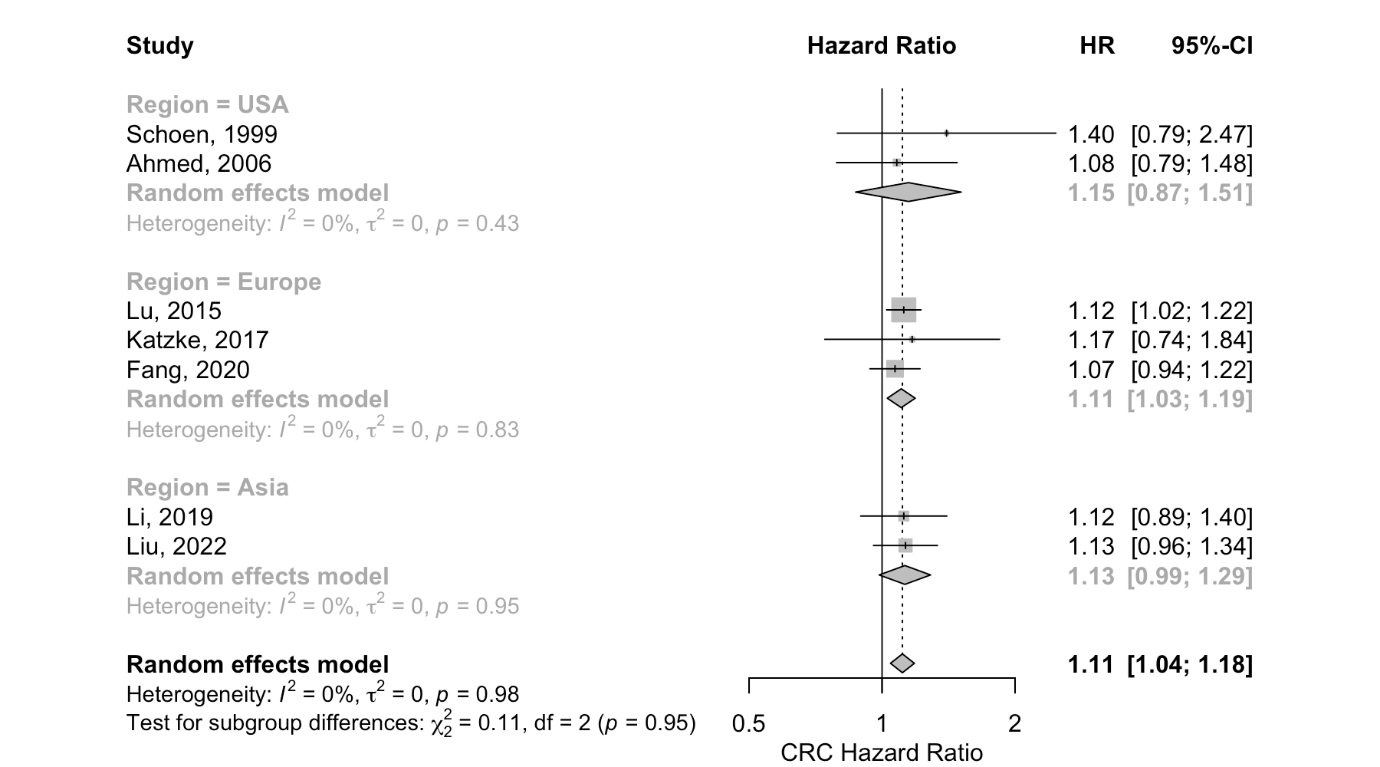
**Figure S26***.* The forest plot for the association between serum TG

levels and the risk of colorectal cancer, categorized

by geographic region. HR, hazard ratio.


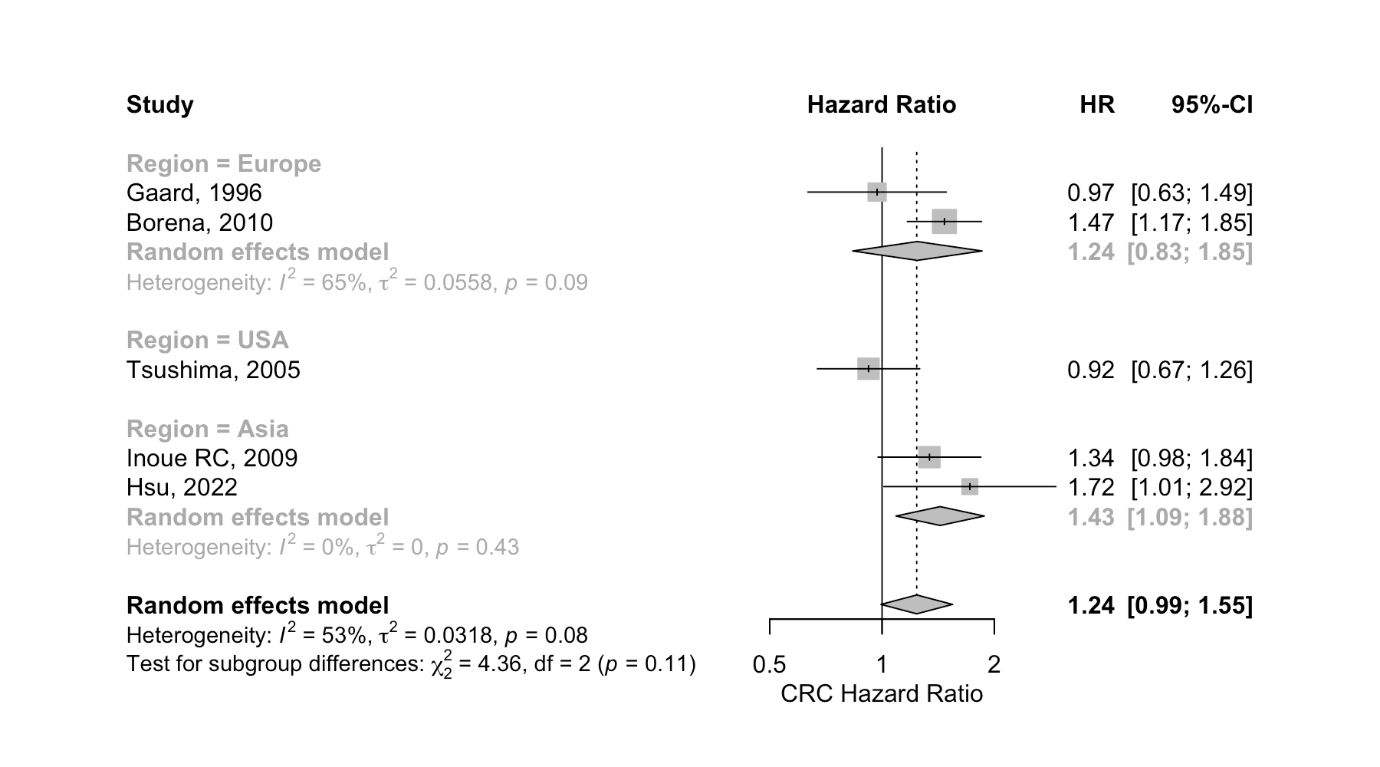
**Figure S27***.* The forest plot for the association between serum TG

levels and the risk of colon cancer, categorized

by geographic region. HR, hazard ratio.


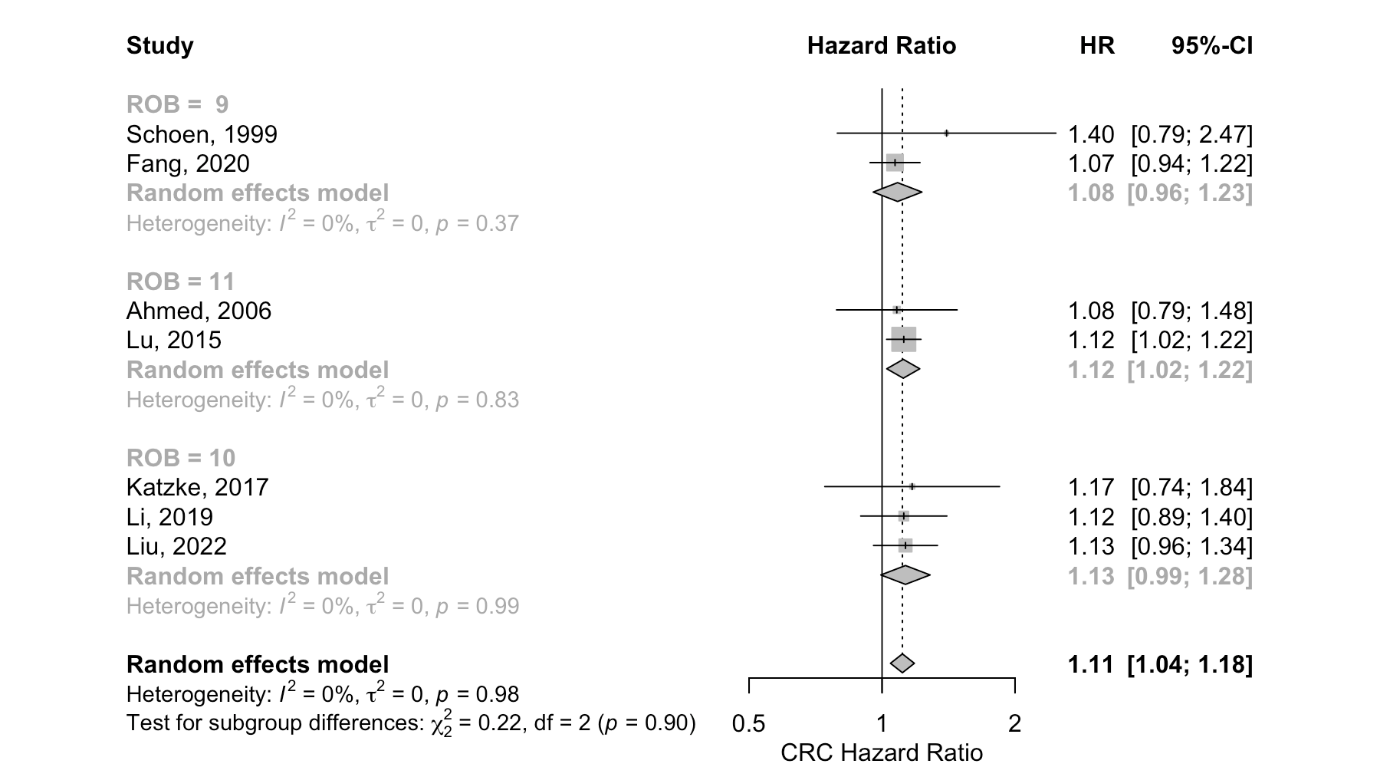


**Figure S28**. The forest plot for the risk of bias analysis of the association of TG and colon cancer.

HR, hazard ratio.


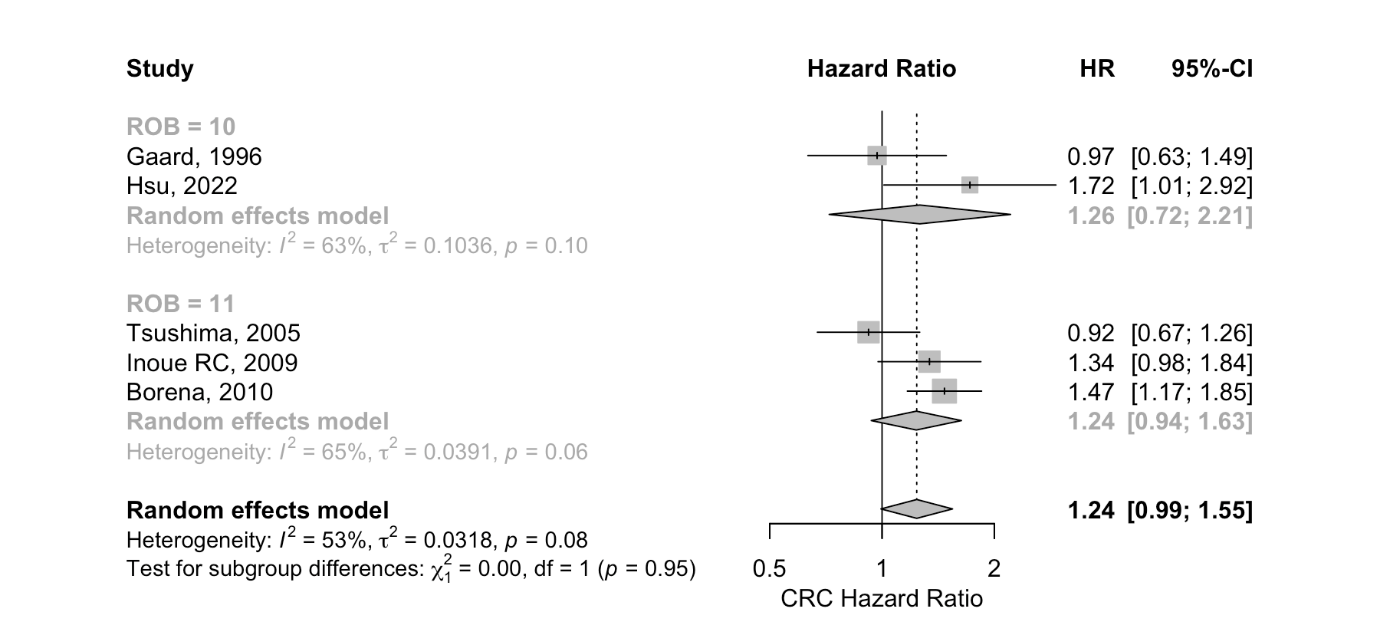


**Figure S29**. The forest plot for the risk of bias analysis of the association of TG and CRC.

HR, hazard ratio.


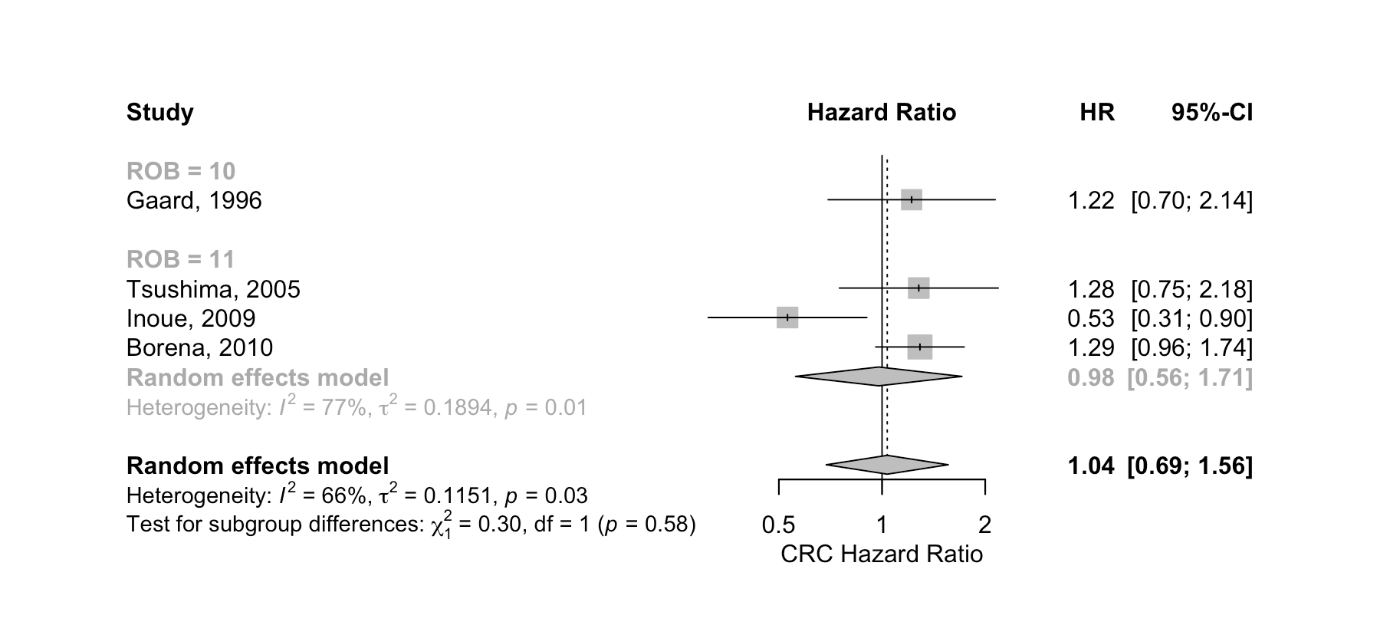


**Figure S30**. The forest plot for the risk of bias analysis of the association of TG and rectal cancer.

HR, hazard ratio.


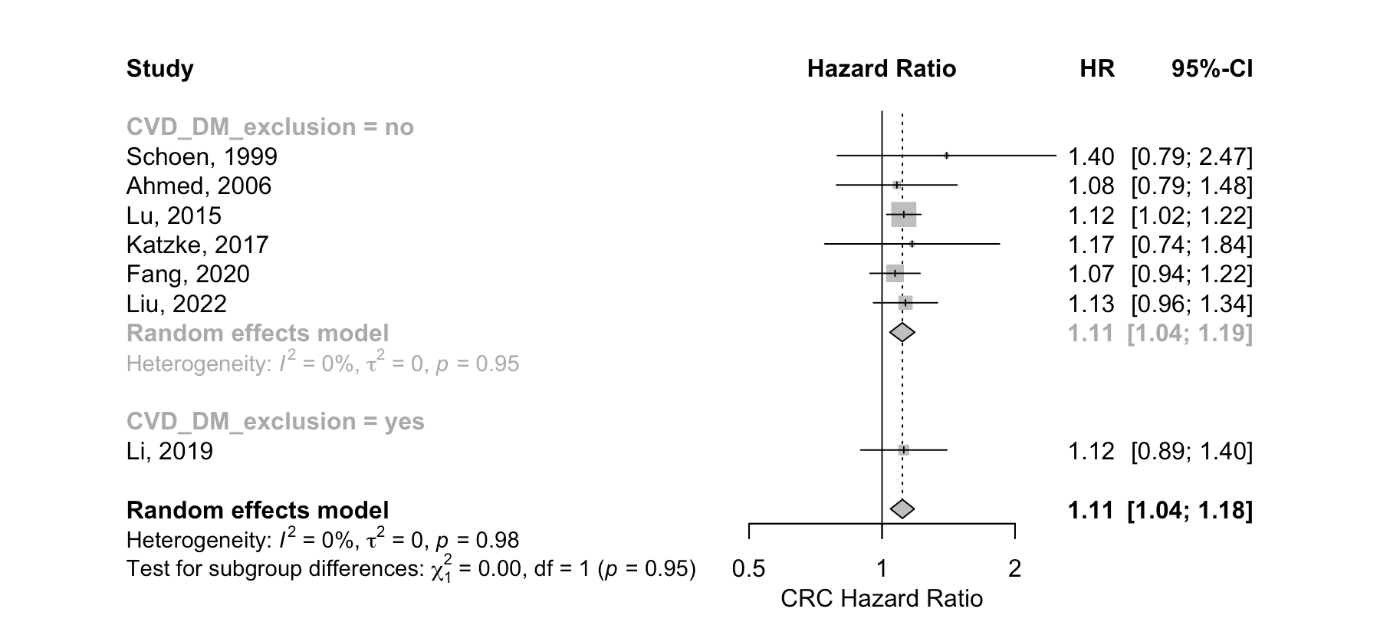
**Figure S31**. The forest plot for the association between serum TG

levels and the risk of colorectal cancer with and without exclusion of cases with cardiovascular diseases and diabetes mellitus.

HR, hazard ratio


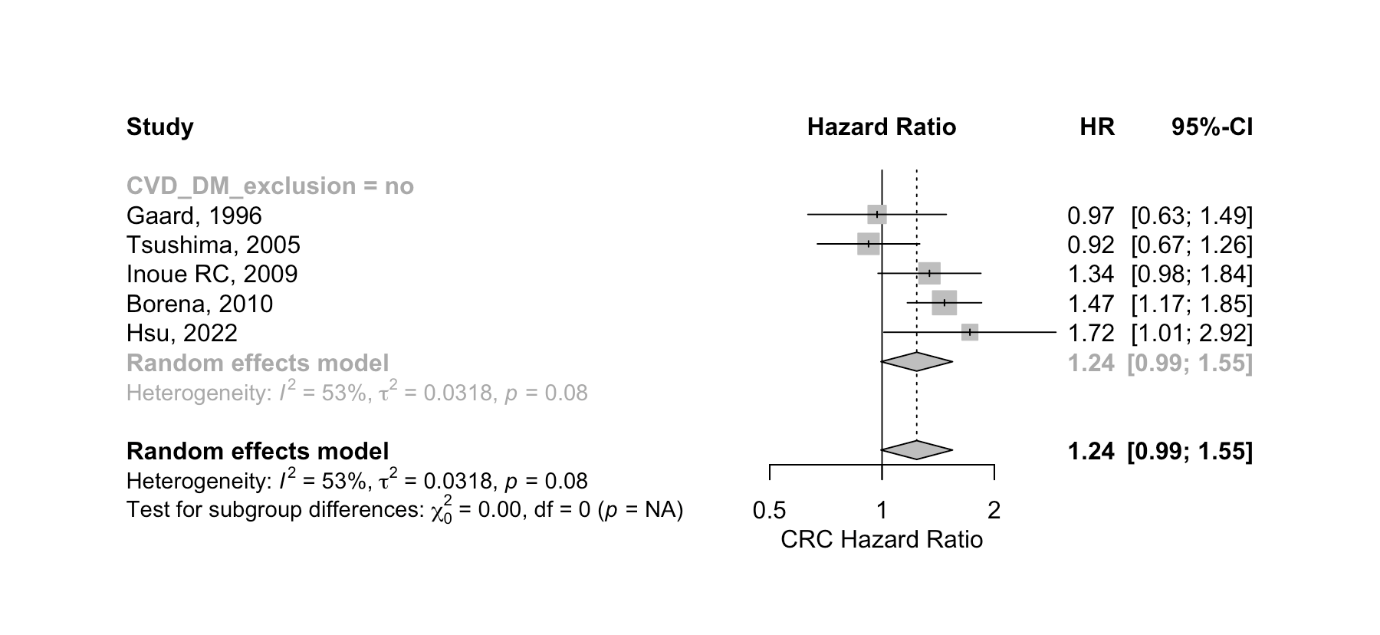
 **Figure S32**. The forest plot for the association between serum TG

levels and the risk of colon cancer with and without exclusion of cases with cardiovascular diseases and diabetes mellitus.

HR, hazard ratio


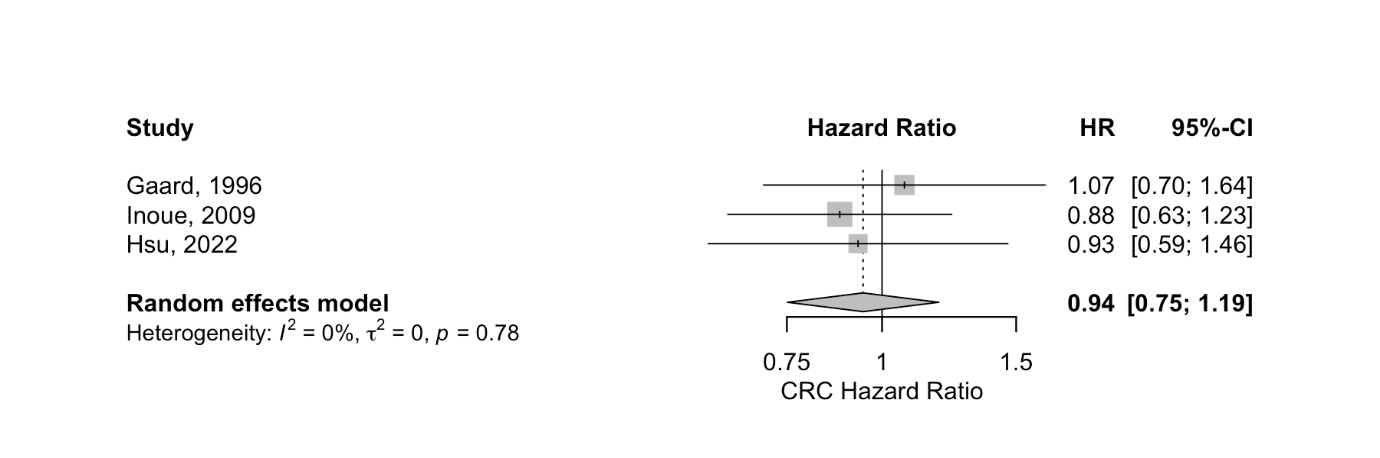
**Figure S33.** The forest plot for the association of HDL serum levels and risk of colon cancer.

HR, hazards ratio.


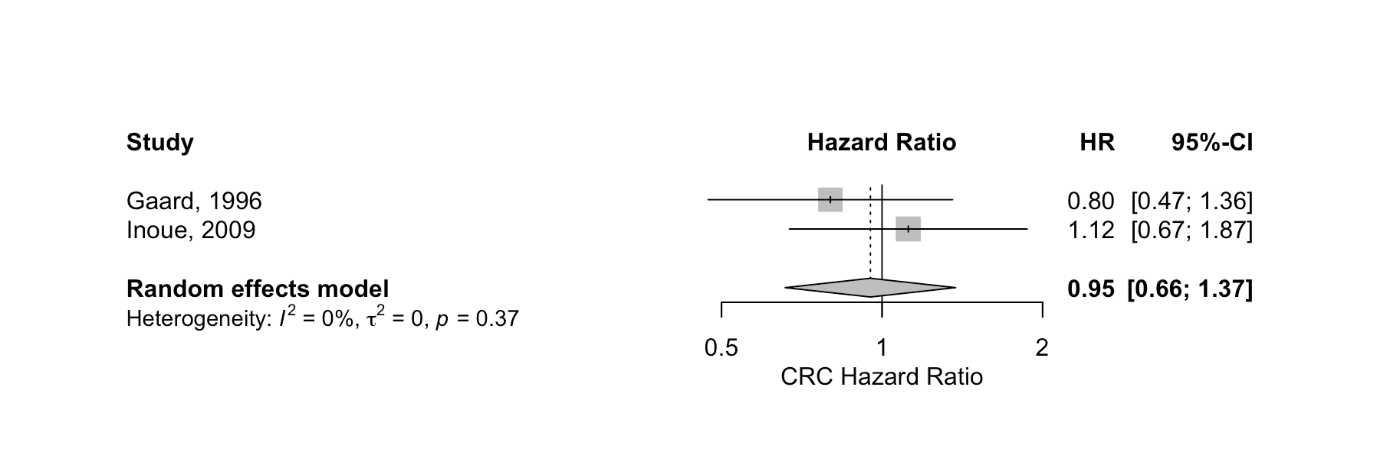


**Forest S34**. The forest plot for the association of HDL serum levels and risk of rectal cancer.

HR, hazards ratio.


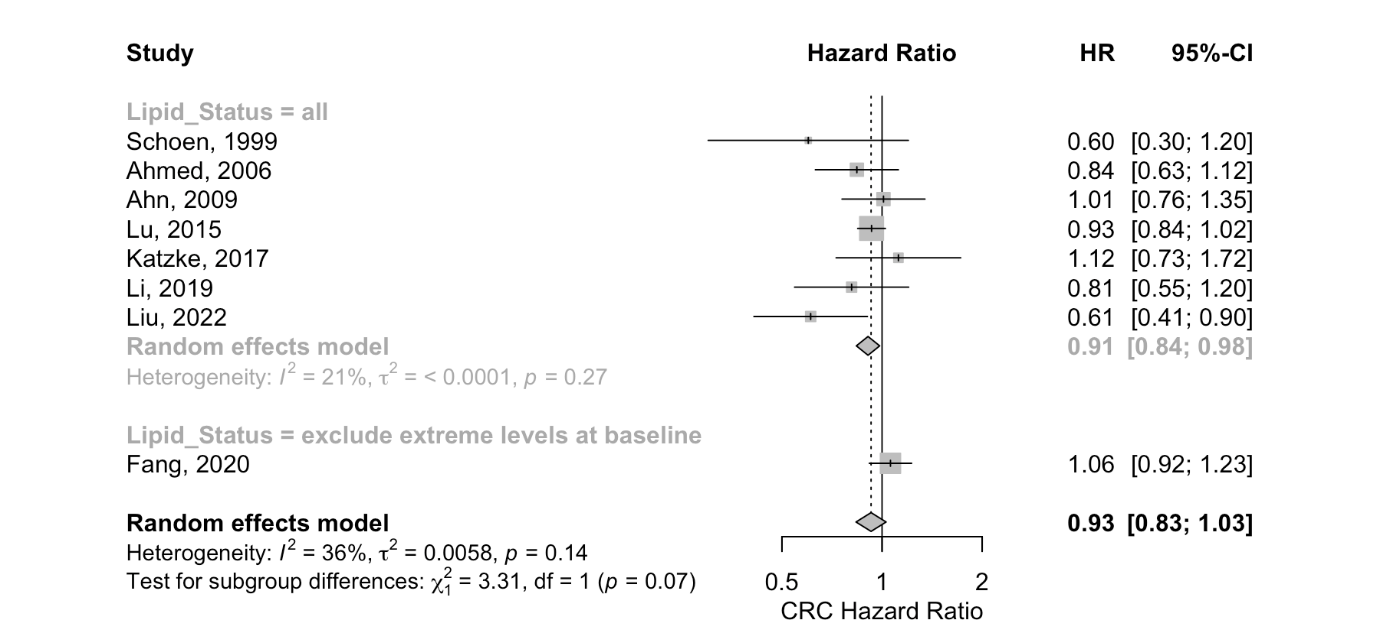


**Figure S35.** The forest plot for the association between serum HDL

levels and the risk of colorectal cancer with and without exclusion of extreme levels at baseline.

HR, hazard ratio.


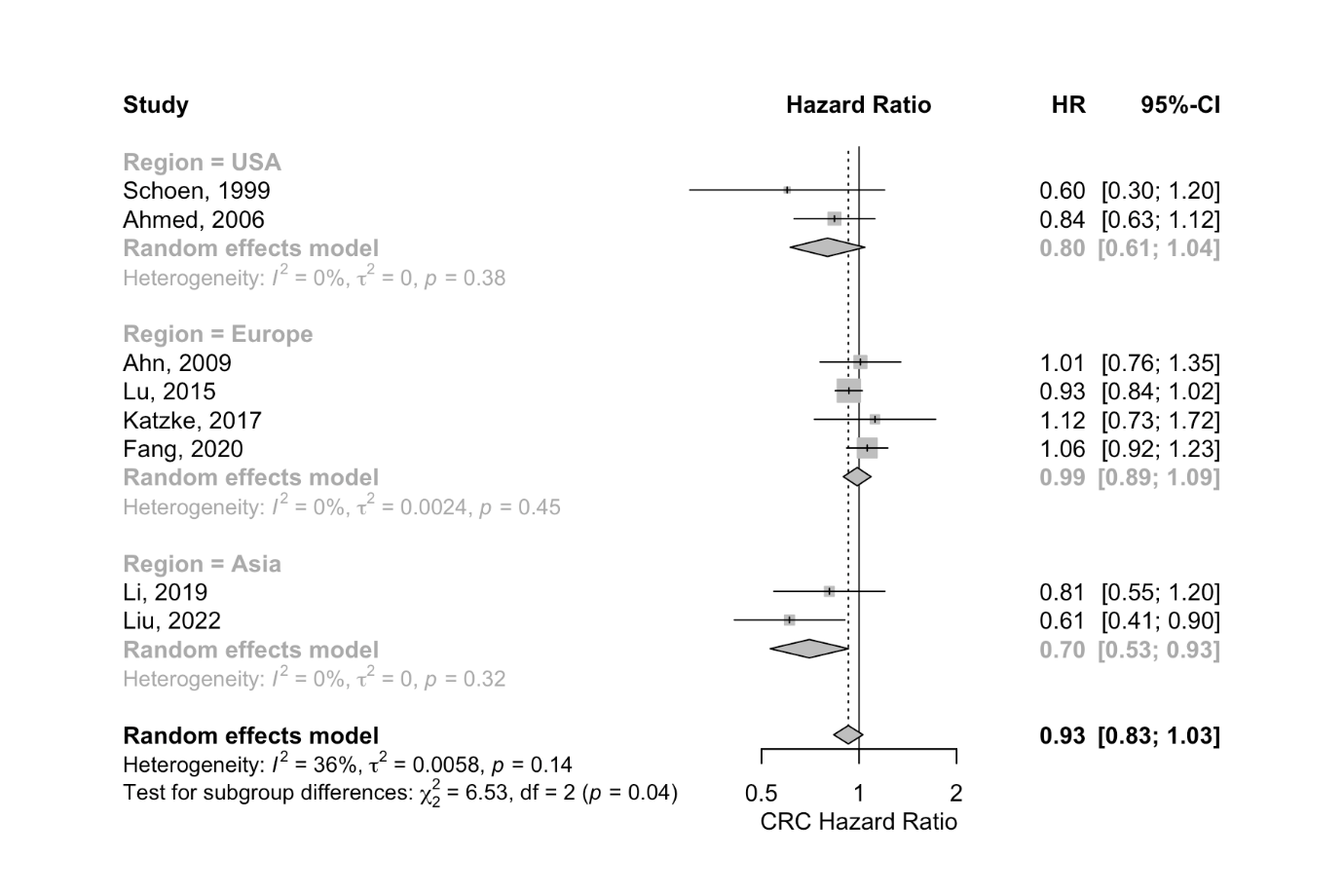
 **Figure S36.** The forest plot for the association between serum HDL

levels and the risk of colorectal cancer, categorized

by geographic region. HR, hazard ratio.


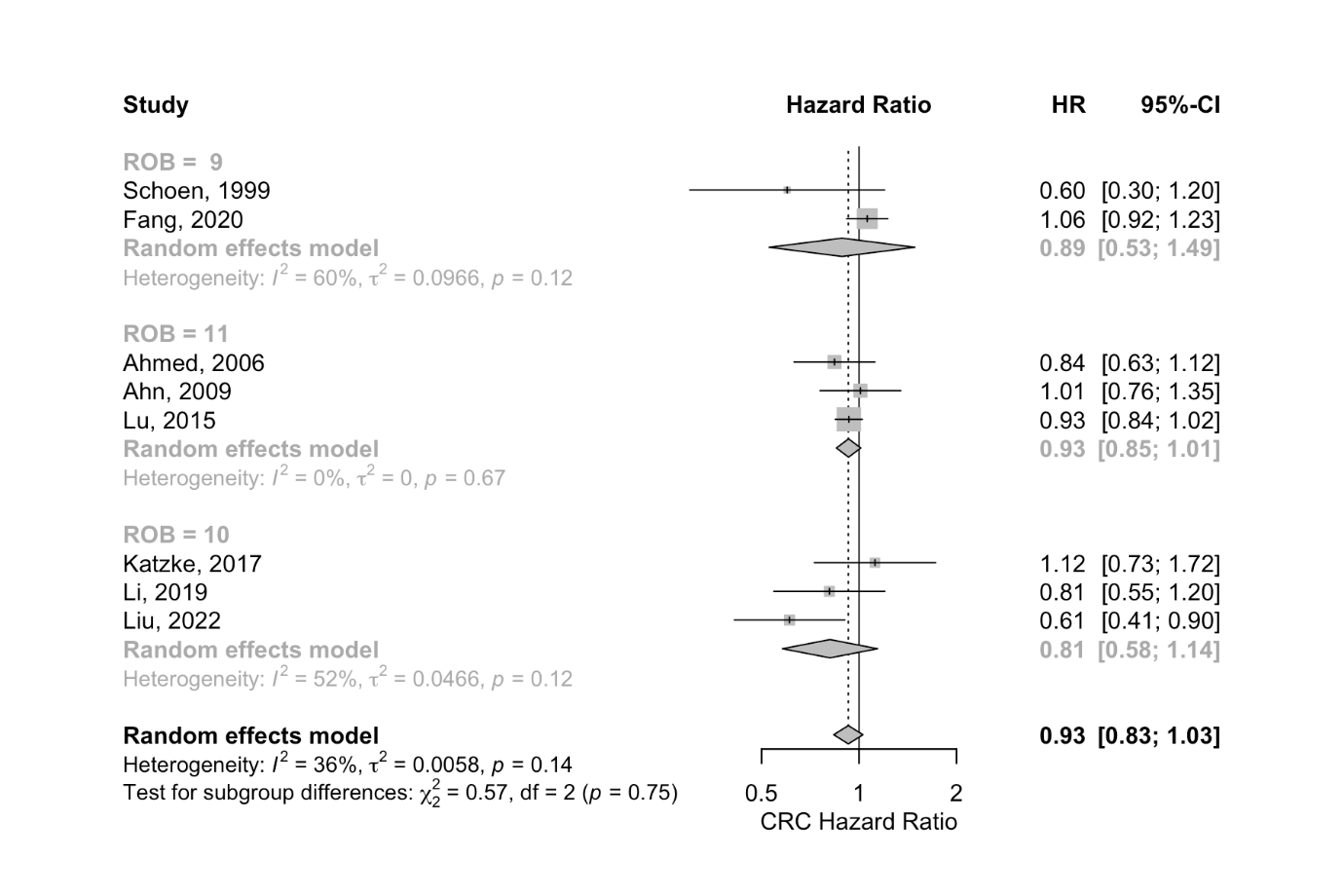
**Figure S37**. The forest plot for Risk of bias for association of HDL and colorectal cancer.

HR, hazard ratio


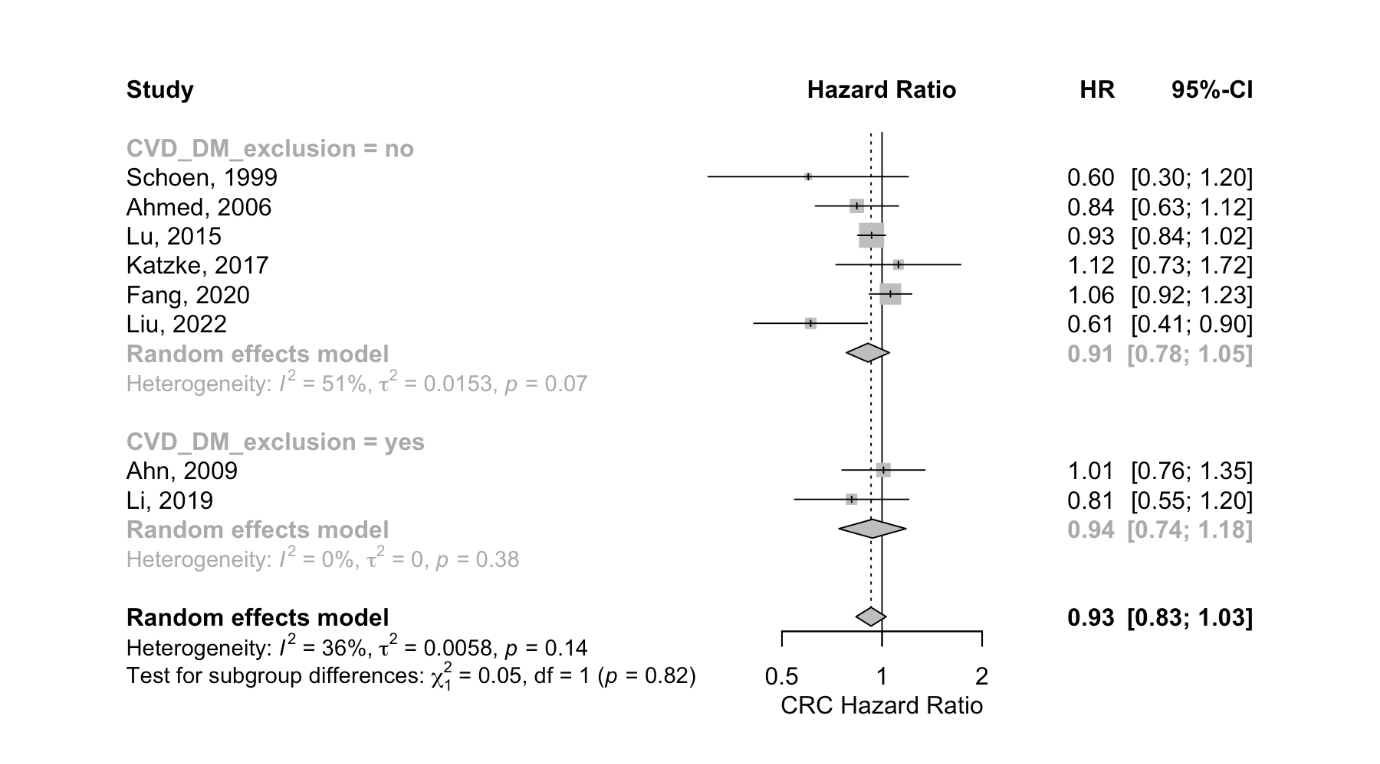


**Figure S38**. The forest plot for the association between serum HDL

levels and the risk of colorectal cancer with and without exclusion of cases with cardiovascular diseases and diabetes mellitus.

HR, hazard ratio


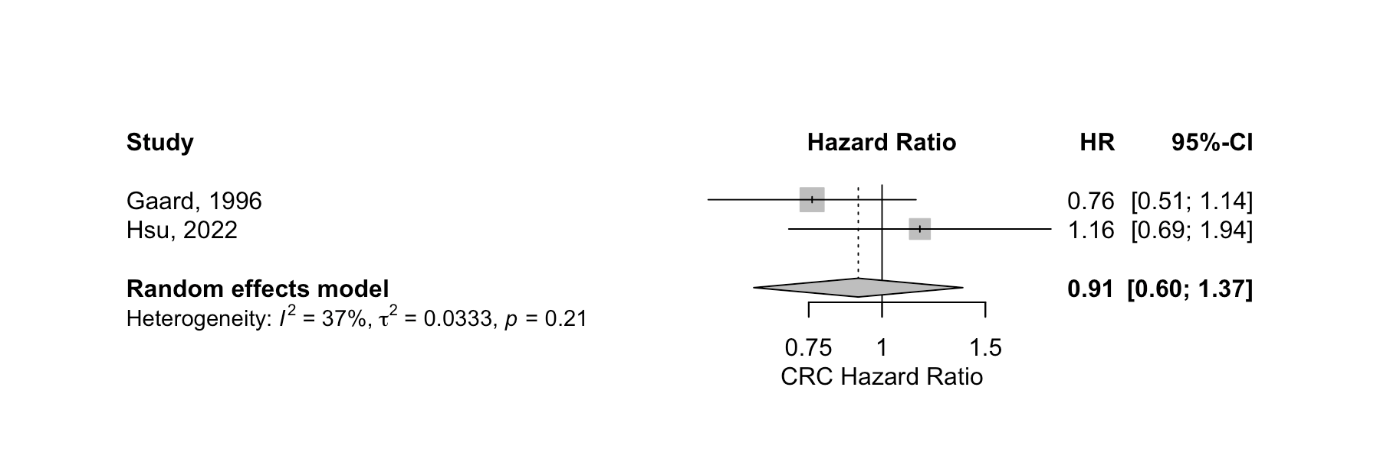


**Figure S39.** The forest plot for the association between serum LDL

levels and the risk of colon cancer.

HR, hazard ratio.


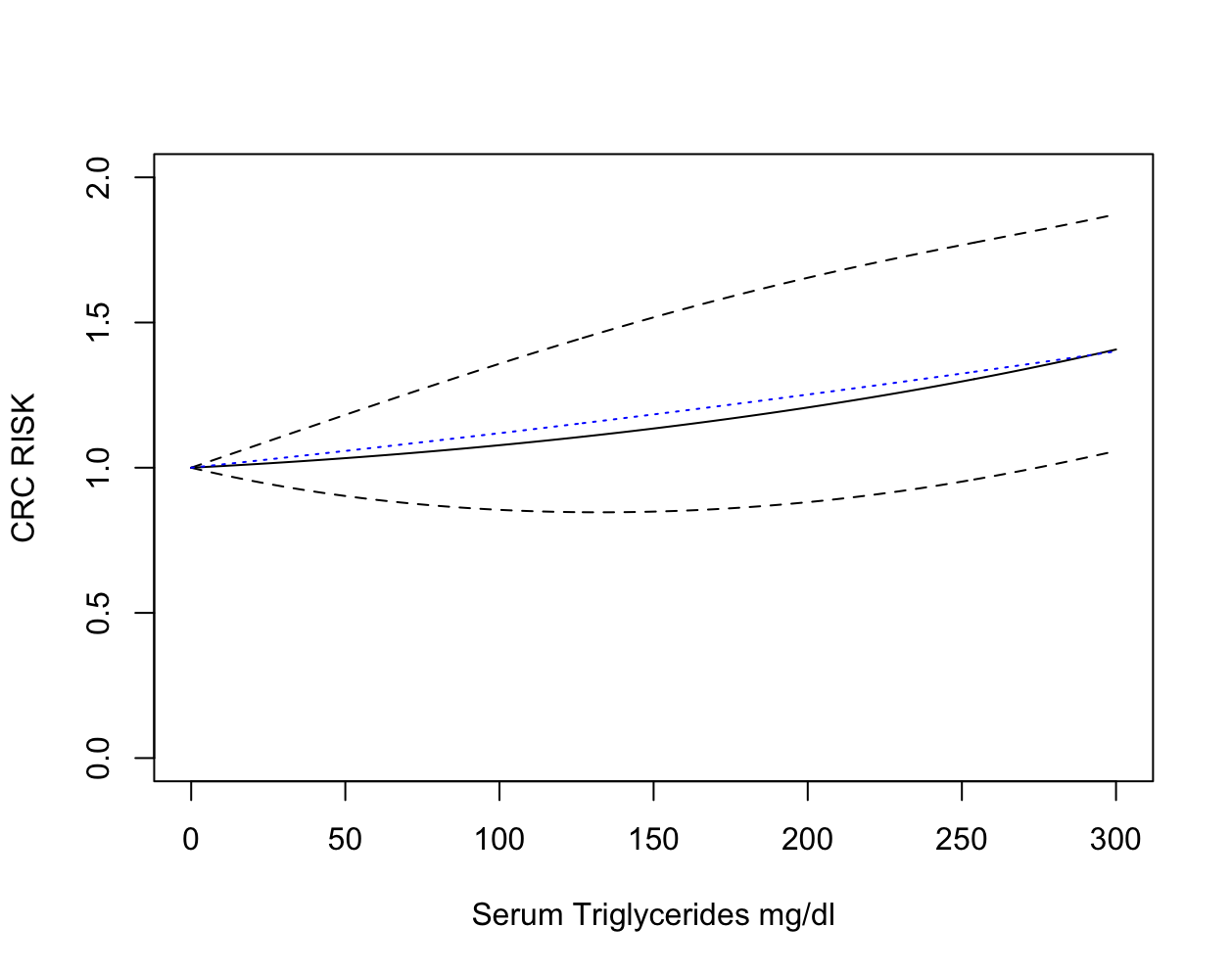


**Figure S40.** Dose-response analysis of the association between serum

triglyceride levels and the risk of colorectal cancer:

Quadratic and Linear Models.


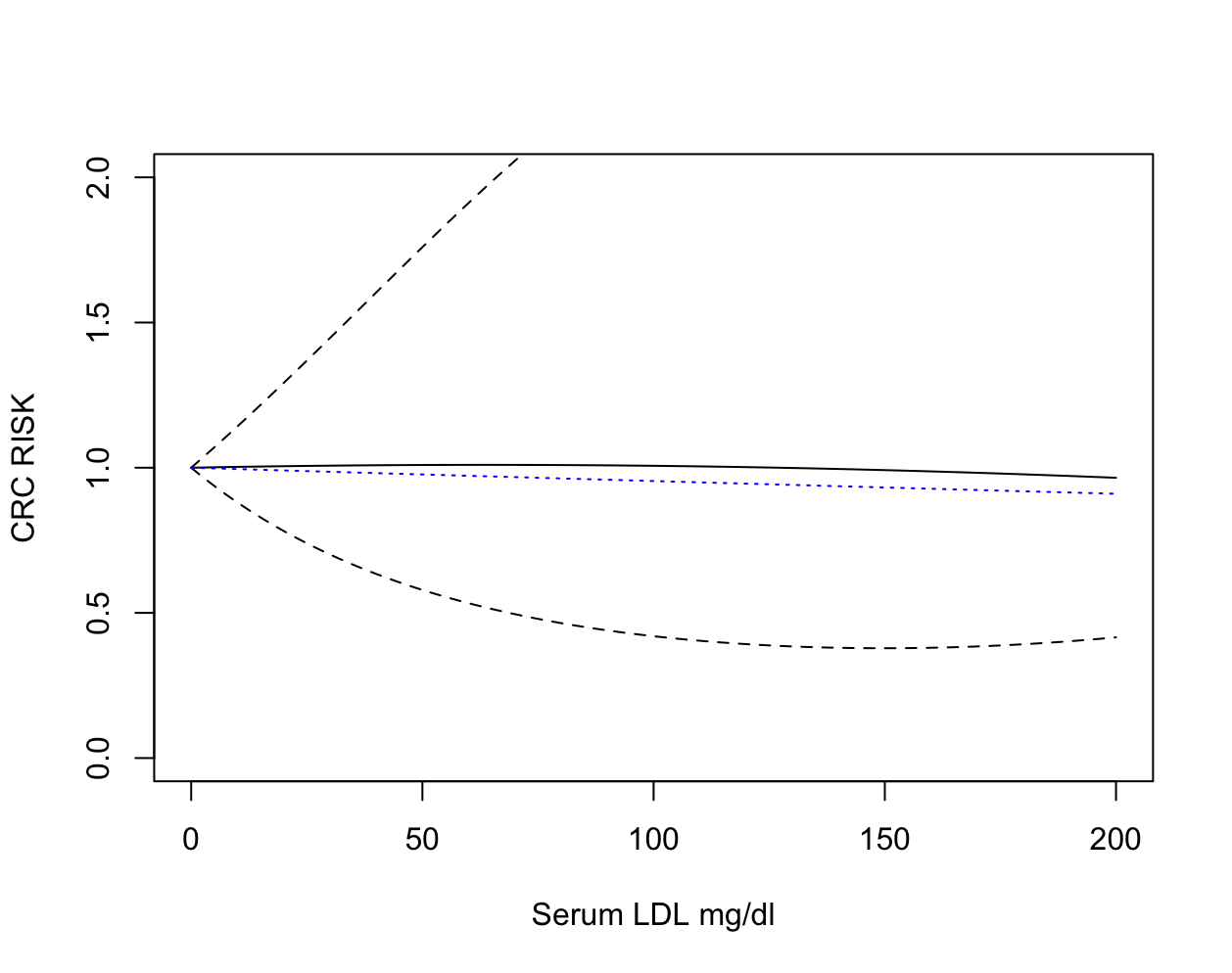


**Figure S41.** Dose-response analysis of the association between serum

LDL levels and the risk of colorectal cancer:

Quadratic and Linear Models.


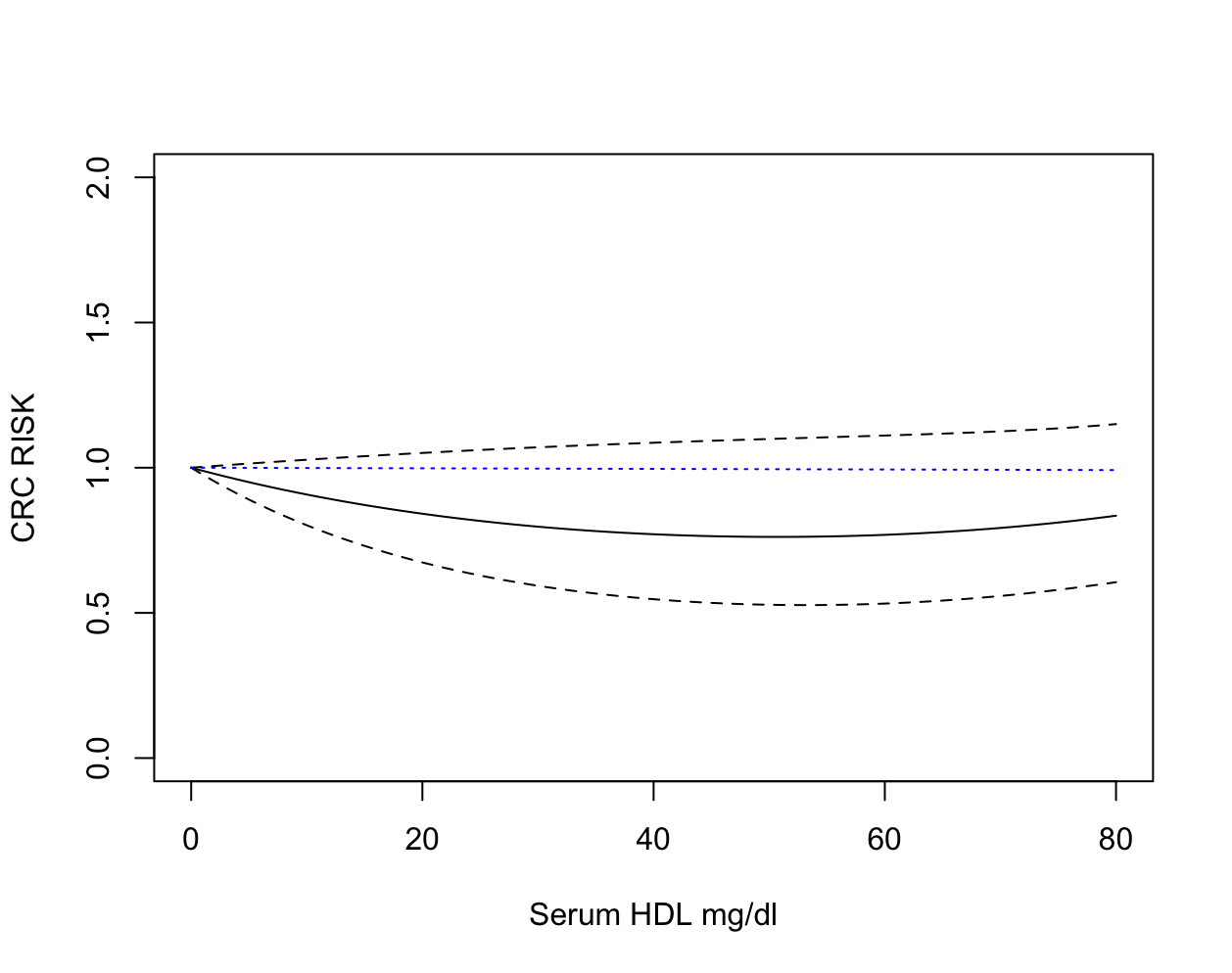


**Figure S42.** Dose-response analysis of the association between serum

HDL levels and the risk of colorectal cancer:

Quadratic and Linear Models.


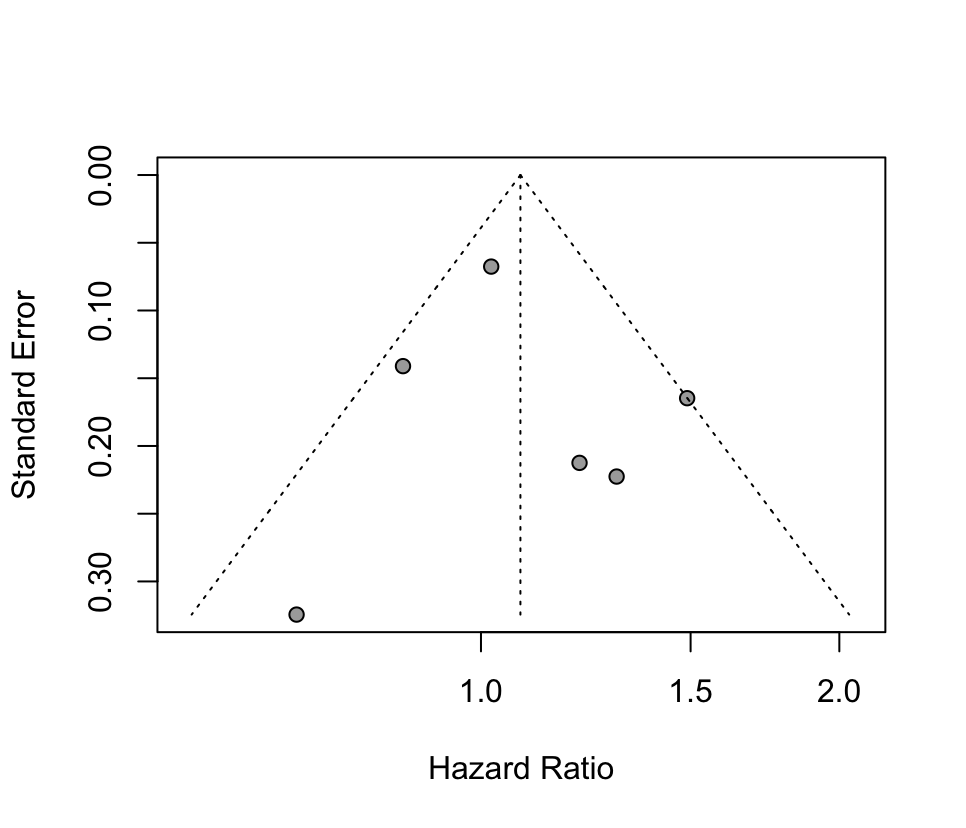


**Figure S43**. Funnel plot for serum cholesterol levels and CRC risk.


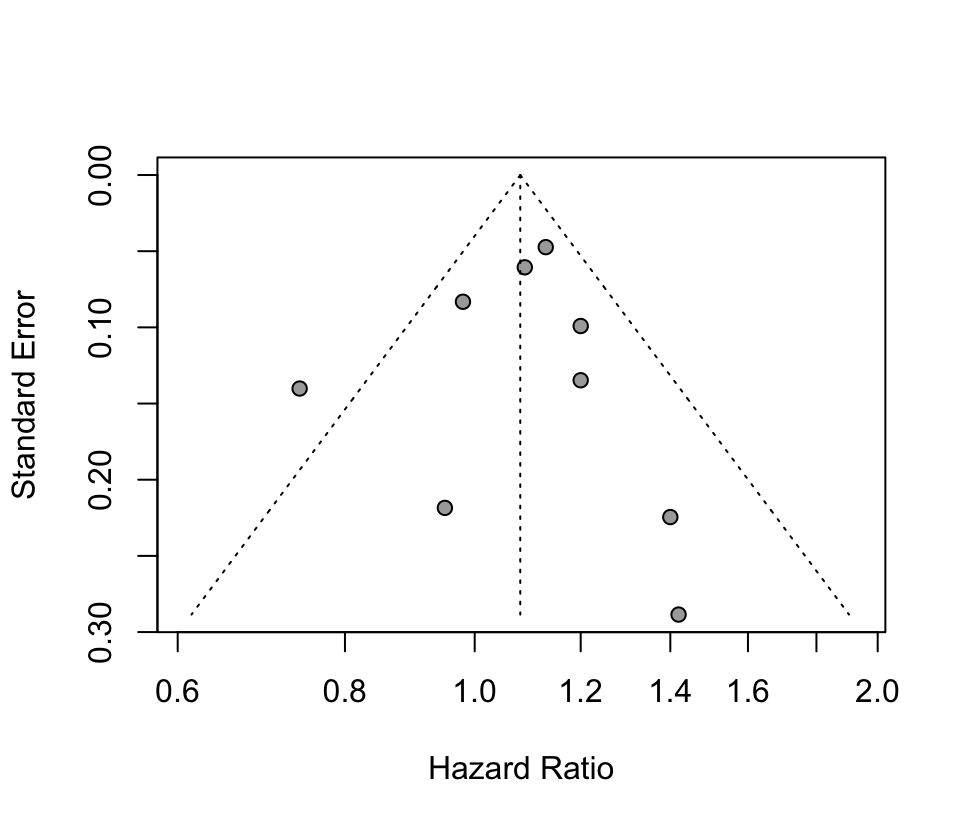
 **Figure S44**. Funnel plot for serum cholesterol levels and colon cancer risk.


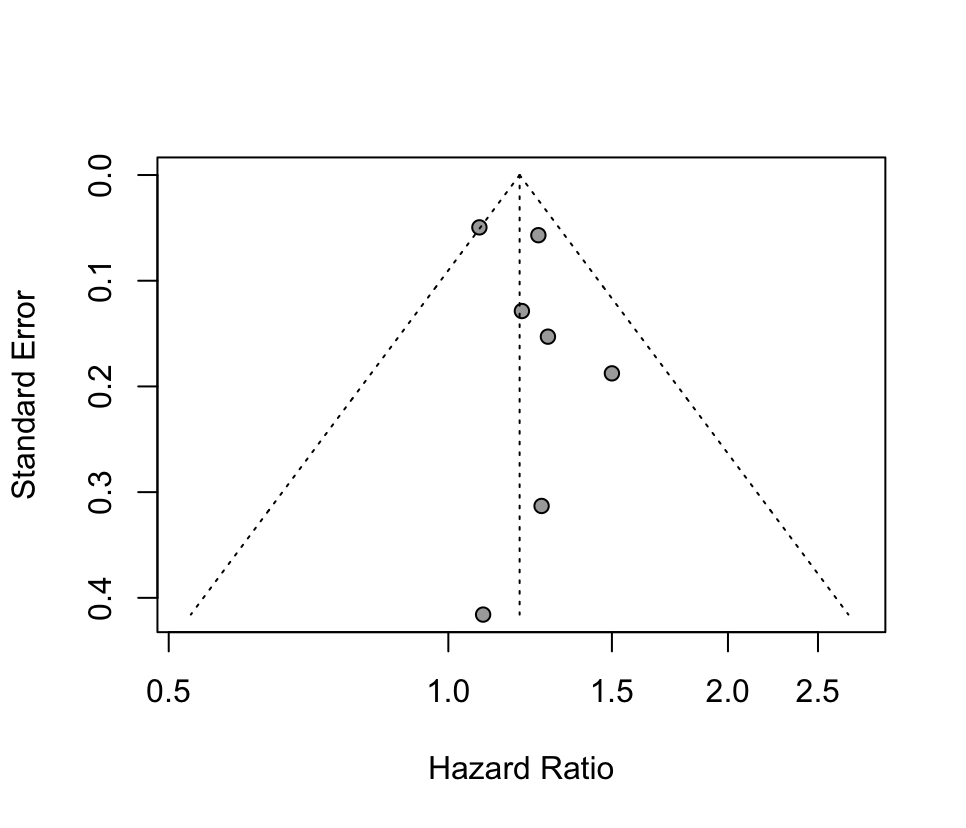


**Figure S45**. Funnel plot for serum cholesterol levels and rectal cancer risk.


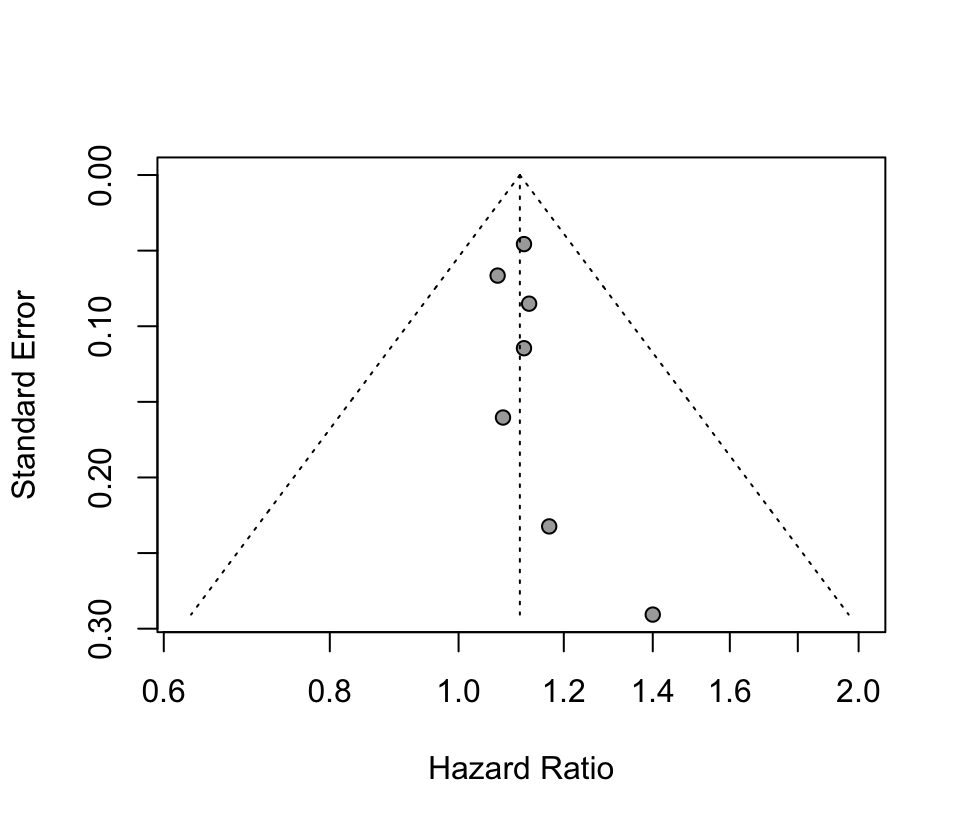


**Figure s46**. Funnel plot for serum TG levels and CRC risk.


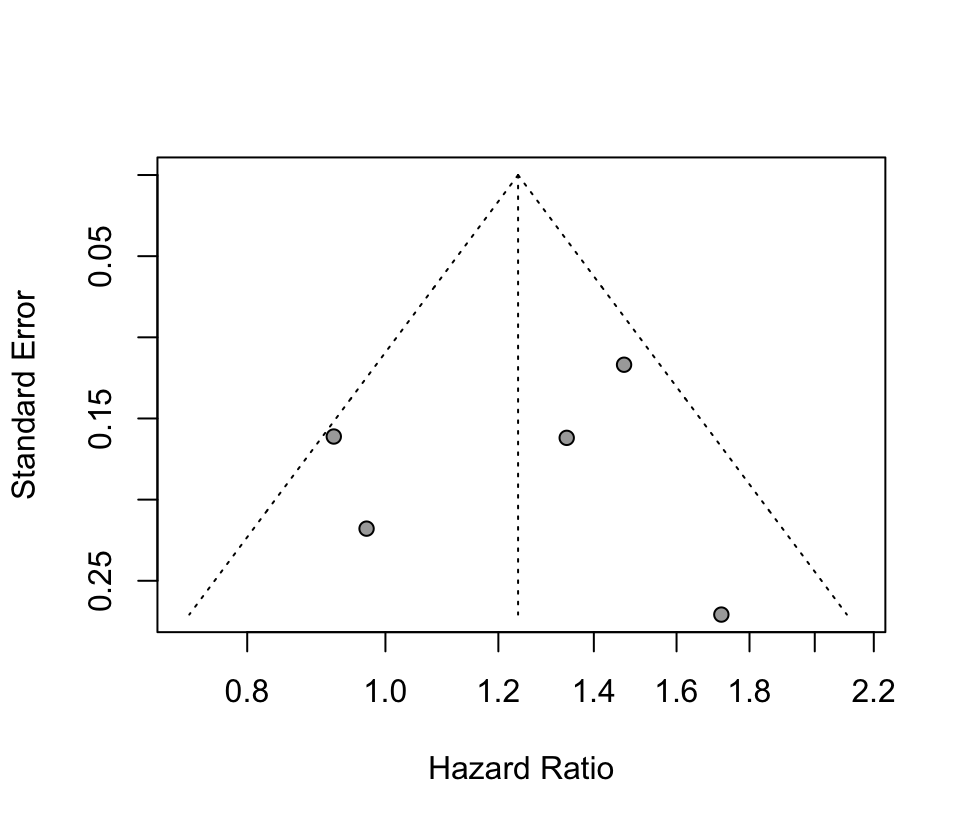


**Figure S47**. Funnel plot for serum TG levels and colon cancer risk.


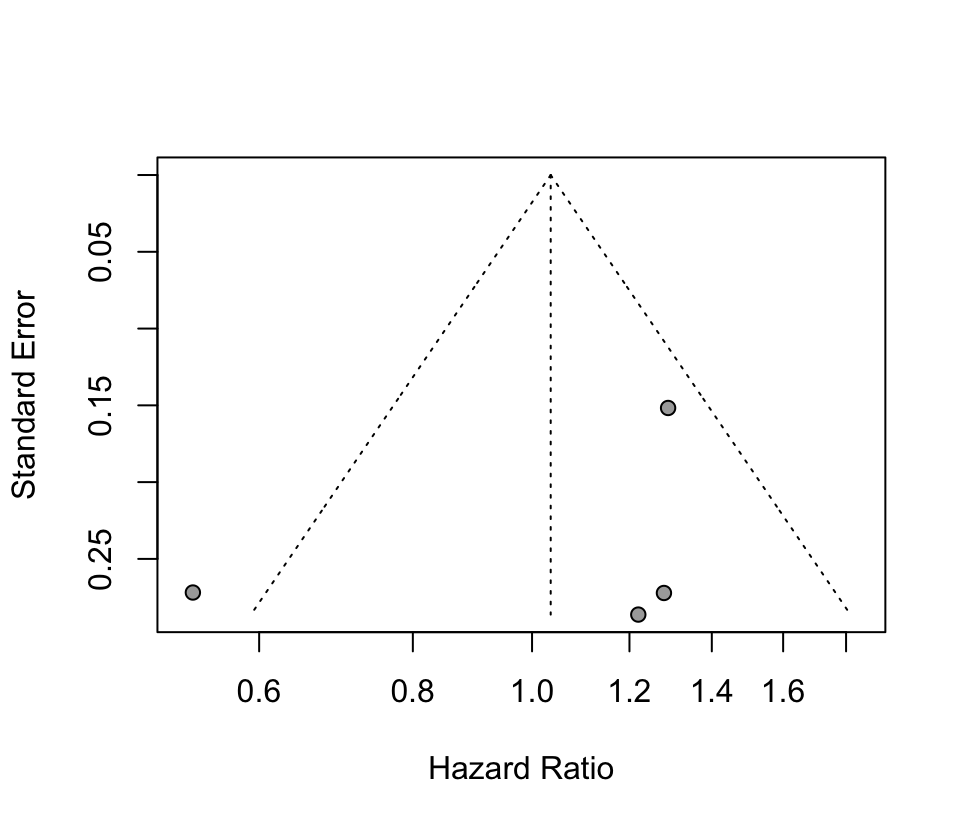
 **Figure S48**. Funnel plot for serum TG levels and rectal cancer risk.


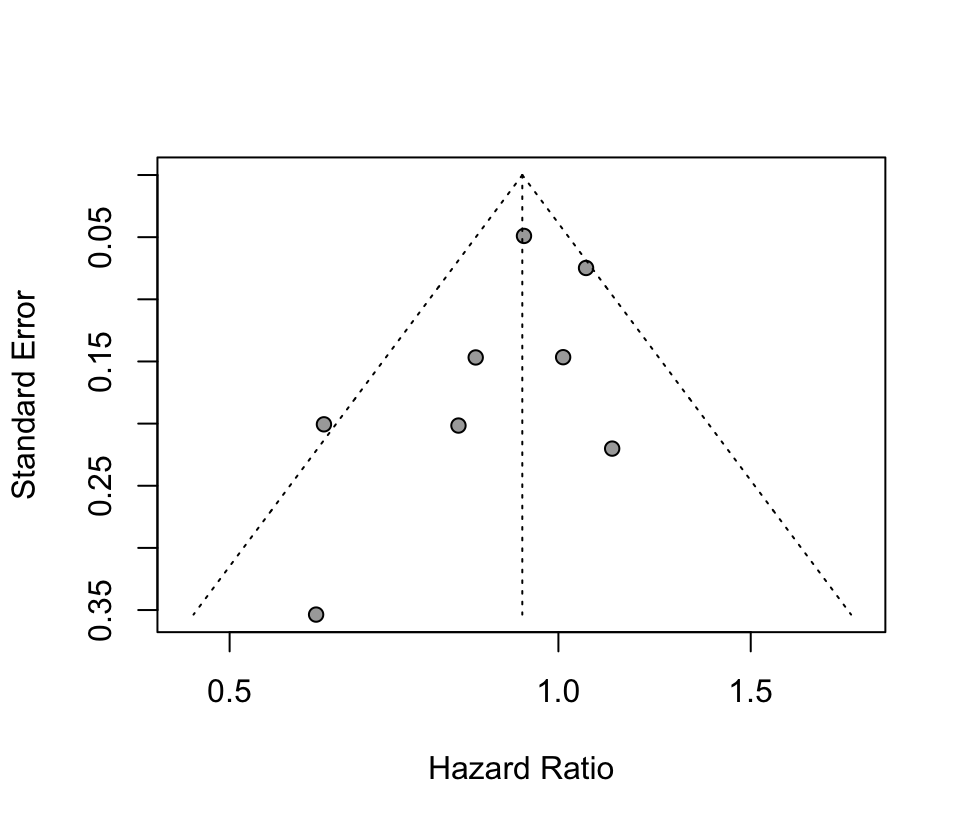


**Figure S49**. Funnel plot for serum HDL levels and CRC risk.


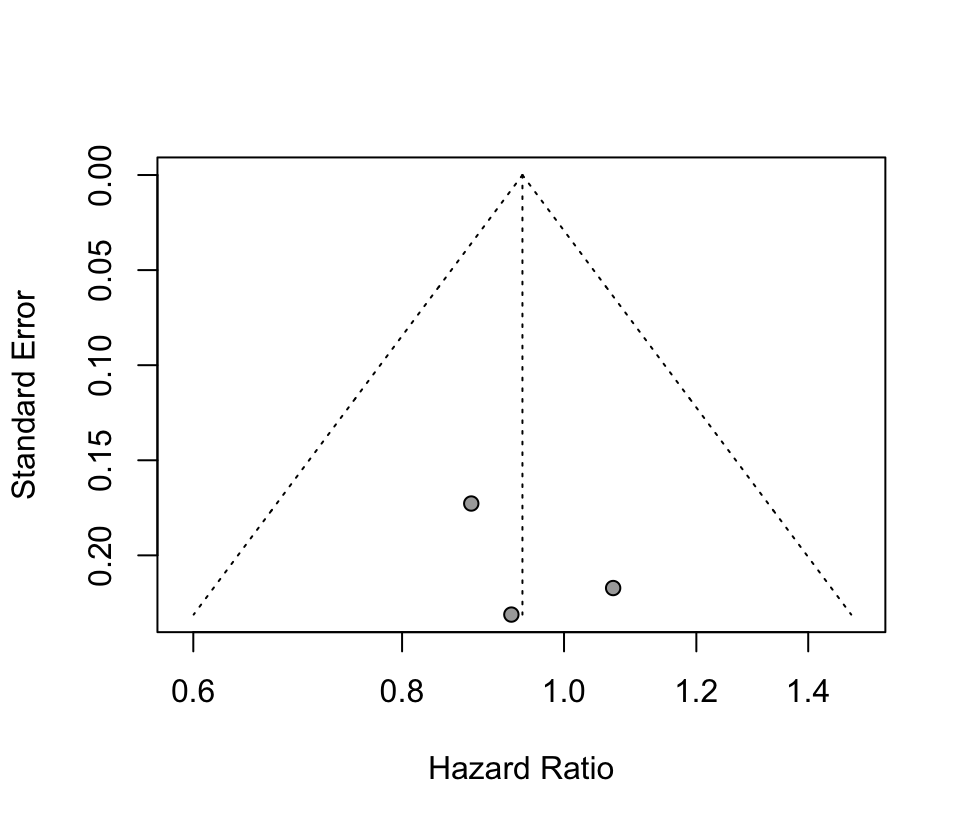


**Figure S50**. Funnel plot for serum HDL levels and colon cancer risk.


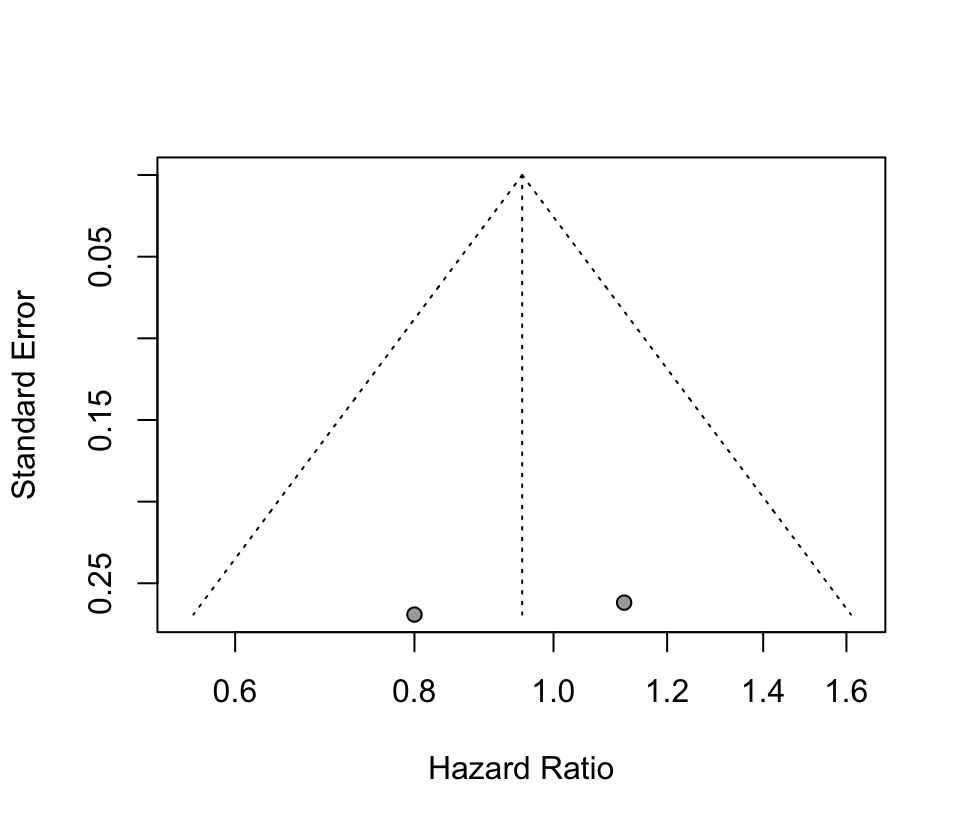


**Figure S51**. Funnel plot for serum HDL levels and rectal cancer risk.


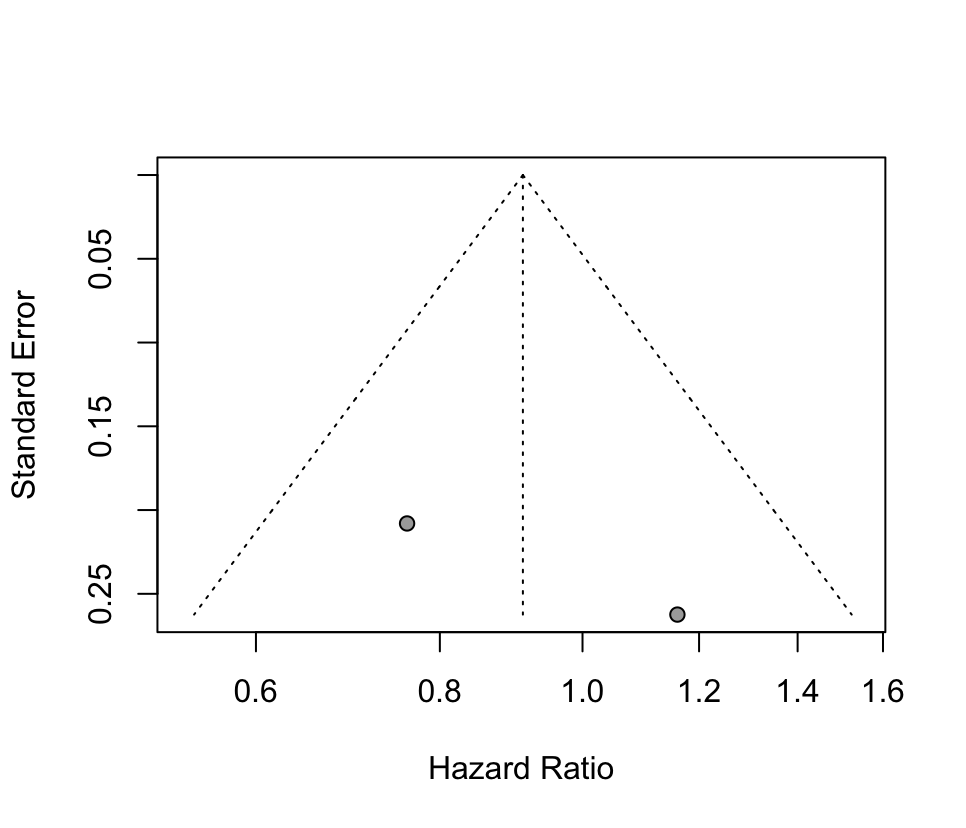


**Figure S52** Funnel plot for serum LDL levels and colon risk.

**Table S1.** Search details

| **Query** | | **Results**  **(21 September 2024)** |
| --- | --- | --- |
| **PubMed** | | |
| #1 | ("Colorectal Neoplasms"[MeSH] OR "colon cancer"[tiab]  OR "rectal cancer"[tiab] OR CRC[tiab] OR "colorectal adenoma"[tiab]  OR CRA[tiab] OR CRN[tiab] OR "large bowel cancer"[tiab]  OR "rectal carcinoma"[tiab] OR "colon carcinoma"[tiab]) | 299,871 |
| #2 | ("Lipids/blood"[MeSH] OR "Cholesterol"[MeSH] OR "Triglycerides/blood"  [MeSH] OR "Cholesterol, HDL"[MeSH] OR "Cholesterol, LDL"[MeSH] OR  "Cholesterol, VLDL"[MeSH] OR "Hyperlipidemias"[MeSH] OR "Dyslipidemias"[MeSH] OR "serum lipids"[tiab] OR "blood lipids"[tiab] OR "lipid profile"[tiab] OR "cholesterol"[tiab] OR "triglycerides"[tiab] OR "TG"[tiab] OR "Total cholesterol"[tiab] OR "TC"[tiab] OR "HDL-C"[tiab] OR "LDL-C"[tiab] OR "Lipid" [tiab] OR "Lipoprotein" [tiab] OR "Triglyceride" [tiab] OR "LDL" [tiab] OR "HDL" [tiab]) | 1,048,026 |
| #3 | #1 AND #2 | 5,372 |
| **Scopus** | | |
| #1 | (TITLE-ABS-KEY ("colorectal neoplasms") OR TITLE-ABS-KEY  ("colorectal cancer") OR TITLE-ABS-KEY("colon cancer") OR  TITLE-ABS-KEY("rectal cancer") OR TITLE-ABS-KEY(CRC) OR  TITLE-ABS-KEY("large bowel cancer") OR TITLE-ABS-KEY  ("colorectal carcinoma") OR TITLE-ABS-KEY("colorectal tumor") OR TITLE-ABS-KEY("colorectal adenoma") OR TITLE-ABS-KEY(CRA) OR TITLE-ABS-KEY(CRN)) | 399,264 |
| #2 | (TITLE-ABS-KEY ( "serum lipids" ) OR TITLE-ABS-KEY ( "blood lipids" )  OR TITLE-ABS-KEY ( "lipid profile" ) OR TITLE-ABS-KEY ( cholesterol )  OR TITLE-ABS-KEY ( "total cholesterol" ) OR TITLE-ABS-KEY ( tc ) OR  TITLE-ABS-KEY ( "triglycerides" ) OR TITLE-ABS-KEY ( tg ) OR TITLE-ABS-KEY ( "high-density lipoprotein cholesterol" ) OR TITLE-ABS-KEY ( hdl ) OR TITLE-ABS-KEY ( "HDL-C" ) OR TITLE-ABS-KEY ( "low-density lipoprotein cholesterol" ) OR TITLE-ABS-KEY ( ldl ) OR TITLE-ABS-KEY ( "LDL-C" ) OR TITLE-ABS-KEY ( "very low-density lipoprotein" ) OR TITLE-ABS-KEY ( vldl ) OR TITLE-ABS-KEY ( "hyperlipidemia" ) OR TITLE-ABS-KEY ( "dyslipidemia" ) OR TITLE-ABS-KEY ( lipid ) OR TITLE-ABS-KEY ( lipoprotein ) OR TITLE-ABS-KEY ( triglyceride ) OR TITLE-ABS-KEY ( LDL ) OR TITLE-ABS-KEY ( HDL )) | 2,055,520 |
| #3 | #1 AND #2 | 13,671 |
| **Web of Science** | | |
| #1 | TS= ("colorectal neoplasms" OR "colorectal cancer" OR "colon cancer" OR.  "rectal cancer" OR CRC OR "colorectal carcinoma" OR "colorectal adenoma"  OR "colorectal tumor" OR "large bowel cancer" OR "rectal neoplasm" OR  "colon neoplasm" OR CRA OR CRN) | 347,939 |
| #2 | TS= ("serum lipids" OR "blood lipids" OR "lipid profile" OR cholesterol OR  triglycerides OR TG OR "HDL-C" OR HDL OR "LDL-C" OR LDL OR "plasma lipids"  OR dyslipidemia OR hyperlipidemia OR "total cholesterol" OR TC OR "high-density lipoprotein cholesterol" OR "low-density lipoprotein cholesterol" OR "very low-density lipoprotein" OR VLDL OR Lipid OR Lipoprotein OR Triglyceride OR LDL OR HDL) | 1,480,615 |
| #3 | #1 AND #2 | 8,235 |
| **Total records** | | **27,278** |
| **Total records after removing duplicates** | | **16,857** |

**Table S2.** Specific serum Cholesterol levels and sssociated Odds Ratios

| Index | Dose (mg/dL) | Dose² | Odds Ratio | 95% CI (Lower) | 95% CI (Upper) |
| --- | --- | --- | --- | --- | --- |
| 1 | 80.0 | 6400.0 | 1.0 | 1.0 | 1.0 |
| 2 | 169.5 | 28730.25 | 1.007171 | 0.9485874 | 1.069373 |
| 3 | 189.5 | 35910.25 | 1.0135896 | 0.9465058 | 1.085428 |
| 4 | 219.5 | 48180.25 | 1.0266333 | 0.9490132 | 1.110602 |
| 5 | 259.5 | 67340.25 | 1.0506556 | 0.961709 | 1.147829 |
| 6 | 80.0 | 6400.0 | 1.0 | 1.0 | 1.0 |
| 7 | 169.5 | 28730.25 | 1.007171 | 0.9485874 | 1.069373 |
| 8 | 189.5 | 35910.25 | 1.0135896 | 0.9465058 | 1.085428 |
| 9 | 219.5 | 48180.25 | 1.0266333 | 0.9490132 | 1.110602 |
| 10 | 259.5 | 67340.25 | 1.0506556 | 0.961709 | 1.147829 |
| 11 | 80.0 | 6400.0 | 1.0 | 1.0 | 1.0 |
| 12 | 169.5 | 28730.25 | 1.007171 | 0.9485874 | 1.069373 |
| 13 | 189.5 | 35910.25 | 1.0135896 | 0.9465058 | 1.085428 |
| 14 | 219.5 | 48180.25 | 1.0266333 | 0.9490132 | 1.110602 |
| 15 | 259.5 | 67340.25 | 1.0506556 | 0.961709 | 1.147829 |
| 16 | 80.0 | 6400.0 | 1.0 | 1.0 | 1.0 |
| 17 | 169.5 | 28730.25 | 1.007171 | 0.9485874 | 1.069373 |
| 18 | 189.5 | 35910.25 | 1.0135896 | 0.9465058 | 1.085428 |
| 19 | 219.5 | 48180.25 | 1.0266333 | 0.9490132 | 1.110602 |
| 20 | 259.5 | 67340.25 | 1.0506556 | 0.961709 | 1.147829 |
| 21 | 80.0 | 6400.0 | 1.0 | 1.0 | 1.0 |
| 22 | 170.0 | 28900.0 | 1.0073096 | 0.9484969 | 1.069769 |
| 23 | 190.0 | 36100.0 | 1.0137732 | 0.9464935 | 1.085835 |
| 24 | 210.0 | 44100.0 | 1.0220524 | 0.9475207 | 1.102447 |
| 25 | 229.0 | 52441.0 | 1.031639 | 0.9511206 | 1.118974 |
| 26 | 250.0 | 62500.0 | 1.0442426 | 0.9578233 | 1.138459 |
| 27 | 80.0 | 6400.0 | 1.0 | 1.0 | 1.0 |
| 28 | 170.0 | 28900.0 | 1.0073096 | 0.9484969 | 1.069769 |
| 29 | 190.0 | 36100.0 | 1.0137732 | 0.9464935 | 1.085835 |
| 30 | 210.0 | 44100.0 | 1.0220524 | 0.9475207 | 1.102447 |
| 31 | 229.0 | 52441.0 | 1.031639 | 0.9511206 | 1.118974 |
| 32 | 250.0 | 62500.0 | 1.0442426 | 0.9578233 | 1.138459 |
| 33 | 80.0 | 6400.0 | 1.0 | 1.0 | 1.0 |
| 34 | 170.0 | 28900.0 | 1.0073096 | 0.9484969 | 1.069769 |
| 35 | 190.0 | 36100.0 | 1.0137732 | 0.9464935 | 1.085835 |
| 36 | 210.0 | 44100.0 | 1.0220524 | 0.9475207 | 1.102447 |
| 37 | 229.0 | 52441.0 | 1.031639 | 0.9511206 | 1.118974 |
| 38 | 250.0 | 62500.0 | 1.0442426 | 0.9578233 | 1.138459 |
| 39 | 101.9 | 10383.61 | 0.9985344 | 0.9805219 | 1.016878 |
| 40 | 216.0 | 46656.0 | 1.0248964 | 0.9483905 | 1.107574 |
| 41 | 238.0 | 56644.0 | 1.0367786 | 0.95366 | 1.127142 |
| 42 | 263.0 | 69169.0 | 1.0531323 | 0.9632637 | 1.151385 |
| 43 | 290.0 | 84100.0 | 1.0743549 | 0.9772119 | 1.181155 |
| 44 | 165.0 | 27225.0 | 1.0059732 | 0.9494928 | 1.065813 |
| 45 | 229.0 | 52441.0 | 1.031639 | 0.9511206 | 1.118974 |
| 46 | 250.0 | 62500.0 | 1.0442426 | 0.9578233 | 1.138459 |
| 47 | 80.0 | 6400.0 | 1.0 | 1.0 | 1.0 |
| 48 | 170.0 | 28900.0 | 1.0073096 | 0.9484969 | 1.069769 |
| 49 | 190.0 | 36100.0 | 1.0137732 | 0.9464935 | 1.085835 |
| 50 | 210.0 | 44100.0 | 1.0220524 | 0.9475207 | 1.102447 |
| 51 | 196.0 | 38416.0 | 1.0160645 | 0.9464921 | 1.090751 |
| 52 | 218.0 | 47524.0 | 1.0258819 | 0.9487361 | 1.109301 |
| 53 | 240.0 | 57600.0 | 1.0379738 | 0.9542935 | 1.128992 |
| 54 | 276.0 | 76176.0 | 1.0628775 | 0.9695744 | 1.165159 |
| 55 | 162.2 | 26308.84 | 1.0052733 | 0.9501393 | 1.063607 |
| 56 | 255.5 | 65280.25 | 1.0479005 | 0.9600121 | 1.143835 |
| 57 | 478.8 | 229249.44 | 1.3494733 | 1.0830557 | 1.681426 |
| 58 | 126.0 | 15876.0 | 0.9993272 | 0.9644685 | 1.035446 |
| 59 | 193.0 | 37249.0 | 1.0148984 | 0.946459 | 1.088287 |
| 60 | 220.0 | 48400.0 | 1.0268861 | 0.949109 | 1.111037 |
| 61 | 354.0 | 125316.0 | 1.1407212 | 1.0183401 | 1.27781 |
| 62 | 101.0 | 10201.0 | 0.9985536 | 0.9812282 | 1.016185 |
| 63 | 217.0 | 47089.0 | 1.0253868 | 0.9485598 | 1.108436 |
| 64 | 248.0 | 61504.0 | 1.0429496 | 0.9570701 | 1.136535 |
| 65 | 281.0 | 78961.0 | 1.0668582 | 0.9722096 | 1.170721 |
| 66 | 101.0 | 10201.0 | 0.9985536 | 0.9812282 | 1.016185 |
| 67 | 217.0 | 47089.0 | 1.0253868 | 0.9485598 | 1.108436 |
| 68 | 248.0 | 61504.0 | 1.0429496 | 0.9570701 | 1.136535 |
| 69 | 281.0 | 78961.0 | 1.0668582 | 0.9722096 | 1.170721 |
| 70 | 102.0 | 10404.0 | 0.9985325 | 0.9804439 | 1.016955 |
| 71 | 220.0 | 48400.0 | 1.0268861 | 0.949109 | 1.111037 |
| 72 | 251.0 | 63001.0 | 1.0448965 | 0.9582086 | 1.139427 |
| 73 | 283.0 | 80089.0 | 1.0684871 | 0.9732934 | 1.172991 |
| 74 | 102.0 | 10404.0 | 0.9985325 | 0.9804439 | 1.016955 |
| 75 | 220.0 | 48400.0 | 1.0268861 | 0.949109 | 1.111037 |
| 76 | 251.0 | 63001.0 | 1.0448965 | 0.9582086 | 1.139427 |
| 77 | 283.0 | 80089.0 | 1.0684871 | 0.9732934 | 1.172991 |
| 78 | 100.0 | 10000.0 | 0.9985791 | 0.9820224 | 1.015415 |
| 79 | 207.0 | 42849.0 | 1.0206931 | 0.947181 | 1.099911 |
| 80 | 227.0 | 51529.0 | 1.0305496 | 0.9506271 | 1.117191 |
| 81 | 253.0 | 64009.0 | 1.0462192 | 0.9589961 | 1.141375 |
| 82 | 112.0 | 12544.0 | 0.9985599 | 0.9731299 | 1.024654 |
| 83 | 237.0 | 56169.0 | 1.0361883 | 0.9533525 | 1.126222 |
| 84 | 262.0 | 68644.0 | 1.0524183 | 0.962813 | 1.150363 |
| 85 | 289.0 | 83521.0 | 1.0735006 | 0.9766407 | 1.179967 |
| 86 | 112.0 | 12544.0 | 0.9985599 | 0.9731299 | 1.024654 |
| 87 | 237.0 | 56169.0 | 1.0361883 | 0.9533525 | 1.126222 |
| 88 | 262.0 | 68644.0 | 1.0524183 | 0.962813 | 1.150363 |
| 89 | 289.0 | 83521.0 | 1.0735006 | 0.9766407 | 1.179967 |
| 90 | 112.0 | 12544.0 | 0.9985599 | 0.9731299 | 1.024654 |
| 91 | 237.0 | 56169.0 | 1.0361883 | 0.9533525 | 1.126222 |
| 92 | 262.0 | 68644.0 | 1.0524183 | 0.962813 | 1.150363 |
| 93 | 289.0 | 83521.0 | 1.0735006 | 0.9766407 | 1.179967 |
| 94 | 112.0 | 12544.0 | 0.9985599 | 0.9731299 | 1.024654 |
| 95 | 237.0 | 56169.0 | 1.0361883 | 0.9533525 | 1.126222 |
| 96 | 262.0 | 68644.0 | 1.0524183 | 0.962813 | 1.150363 |
| 97 | 289.0 | 83521.0 | 1.0735006 | 0.9766407 | 1.179967 |

**Table S3.** Specific serum Triglyceride levels and associated Odds Ratios

| Index | Dose (mg/dL) | Dose² | Odds Ratio | 95% CI (Lower) | 95% CI (Upper) |
| --- | --- | --- | --- | --- | --- |
| 1 | 70 | 4900 | 1.0 | 1.0 | 1.0 |
| 2 | 100 | 10000 | 1.0268616 | 0.9731841 | 1.0835 |
| 3 | 133 | 17689 | 1.0615618 | 0.9626333 | 1.170657 |
| 4 | 180 | 32400 | 1.1212528 | 0.9786551 | 1.284628 |
| 5 | 286 | 81796 | 1.3094701 | 1.1077748 | 1.547889 |
| 6 | 70 | 4900 | 1.0 | 1.0 | 1.0 |
| 7 | 100 | 10000 | 1.0268616 | 0.9731841 | 1.0835 |
| 8 | 133 | 17689 | 1.0615618 | 0.9626333 | 1.170657 |
| 9 | 180 | 32400 | 1.1212528 | 0.9786551 | 1.284628 |
| 10 | 286 | 81796 | 1.3094701 | 1.1077748 | 1.547889 |
| 11 | 58 | 3364 | 0.9904314 | 0.965767 | 1.015726 |
| 12 | 77 | 5929 | 1.0058866 | 0.992185 | 1.019777 |
| 13 | 99 | 9801 | 1.0258963 | 0.9738044 | 1.080775 |
| 14 | 130 | 16900 | 1.0581726 | 0.9628183 | 1.16297 |
| 15 | 197 | 38809 | 1.1461023 | 0.9926434 | 1.323285 |
| 16 | 58 | 3364 | 0.9904314 | 0.965767 | 1.015726 |
| 17 | 77 | 5929 | 1.0058866 | 0.992185 | 1.019777 |
| 18 | 99 | 9801 | 1.0258963 | 0.9738044 | 1.080775 |
| 19 | 130 | 16900 | 1.0581726 | 0.9628183 | 1.16297 |
| 20 | 197 | 38809 | 1.1461023 | 0.9926434 | 1.323285 |
| 21 | 69 | 4761 | 0.9991775 | 0.9971623 | 1.001197 |
| 22 | 99 | 9801 | 1.0258963 | 0.9738044 | 1.080775 |
| 23 | 131 | 17161 | 1.059297 | 0.9627399 | 1.165538 |
| 24 | 174 | 30276 | 1.1129127 | 0.9747062 | 1.270716 |
| 25 | 263 | 69169 | 1.2613839 | 1.0762188 | 1.478407 |
| 26 | 35 | 1225 | 0.9738826 | 0.899324 | 1.054622 |
| 27 | 84 | 7056 | 1.0120023 | 0.9853367 | 1.039389 |
| 28 | 118 | 13924 | 1.0450925 | 0.9650828 | 1.131735 |
| 29 | 159 | 25281 | 1.0930077 | 0.9672093 | 1.235168 |
| 30 | 35 | 1225 | 0.9738826 | 0.899324 | 1.054622 |
| 31 | 84 | 7056 | 1.0120023 | 0.9853367 | 1.039389 |
| 32 | 118 | 13924 | 1.0450925 | 0.9650828 | 1.131735 |
| 33 | 159 | 25281 | 1.0930077 | 0.9672093 | 1.235168 |
| 34 | 45 | 2025 | 0.9807935 | 0.9283136 | 1.03624 |
| 35 | 110 | 12100 | 1.0367876 | 0.9679738 | 1.110493 |
| 36 | 157 | 24649 | 1.0904534 | 0.9664724 | 1.230339 |
| 37 | 216 | 46656 | 1.1761037 | 1.0127683 | 1.365781 |
| 38 | 45 | 2025 | 0.9807935 | 0.9283136 | 1.03624 |
| 39 | 110 | 12100 | 1.0367876 | 0.9679738 | 1.110493 |
| 40 | 157 | 24649 | 1.0904534 | 0.9664724 | 1.230339 |
| 41 | 216 | 46656 | 1.1761037 | 1.0127683 | 1.365781 |

**Table S4.** Specific serum HDL levels and associated Odds Ratios

| Index | Dose (mg/dL) | Dose² | Odds Ratio | 95% CI (Lower) | 95% CI (Upper) |
| --- | --- | --- | --- | --- | --- |
| 1 | 18.1 | 327.61 | 1.0 | 1.0 | 1.0 |
| 2 | 38.9 | 1513.21 | 0.9068504 | 0.7923801 | 1.037857 |
| 3 | 44.4 | 1971.36 | 0.8973817 | 0.7695358 | 1.046467 |
| 4 | 51.2 | 2621.44 | 0.8937131 | 0.7553374 | 1.057439 |
| 5 | 59.35 | 3522.423 | 0.9009159 | 0.7561953 | 1.073333 |
| 6 | 38.0 | 1444.0 | 0.908965 | 0.797178 | 1.036428 |
| 7 | 47.0 | 2209.0 | 0.8949389 | 0.7623709 | 1.050559 |
| 8 | 53.0 | 2809.0 | 0.894213 | 0.7539475 | 1.060574 |
| 9 | 61.0 | 3721.0 | 0.9039318 | 0.7584616 | 1.077303 |
| 10 | 75.0 | 5625.0 | 0.9518179 | 0.7975775 | 1.135886 |
| 11 | 22.0 | 484.0 | 0.9749867 | 0.9429911 | 1.008068 |
| 12 | 50.0 | 2500.0 | 0.8937215 | 0.756795 | 1.055422 |
| 13 | 59.0 | 3481.0 | 0.9003444 | 0.7557987 | 1.072534 |
| 14 | 69.0 | 4761.0 | 0.9262595 | 0.7774887 | 1.103497 |
| 15 | 22.0 | 484.0 | 0.9749867 | 0.9429911 | 1.008068 |
| 16 | 50.0 | 2500.0 | 0.8937215 | 0.756795 | 1.055422 |
| 17 | 59.0 | 3481.0 | 0.9003444 | 0.7557987 | 1.072534 |
| 18 | 69.0 | 4761.0 | 0.9262595 | 0.7774887 | 1.103497 |
| 19 | 18.0 | 324.0 | 1.0006922 | 0.9997862 | 1.001599 |
| 20 | 40.0 | 1600.0 | 0.9044837 | 0.7869341 | 1.039593 |
| 21 | 46.0 | 2116.0 | 0.8957255 | 0.7648637 | 1.048977 |
| 22 | 59.0 | 3481.0 | 0.9003444 | 0.7557987 | 1.072534 |
| 23 | 18.0 | 324.0 | 1.0006922 | 0.9997862 | 1.001599 |
| 24 | 40.0 | 1600.0 | 0.9044837 | 0.7869341 | 1.039593 |
| 25 | 46.0 | 2116.0 | 0.8957255 | 0.7648637 | 1.048977 |
| 26 | 59.0 | 3481.0 | 0.9003444 | 0.7557987 | 1.072534 |

**Table S5.** Specific Serum LDL Levels and Associated Odds Ratios

| Index | Dose (mg/dL) | Dose² | Odds Ratio | 95% CI (Lower) | 95% CI (Upper) |
| --- | --- | --- | --- | --- | --- |
| 1 | 96 | 9216 | 1.0 | 1.0 | 1.0 |
| 2 | 119 | 14161 | 0.995119 | 0.9159849 | 1.08109 |
| 3 | 136 | 18496 | 0.9898977 | 0.878828 | 1.115005 |
| 4 | 154 | 23716 | 0.9829007 | 0.8531157 | 1.13243 |
| 5 | 181 | 32761 | 0.9696451 | 0.8100384 | 1.1607 |
| 6 | 61 | 3721 | 1.0025407 | 0.8096625 | 1.241366 |
| 7 | 136 | 18496 | 0.9898977 | 0.878828 | 1.115005 |
| 8 | 164 | 26896 | 0.9783723 | 0.8402059 | 1.139259 |
| 9 | 195 | 38025 | 0.961507 | 0.7696441 | 1.201199 |
| 10 | 61 | 3721 | 1.0025407 | 0.8096625 | 1.241366 |
| 11 | 136 | 18496 | 0.9898977 | 0.878828 | 1.115005 |
| 12 | 164 | 26896 | 0.9783723 | 0.8402059 | 1.139259 |
| 13 | 195 | 38025 | 0.961507 | 0.7696441 | 1.201199 |
| 14 | 60 | 3600 | 1.0025261 | 0.8034065 | 1.250996 |
| 15 | 136 | 18496 | 0.9898977 | 0.878828 | 1.115005 |
| 16 | 165 | 27225 | 0.9778946 | 0.838813 | 1.140037 |
| 17 | 196 | 38416 | 0.9608935 | 0.7661266 | 1.205175 |
| 18 | 60 | 3600 | 1.0025261 | 0.8034065 | 1.250996 |
| 19 | 136 | 18496 | 0.9898977 | 0.878828 | 1.115005 |
| 20 | 165 | 27225 | 0.9778946 | 0.838813 | 1.140037 |
| 21 | 196 | 38416 | 0.9608935 | 0.7661266 | 1.205175 |

**Table S6**. Meta-regression anallysis for Cholesterol and CRC risk.

| **Moderator** | **Intercept_HR** | **Intercept_CI_lb** | **Intercept_CI_ub** | **Moderator_HR** | **Moderator_CI_lb** | **Moderator_CI_ub** | **p** | **I2** |
| --- | --- | --- | --- | --- | --- | --- | --- | --- |
| Sex | 1.31503 | 0.956732 | 1.807512 | 0.669045 | 0.386526 | 1.158062 | 0.151071 | 32.46004 |
| Age | 0.325761 | 0.062659 | 1.69361 | 1.020971 | 0.992196 | 1.05058 | 0.154774 | 33.12764 |
| Follow_up | 1.151742 | 0.515413 | 2.573682 | 0.995341 | 0.939589 | 1.054402 | 0.873845 | 57.09648 |
| BMI | 26.69106 | 0.001114 | 639578.5 | 0.887776 | 0.607796 | 1.29673 | 0.538036 | 55.00133 |

**Table S7**. Meta-regression anallysis for Cholesterol and colon caner risk.

| **Moderator** | **Intercept_HR** | **Intercept_CI_lb** | **Intercept_CI_ub** | **Moderator_HR** | **Moderator_CI_lb** | **Moderator_CI_ub** | **p** | **I2** |
| --- | --- | --- | --- | --- | --- | --- | --- | --- |
| Sex | 0.989110602 | 0.73143609 | 1.337560174 | 1.228341666 | 0.729499798 | 2.068298375 | 0.439155464 | 0 |
| Follow_up | 1.203544242 | 1.007204986 | 1.438156844 | 0.994156416 | 0.981933199 | 1.006531789 | 0.353135981 | 0 |

**Table S8**. Meta-regression anallysis for Cholesterol and rectum caner risk.

| **Moderator** | **Intercept_HR** | **Intercept_CI_lb** | **Intercept_CI_ub** | **Moderator_HR** | **Moderator_CI_lb** | **Moderator_CI_ub** | **p** | **I2** |
| --- | --- | --- | --- | --- | --- | --- | --- | --- |
| Sex | 1.478111 | 0.843564 | 2.589978 | 0.691262 | 0.266159 | 1.79533 | 0.448295 | 29.38045 |
| Follow_up | 1.210961 | 0.883248 | 1.660267 | 0.999203 | 0.976074 | 1.02288 | 0.946798 | 31.00503 |

**Table S9**. Meta-regression anallysis for TG and CRC risk.

| **Moderator** | **Intercept_HR** | **Intercept_CI_lb** | **Intercept_CI_ub** | **Moderator_HR** | **Moderator_CI_lb** | **Moderator_CI_ub** | **p** | **I2** |
| --- | --- | --- | --- | --- | --- | --- | --- | --- |
| Sex | 1.094225 | 0.875258 | 1.367974 | 1.0309 | 0.708069 | 1.500921 | 0.873836 | 0 |
| Age | 1.037758 | 0.363692 | 2.96113 | 1.00134 | 0.98158 | 1.021498 | 0.89522 | 0 |
| Follow_up | 1.220803 | 0.629761 | 2.366546 | 0.991036 | 0.93163 | 1.054231 | 0.775259 | 0 |

**Table S10**. Meta-regression anallysis for TG and colon cancer risk.

| **Moderator** | **Intercept_HR** | **Intercept_CI_lb** | **Intercept_CI_ub** | **Moderator_HR** | **Moderator_CI_lb** | **Moderator_CI_ub** | **p** | **I2** |
| --- | --- | --- | --- | --- | --- | --- | --- | --- |
| Sex | 1.850208 | 1.194715 | 2.865343 | 0.50508 | 0.249332 | 1.023155 | 0.057901 | 17.5 |
| Follow_up | 1.541702 | 0.622534 | 3.818019 | 0.983749 | 0.921452 | 1.050258 | 0.623511 | 57.15058 |

**Table S11**. Meta-regression anallysis for HDL and CRC risk.

| **Moderator** | **Intercept_HR** | **Intercept_CI_lb** | **Intercept_CI_ub** | **Moderator_HR** | **Moderator_CI_lb** | **Moderator_CI_ub** | **p** | **I2** |
| --- | --- | --- | --- | --- | --- | --- | --- | --- |
| Sex | 1.070519 | 0.735259 | 1.55865 | 0.794579 | 0.447075 | 1.412192 | 0.433223 | 41.85635 |
| Age | 1.19219 | 0.216941 | 6.551641 | 0.995195 | 0.964411 | 1.026961 | 0.763836 | 50.17297 |
| Follow_up | 0.838916 | 0.529442 | 1.329288 | 1.011489 | 0.972123 | 1.05245 | 0.572728 | 18.24334 |

**Table S12**. Semsitivity anallysis for Cholesterol and CRC risk.

| **Study** | **Estimate** | **CI_lb** | **CI_ub** | **p_value** | **Tau2** | **I2** |
| --- | --- | --- | --- | --- | --- | --- |
| 1 | 1.114531 | 0.919633 | 1.350733 | 0.268868 | 0.024573 | 54.76212 |
| 2 | 1.140358 | 0.938421 | 1.38575 | 0.186563 | 0.019437 | 41.19625 |
| 3 | 1.062092 | 0.852212 | 1.323661 | 0.591759 | 0.034502 | 60.80917 |
| 4 | 1.052132 | 0.852966 | 1.297804 | 0.635052 | 0.029967 | 57.75844 |
| 5 | 1.104419 | 0.853035 | 1.429885 | 0.451026 | 0.045011 | 53.89274 |
| 6 | 1.009033 | 0.904469 | 1.125685 | 0.87201 | 1.27E-07 | 0.000511 |

**Table S13**. Semsitivity anallysis for TG and colon cancer risk.

| **Study** | **Estimate** | **CI_lb** | **CI_ub** | **p_value** | **Tau2** | **I2** |
| --- | --- | --- | --- | --- | --- | --- |
| 1 | 1.298515 | 1.014533 | 1.661988 | 0.038028 | 0.034475 | 56.18922 |
| 2 | 1.371321 | 1.166002 | 1.612796 | 0.000136 | 1.18E-06 | 0.003754 |
| 3 | 1.210866 | 0.90706 | 1.616428 | 0.194238 | 0.052101 | 62.35877 |
| 4 | 1.158989 | 0.894424 | 1.501812 | 0.264409 | 0.031171 | 45.1792 |
| 5 | 1.18339 | 0.932234 | 1.502212 | 0.166531 | 0.033171 | 56.97925 |

**Table S14**. Semsitivity anallysis for TG and colon cancer risk.

| **Study** | **Estimate** | **CI_lb** | **CI_ub** | **p_value** | **Tau2** | **I2** |
| --- | --- | --- | --- | --- | --- | --- |
| 1 | 0.977866 | 0.559217 | 1.70993 | 0.937429 | 0.189397 | 78.47719 |
| 2 | 0.962264 | 0.554476 | 1.669959 | 0.891219 | 0.18075 | 77.02338 |
| 3 | 1.275422 | 1.007606 | 1.614421 | 0.043076 | 0 | 0 |
| 4 | 0.936216 | 0.532334 | 1.646525 | 0.819019 | 0.172327 | 69.23838 |

| **Table S15.** Excluded studies. | | |
| --- | --- | --- |
| First Author et al. | Title article | Reason for excluding |
| Pikul Laisupasin et al. | Comparison of Serum Lipid Profiles between Normal Controls and Breast Cancer Patients | Not CRC risk as outcome |
| Chacko Sara A et al. | Serum 25-hydroxyvitamin D concentrations in relation to cardiometabolic risk factors and metabolic syndrome in postmenopausal women | Not CRC risk as outcome |
| Dandan Li et al. | Comparative analysis of the serum proteome profiles of thyroid cancer: An initial focus on the lipid profile | Not CRC risk as outcome |
| Arti Verma et al. | Association between Lipid Profile and Ovarian Cancer in women ofNorth India | Not CRC risk as outcome |
| Anne-Sofie Furber et al. | Metabolic and Hormonal Profiles: HDL Cholesterol as a Plausible Biomarker of Breast Cancer Risk. The Norwegian EBBA Study | Not CRC risk as outcome |
| Christoph Nowak et al. | A Mendelian randomization study of the effects of blood lipids on breast cancer risk | Not CRC risk as outcome |
| Paul M Ridker et al | Non–HDL Cholesterol, Apolipoproteins A-I and B100, Standard Lipid Measures, Lipid Ratios, and CRP as Risk Factors for Cardiovascular Disease in Women | Not CRC risk as outcome |
| Jiyoung Ahn et al. | Prediagnostic Total and High-Density Lipoprotein Cholesterol and Risk of Cancer | Not CRC risk as outcome |
| Kumar et al. | Serum lipid profile in oral cancer and leukoplakia Correlation with tobacco abuse and histological grading | Not CRC risk as outcome |
| Geoffrey C. Kabat et al. | Serum lipids and risk of obesity-related cancers in postmenopausal women | Not CRC risk as outcome |
| Rigter et al. | Gastric adenocarcinoma in patients treated for Hodgkin/testicular cancer | Not CRC risk as outcome |
| Rimola et al. | Lysophospholipids Contribute to Oxaliplatin-Induced Acute Peripheral Pain | Not CRC risk as outcome |
| Rysz et al. | The Role of Metabolic Factors in Renal Cancers | Not CRC risk as outcome |
| Safaroghli-Azar et al. | PI3K classes: From cell signaling to autophagy | Not CRC risk as outcome |
| Safi et al. | Lipid droplets provide metabolic flexibility for cancer progression | Not CRC risk as outcome |
| Saito et al. | Plasma lipid markers and machine learning-based algorithm for gastric cancer | Not CRC risk as outcome |
| Sakamoto et al. | MUTYH and hepatocarcinogenesis in NASH model | Not CRC risk as outcome |
| Samec et al. | Flavonoids attenuate cancer metabolism by modulating lipid metabolism,and use of amino acids or ketone bodies | Not CRC risk as outcome |
| Sánchez Marín et al | Drug repositioning in thyroid cancer: from point mutations to gene fusions | Not CRC risk as outcome |
| Sandhu et al. | Chickpea Nutritional Status and Value Chain for Sustainable Development | Not CRC risk as outcome |
| Sasaki et al. | Gangliosides as signaling regulators in cancer | Not CRC risk as outcome |
| Sato et al. | The association between sarcopenia and endotoxin in patients with alcoholic cirrhosis | Not CRC risk as outcome |
| Saxena et al. | Incidental diagnosis of intestinal perforation on a 99mTc DTPA renogram | Not CRC risk as outcome |
| Schei-Andersen et al. | Histopathological phenotyping in PTEN Hamartoma Tumor Syndrome | Not CRC risk as outcome |
| [Schniers et al.](javascript:;) | Deletion of Slc6a14 reduces cancer growth in KPC pancreatic cancer model | Not CRC risk as outcome |
| Schumacher et al. | Exploring the inhibitory potential of the antiarrhythmic drug amiodarone against Clostridioides difficile toxins TcdA and TcdB | Not CRC risk as outcome |
| Seo et al. | High-Precision Synthesis of RNA-Loaded Lipid Nanoparticles for Biomedical Applications | Not CRC risk as outcome |
| Setayeshpour et | Environmental Determinants of Ferroptosis in Cancer | Not CRC risk as outcome |
| Shaheen et al. | Screening Practices, Knowledge and Adherence Among Health Care Professionals | Not CRC risk as outcome |
| Sharma et al. | Recent advances in lipid-based long-acting injectable depot formulations | Not CRC risk as outcome |
| Shende and Gupta | Role of Lipopolysaccharides in Nanocarrier Systems | Not CRC risk as outcome |
| Shi et al. | lncRNAs and mRNAs expression in HCC – predictive value | Not CRC risk as outcome |
| Shi et al. | Bile Acids, Intestinal Barrier Dysfunction, and Related Diseases | Not CRC risk as outcome |
| Shi et al. | Ferroptosis-Based Therapeutic Strategies for Precision Medicine in Cancer | Not CRC risk as outcome |
| Shimon et al. | The Big Potential of Small Particles: Lipid-Based Nanoparticles and Exosomes in Vaccination | Not CRC risk as outcome |
| Rossouw et al. | Risks and Benefits of Estrogen/Progestin in Healthy Women (WHI)-2002 | Not CRC risk as outcome |
| Shen et al. | Fatty acid metabolism-related lncRNA signatures as a novel prognostic model for clear cell renal cell carcinoma | Not CRC risk as outcome |
| Shebbo et al. | Hepatoprotective effect of Matricaria chamomilla against carcinogenic hepatic damage | Not CRC risk as outcome |
| Al-Hawary et al. | Curcumin in liver cancer: mechanisms and nanoformulations | Not CRC risk as outcome |
| Savukaitytė et al. | DDIT4 Downregulation... Fatty Acid Metabolism in Breast Cancer Cells | Not CRC risk as outcome |
| Schniers et al. | Deletion of Slc6a14 reduces cancer growth in KPC pancreatic cancer model | Not CRC risk as outcome |
| Ruan et al. | CD36: an emerging therapeutic target for cancer and its molecular mechanisms | Not CRC risk as outcome |
| Mathilde His et al. | Prospective associations between serum biomarkers of lipid metabolism and overall, breast and prostate cancer risk | Not CRC risk as outcome |
| Daniela Guardado-Félix et al. | Chickpea (Cicer arietinum L.) sprouts containing supranutritional levels of selenium decrease tumor growth of colon cancer cells xenografted in immune-suppressed mice | Not CRC risk as outcome |
| Shi et al. | Clinical Mass Spectrometry Workflow for Metabolomics Studies | Not CRC risk as outcome |
| Seo et al. | High-Precision Synthesis of RNA-Loaded Lipid Nanoparticles for Biomedical Applications | Not CRC risk as outcome |
| Shoeibi et al. | Enhancing Healthcare Outcomes via Nano-Phytosomal Delivery of Allium ampeloprasum | Not CRC risk as outcome |
| Ramirez et al. | Access to Care Among Adults with Limited English Proficiency | Not lipid as exposure |
| Rauf et al. | Garlic (Allium sativum): Chemistry and anticancer properties | Not lipid as exposure |
| Ren et al. | Ferroptosis and EMT: key targets for combating Ferroptosis and EMT: key targets for combating cancer progression and therapy resistance | Not lipid as exposure |
| Rhoades et al. | Cancer mortality in a population-based cohort of American Indians – The strong heart study | Not lipid as exposure |
| Rincón-Riveros et al. | Regulation of antitumor immune responses by exosomes from tumor/immune cells | Not lipid as exposure |
| Rodriguez and Coveñas | Biochemical Mechanisms Associating Alcohol Use Disorders with Cancers | Not lipid as exposure |
| Rumgay et al. | Alcohol and cancer: Epidemiology and biological mechanisms | Not lipid as exposure |
| Ryu et al. | Gene editing particle system for drug-resistant colorectal cancer | Not lipid as exposure |
| Sak | Radiosensitizing Potential of Curcumin in Different Cancer Models | Not lipid as exposure |
| [Santos and Almeida](https://pubmed.ncbi.nlm.nih.gov/?term=Almeida+F&cauthor_id=32545155) | Role of Exosomal miRNAs and the Tumor Microenvironment in Drug Resistance | Not lipid as exposure |
| Sargazi et al. | Relationship Between CASP9 and CASP10 Polymorphisms and Cancer Susceptibility | Not lipid as exposure |
| Savva et al. | Progress with Metabolomic Blood Tests for GI Cancer Diagnosis | Not lipid as exposure |
| Scavo et al. | Exosomes for Diagnosis and Therapy in GI Cancers | Not lipid as exposure |
| Scheetz et al. | Synthetic HDL Nanoparticles Delivering Docetaxel and CpG for Colon Adenocarcinoma | Not lipid as exposure |
| Malin Backman et al. | A randomized pilot study with daily walking during adjuvant chemotherapy for patients with breast and colorectal cancer | Not lipid as exposure |
| Shi et al. | Exosomes and ferroptosis: roles in tumor regulation | Not lipid as exposure |
| Shi et al. | Ferroptosis: Biochemistry and Biology in Cancers | Not lipid as exposure |
| Shoeibi et al. | Enhancing Healthcare Outcomes via Nano-Phytosomal Delivery of Allium ampeloprasum | Not lipid as exposure |
| Rong Xu et al. | A genome-wide systems analysis reveals strong link between colorectal cancer and trimethylamine N-oxide (TMAO), a gut microbial metabolite of dietary meat and fat | Not lipid as exposure |
| Annie S Anderson et al. | The impact of a bodyweight and physical activity intervention (BeWEL) initiated through a national colorectal cancer screening programme: randomised controlled trial | Not lipid as exposure |
| Shi et al. | Ferroptosis-Based Therapeutic Strategies for Precision Medicine in Cancer | Not lipid as exposure |
| Rosa Divella et al. | ADIPOQ rs266729 G/C gene polymorphism and plasmatic adipocytokines connect metabolic syndrome to colorectal cancer | Not lipid as exposure |
| Huijuan Zhu et al. | Serum and Adipose Tissue mRNA Levels of ATF3 and FNDC5/Irisin in Colorectal Cancer Patients With or Without Obesity | Not lipid as exposure |
| Jie You et al. | Nonalcoholic Fatty Liver Disease:A Negative Risk Factor for Colorectal Cancer Prognosis | Not lipid as exposure |
| Gad Rennert et al. | Use of Bisphosphonates and Reduced Risk of Colorectal Cancer | Not lipid as exposure |
| Relucenti et al. | The Ultrastructural Analysis of Human CRC Stem Cell-Derived Spheroids and Their Mouse Xenograft Shows That the Same Cells Types Have Different Ratios | Not lipid as exposure |
| Salari et al. | Hyaluronic acid-based drug nanocarriers: A systematic review | Review |
| Salari et al. | ACT therapy on anxiety and depression in cancer patients: A systematic review | Review |
| Schwingshackl et al. | Total Dietary Fat Intake, Fat Quality, and Health Outcomes: A Scoping Review | Review |
| Rasilla et al. | 18F-FDG PET-CT in colorectal cancer. Where are we going? | Review |
| Alpers et al. | Garlic and its potential for prevention of colorectal cancer and other conditions | Review |
| Liwen Ren et al. | Apolipoproteins and cancer | Review |
| Guifang Yan | Lipidome in colorectal cancer | Review |
| Pingting Zhou | Prognostic role of serum total cholesterol and high-density lipoprotein cholesterol in cancer survivors: A systematic review and meta-analysis | Review |
| Yun Tian et al. | The association between serum lipids and colorectal neoplasm: a systemic review and meta-analysis | Review |
| Alicja Pakiet et al. | Changes in lipids composition and metabolism in colorectal cancer: a review | Review |

| **Table S16.** List of fundings of the included studies. | | |
| --- | --- | --- |
| **Authors** | **Year** | **Funding** |
| Hsu et al. | 2022 | Fu Jen Catholic University (A0110183) |
| [Tong Liu et al.](https://link.springer.com/article/10.1007/s00011-022-01597-9#auth-Tong-Liu-Aff1-Aff2-Aff3) | 2022 | National Key Research and Development Program (No. 2017YFC1309200) and the Beijing Municipal Science and Technology Commission (SCW2018-06) to Dr. Hanping Shi |
| Zhang et al. | 2022 | UM1 CA186107, U01 CA176726, P01 CA87969,U01 CA167552 fromNIH. NIH grants R01 CA137178 and R35 CA253185 (A.T. Chan). World Cancer Research Fund (E.L. Giovannucci). |
| Zhe Fang et al. | 2021 | American Cancer Society (MRSG-17-220-01-NEC to M.S.) and the U.S. National Institutes of Health (R00 CA215314 to M.S.).Wellcome Trust.Medical Research Council.the United Kingdom Department of Health, the Scottish Government, the Welsh Assembly Government, the British Heart Foundation, and Diabetes UK. |
| Xin Li et al. | 2019 | National Key R&D Program of China (grant numbers 2018YFC1315000/2018YFC1315001, 2016YFC1302500/2016YFC1302503), CAMS Innovation Fund for Medical Sciences (grant numbers 2017-I2M-1-006, 2019-I2M-2-002), the Training Programme Foundation for the Talents in Beijing City (grant number 2017000021223TD05), Beijing Municipal Science and Technology Project (grant number D171100002617001), the and National Natural Science Foundation of China (grant number 81673265). |
| [Verena Andrea Katzke et al.](https://link.springer.com/article/10.1186/s12916-017-0976-4#auth-Verena_Andrea-Katzke-Aff1) | 2017 | Helmholtz Association (Portfolio Theme “Metabolic Dysfunction”) and the German Federal Ministry of Education and Research (BMBF) (Grant number 01ER0809) and co-funded by the German Center for Lung Research (DZL, grant PB13394). |
| Chandler Paulette D et al. | 2016 | The NIH (grants CA-047988, HL-043851, HL-080467, HL-099355, and UM1 CA182913; to the Women’s Health Study), the National Cancer Institute (grant U01CA138962; to PDC), and the American Cancer Society (grant 127524-MRSG-15-012-01-CNE; to PDC). |
| Taulant Muka et al. | 2016 | Erasmus Mundus Western Balkans (ERAWEB). TM, BK, JCK and work in ErasmusAge, a centre for ageing research across the life course founded by Nestlé Nutrition (Nestec Ltd.), Metagenics Inc and AXA |
| [Yunxia Lu et al.](https://pubmed.ncbi.nlm.nih.gov/?term=Lu+Y&cauthor_id=26511906) | 2015 | The Swedish Society of Medicine and the Karolinska Foundation. |
| Aesun Shin et al. | 2014 | The authors have no support or funding to report. |
| Susanne Strohmaier et al. | 2013 | World Cancer Research Fond International (2007/09 and 2010/247 to P.S.); and Medical University of Innsbruck (MUISTART). |
| Cari M Kitahara et al. | 2011 | Grant No. 10526 from Korean Seoul City Research and Grant No. 0920330 from the National Research and Development Program for Cancer Control, Ministry for Health, Welfare and Family Affairs, Republic of Korea. This study was also supported in part by the Intramural Research Program of the National Cancer Institute, National Institutes of Health. |
| Hiroyasu Iso et al. | 2009 | Grants in aid for Cancer Research and for the Third Term Comprehensive Ten-Year Strategy for Cancer Control from the Ministry of Health, Labor and Welfare of Japan. |
| [Wegene Borena et al.](https://pubmed.ncbi.nlm.nih.gov/?term=Borena+W&cauthor_id=21140204) | 2011 | World Cancer Research Fund International (WCRF International) 2007/2009 and the Austrian National Bank Grant OENB-12737 (to H.U.). |
| Jiyoung Ahn et al. | 2009 | Intramural Research Program of the National Institutes of Health, Division of Cancer Epidemiology and Genetics, National Cancer Institute, Department of Health and Human Services with Public Health Service contracts N01-CN-45165, N01-RC-45035, and N01-RC-37004. |
| Manami Inoue et al. | 2009 | Grant-in-Aid for Cancer Research, for the Third Term Comprehensive Control Research for Cancer, for Research on Hepatitis, and for Impact of metabolic factors on cancer risk Inoue et al. 245 Health Science Research from the Ministry of Health, Labour and Welfare of Japan. |
| Rehana L Ahmed et al. | 2006 | The Atherosclerosis Risk in Communities Study isa collaborative study supported by National Heart,Lung, and Blood Institute (contracts N01-HC-55015, 55016, 55018, 55019, 55020, 55021, and55022). In addition, this work was supported bythe National Cancer Institute (Grant R03-CA65473). R.L.A. was supported by the NationalInstitutes of Health (Grants T32 GM08244-15 andT32 CA09607-15) and by the University of Minne-sota Thomas Shevlin predoctoral fellowship. |
| [Matthew Tsushima et al.](https://pubmed.ncbi.nlm.nih.gov/?term=Tsushima+M&cauthor_id=15810632) | 2005 | Grant RO1 CA 33644 from the National Cancer Institute, National Institutes of Health. |
| R E Schoen et al. | 1999 | Public Health Service (PHS) grant K07CA72561 (to R. E. Schoen) from the National Cancer Institute, National Institutes of Health (NIH), Department of Health and Human Services (DHHS);and by PHS contract N01HC85079 from the National Heart, Lung, and Blood Institute, NIH, DHHS |
| [M Gaard et al.](https://pubmed.ncbi.nlm.nih.gov/?term=Gaard+M&cauthor_id=9051877) | 1997 | Grant 93020/002 from the Norwegian Cancer Society. |
| P H Chyou et al. | 1996 | Supported in part by grant ROI CA 33644 from the National Cancer Institute. |
| A Schatzkin et al. | 1988 | National Institute on Aging; National Center for Health Statistics; National Cancer Institute; National Heart, Lung, and Blood Institute; National Institute of Arthritis, Diabetes, and Digestive and Kidney Diseases; National Institute of Mental Health; National Institute of Alcohol Abuse and Alcoholism; National Institute of Allergy and Infectious Disease; and National Institute of Neurological and Communicative Disorders and Stroke. The field work was conducted by Westat, Inc., under Contract No. 23380-2049. |
| S A Törnberg et al. | 1986 | Grant (83:83) from the Cancer Society in Stockholm and by a grant (83:508) from the King Gustaf V Jubilee Fund. |

**Methods Appendix:**

The following deviations from the PROSPERO-registered protocol (ID: CRD42024612076) occurred due to limitations in the data available from the included studies:

1. **Stratified Analysis by Age Groups (<50 vs. ≥50 Years)**

*Planned:* Subgroup analysis by participant age to explore differences in the association between lipid levels and colorectal cancer risk.

*Deviation:* The included studies did not provide stratified effect estimates by age groups (e.g., <50 vs. ≥50 years). Most reported only mean or median ages without age-specific hazard ratios, making this analysis unfeasible.

- **Subgroup Analysis by Study Design (Cohort vs. Case-Control Studies)**

*Planned:* Comparison of effect estimates between cohort and case-control studies to assess potential design-related biases.

*Deviation:* Only prospective cohort studies were included in the meta-analysis, as case-control studies were excluded per the pre-defined eligibility criteria. Thus, this comparison could not be performed.

**Statistical Analysis Appendix:**

In studies where the reference group was not the lowest exposure category (e.g., where the third quintile was the reference instead of the first), we recalculated the hazard ratios to reflect comparisons between the highest and lowest categories (Q5 vs. Q1). This was done by applying the logarithmic transformation of the reported ratios. Specifically, when a study reported the hazard ratio (HR) for the fifth quintile versus the third quintile (Q5 vs. Q3) and for the first quintile versus the third quintile (Q1 vs. Q3), we calculated the log-transformed ratio for Q5 vs. Q1 using the formula:
    **log(HRQ5vsQ1) = log(HRQ5vsQ3) – log(HRQ1vsQ3)**
The exponential of this value was then taken to retrieve the adjusted HR or OR for Q5 vs. Q1:
    **HRQ5vsQ1 = exp(log(HRQ5vsQ3)– log(HRQ1vsQ3))**

To compute the variance and 95% confidence intervals of the transformed HR/OR, we summed the variances of the log-transformed ratios:
    **Var(log(HRQ5vsQ1)) = Var(log(HRQ5vsQ3)) + Var(log(HRQ1vsQ3))**
Then, the standard error (SE) was derived as the square root of this variance, and the 95% confidence interval (CI) was calculated as:
    **CI = exp[log(HRQ5vsQ1) ± 1.96 × SE]**

This approach allowed consistent high-versus-low comparisons across studies, regardless of the originally reported reference categories.
